# Interrogating Genomic-Scale Data to Resolve Recalcitrant Nodes in the Spider Tree of Life

**DOI:** 10.1093/molbev/msaa251

**Published:** 2020-09-28

**Authors:** Siddharth Kulkarni, Robert J Kallal, Hannah Wood, Dimitar Dimitrov, Gonzalo Giribet, Gustavo Hormiga

**Affiliations:** 1 Department of Biological Sciences, The George Washington University, Washington, DC; 2 Department of Entomology, National Museum of Natural History, Smithsonian Institution, Washington, DC; 3 Department of Natural History, University Museum of Bergen, University of Bergen, Bergen, Norway; 4 Museum of Comparative Zoology, Department of Organismic and Evolutionary Biology, Harvard University, Cambridge, MA

**Keywords:** Araneae, noncoding regions, phylogeny, target-capture, transcriptomics

## Abstract

Genome-scale data sets are converging on robust, stable phylogenetic hypotheses for many lineages; however, some nodes have shown disagreement across classes of data. We use spiders (Araneae) as a system to identify the causes of incongruence in phylogenetic signal between three classes of data: exons (as in phylotranscriptomics), noncoding regions (included in ultraconserved elements [UCE] analyses), and a combination of both (as in UCE analyses). Gene orthologs, coded as amino acids and nucleotides (with and without third codon positions), were generated by querying published transcriptomes for UCEs, recovering 1,931 UCE loci (*codingUCEs*). We expected that congeners represented in the *codingUCE* and UCEs data would form clades in the presence of phylogenetic signal. Noncoding regions derived from UCE sequences were recovered to test the stability of relationships. Phylogenetic relationships resulting from all analyses were largely congruent. All nucleotide data sets from transcriptomes, UCEs, or a combination of both recovered similar topologies in contrast with results from transcriptomes analyzed as amino acids. Most relationships inferred from low-occupancy data sets, containing several hundreds of loci, were congruent across Araneae, as opposed to high occupancy data matrices with fewer loci, which showed more variation. Furthermore, we found that low-occupancy data sets analyzed as nucleotides (as is typical of UCE data sets) can result in more congruent relationships than high occupancy data sets analyzed as amino acids (as in phylotranscriptomics). Thus, omitting data, through amino acid translation or via retention of only high occupancy loci, may have a deleterious effect in phylogenetic reconstruction.

## Introduction

Massive parallel sequencing and the exponential increase in the size of data sets have enabled researchers to use a variety of genomic data types (whole genomes, transcribed gene regions, introns, fast/slow evolving loci, etc.) to address specific evolutionary questions. These data sets have rapidly dwarfed Sanger sequencing-based studies in terms of amounts of data (Mardis 2011), however, they have proven to be challenging to analyze. Once celebrated as the gold standard for inferring evolutionary histories (Gee 2003; Rokas et al. 2003), it is now clear that sheer quantity of data will not unequivocally resolve all problematic nodes in a phylogeny. Conflicting but highly supported phylogenetic relationships have emerged in many data sets, even when containing hundreds or thousands of loci.

Furthermore, the objective quantification of branch support is obfuscated by widespread reliance on the bootstrap support metric (in a maximum likelihood framework), among a few others like posterior probability in a Bayesian framework. Bootstrap values are often inflated when comparable numbers of sites indicate conflicting relationships for a given branch (Felsenstein 1985). Such conflicts are common among large-scale data sets and therefore bootstrap values are generally high. This conundrum has impacted phylogenetic studies of many groups of organisms, including birds (Jarvis et al. 2014; Prum et al. 2015; Walker et al. 2018; Cloutier et al. 2019), placental mammals (Morgan et al. 2013; Romiguier et al. 2013), extant angiosperms (Zanis et al. 2002; Wickett et al. 2014; Xi et al. 2014), and arachnids (e.g., Sharma et al. 2014; Ballesteros and Sharma 2019; Lozano-Fernández et al. 2019). In the present study, we focus on the nature of the systematic conflict (with high bootstrap support for alternative hypotheses) across genomic data sets addressing a yet to be satisfactorily resolved problem in spider phylogenetics.

In recent studies on the spider tree of life, phylogenies resulting from the analysis of either transcriptomes or ultraconserved elements (UCEs) have largely converged on similar topologies (e.g., Garrison et al. 2016; Fernández et al. 2018; Kulkarni et al. 2020; Dimitrov and Hormiga 2021; Kallal et al. forthcoming). However, incongruence persists in some recalcitrant nodes, receiving high support for contradicting hypotheses. Some of these incongruences, in the context of spider systematics, include: 1) the placement of the RTA Clade (a group of spiders characterized by the presence of a retrolateral tibial apophysis in the male palp–the appendage that male spiders use for copulation) with respect to the “UDOH grade” (an assemblage containing the spider families Uloboridae, Deinopidae, Oecobiidae, and Hersiliidae); 2) the placement of Nicodamoidea with respect to Araneoidea (the ecribellate orb weavers); and, 3) the interfamilial relationships of the miniature orb-weaving families—a group informally known as “symphytognathoids.” The “symphytognathoids” (Griswold et al. 1998) include the families Anapidae, Mysmenidae, Theridiosomatidae, and Symphytognathidae (which includes the smallest adult spider in the world, *Patu digua*; Forster and Platnick 1977). Few studies have found support for the monophyly of “symphytognathoids,” and a particular study suggests that Synaphridae also belongs to this group (Lopardo et al. 2011). Here, we focus on the relationships of the “symphytognathoid” families as a major area of conflict in the spider tree of life by comparing a diversity of approaches and data classes and their effects on this particular topology.

The monophyly of “symphytognathoid” families has been supported, although not formalized as a taxon, by morphological and behavioral characters (Griswold et al. 1998; Schütt 2003; Lopardo and Hormiga 2008; Lopardo et al. 2011; Hormiga and Griswold 2014), but these families have appeared as either paraphyletic or polyphyletic in molecular phylogenies based on standard Sanger markers (Dimitrov et al. 2017; Wheeler et al. 2017) or transcriptomes (Fernández et al. 2018; Kallal et al. forthcoming). Lopardo et al.’s (2011) extensive Sanger-based data set supported “symphytognathoid” monophyly only when the nucleotide data were analyzed in combination with phenotypic data. Recently, an analysis using target enrichment methods to capture UCEs provided the first molecular support for the monophyly of “symphytognathoids” (ultrafast bootstrap >95), although only with the analyzed low-occupancy data sets (Kulkarni et al. 2020). This result was surprising, given the lack of support for symphytognathoid monophyly in all prior molecular analyses, including phylogenomic data sets analyzed as amino acid data in a maximum likelihood framework (Kallal et al. forthcoming). In that study, the parsimony analysis of the amino acid data set recovered Theridiosomatidae as the sister group of Araneidae, with the remaining “symphytognathoids” forming a monophyletic group (Kallal et al. forthcoming).

The paradox of highly supported but incongruent relationships requires a critical assessment of the nature of the data being analyzed, in our case, in the context of the high bootstrap support for both, the monophyly or polyphyly of “symphytognathoids” in different analyses. The phylogenetic relationships of the miniature orb weavers offer an excellent system to explore the nature of conflict between these two types of genomic data sets. One possible approach, albeit unexplored up to this point, is to identify the phylogenetic signal common to transcriptomic and UCE data sets. Transcriptomes, which are sequenced from mRNA, are often analyzed as amino acids, and include only exonic regions. UCEs on the other hand are sequenced from the genome and are typically analyzed as nucleotides, and include both exons and noncoding regions. The possibility of combining the vast data sets of UCEs and transcriptomes would not only enable an expanded taxon sampling but also allow reconciliation of the existing UCE and transcriptome data sets (Bossert et al. 2019). Furthermore, because a recent study has shown that currently sequenced UCEs in Arachnida are mostly exonic (Hedin et al. 2019) it should be possible to combine UCEs and transcriptomes in a meaningful manner (Bossert et al. 2019; Hedin et al. 2019).

The present study aims to identify the causes of incongruence among transcriptome-based and UCE-based sequences in phylogenetic analyses of spiders by leveraging data from recent studies (e.g., Garrison et al. 2016; Fernández et al. 2018; Kulkarni et al. 2020; Kallal et al. forthcoming). Our approach was to reconstruct phylogenies using sequences from transcriptomes, UCEs, and a combination of data sources, at both the amino acid and nucleotide levels. We then analyzed these data sets using different phylogenetic methods at different occupancy levels, while also exploring the phylogenetic signal of noncoding regions, something rarely attempted in this kind of phylogenetic analyses.

First, we hypothesize that transcriptomes contain ultraconserved regions. On targeting these coding ultraconserved regions using the Spider2Kv1 probe set (Kulkarni et al. 2020), we reconstruct a phylogeny to resolve a number of selected recalcitrant nodes. The efficacy of the transcriptome-derived UCEs for resolving phylogenetic relationships is tested by adding multiple congeneric or confamilial taxa that represent coding UCEs, UCEs from previous studies and UCEs obtained from genomes. We hypothesize that analyzing data as amino acids versus nucleotides can influence the inferred phylogenetic relationships. To test this, we reconstruct and compare phylogenies using nucleotide and amino acid data sets from sequences derived from both transcriptomes and ultraconserved regions of the genome. We found that nucleotide data sets converge on a similar topology—including the recovery of the symphytognathoid representatives as a clade—whereas amino acid data sets did not. This outcome suggests that reducing the number of characters included in nucleotide data sets via translation to amino acids is detrimental to the topological stability of phylogenetic inference.

## Results and Discussion

Statistics for all analyzed data sets are listed in supplementary table 1, Supplementary Material online. A few clarifications are provided here.

### CodingUCEs

With the current taxon sample, 2,019 loci were obtained (before occupancy filtering), out of which 1,931 UCEs were recovered from the transcriptomes analyzed in Fernández et al. (2018). This means that the transcriptomic analysis of Fernández et al. (2018) contained at least 1,931 coding UCE regions, out of the 2,021 possible UCEs targeted by the spider probe set of Kulkarni et al. (2020) (95.5%), making both data sets nearly identical in gene composition, and thus straightforward to combine. The number of UCEs recovered from individual transcriptomes (i.e., taxon-wise) ranged between 62 and 897 (*µ* = 436.18) (supplementary table 2, Supplementary Material online). Two taxa out of a total of six nonspider outgroup taxa, *Phrynus marginemaculatus* and *Limulus polyphemus*, yielded too few UCE loci, so they were omitted from the final data set.

### AllUCEs

This data set included a combination of the taxon sample of UCEs recovered from the transcriptomes (Fernández et al. 2018) and UCEs (Kulkarni et al. 2020). Three ingroup species (*Amaurobius ferox*, *Deinopis longipes*, and *Nesticus cooperi*) were removed from the *AllUCEs50* data set because they did not have any locus represented in the final alignment. This data set (*AllUCEs50*), with only 21 loci, resulted in a phylogeny in which many families were polyphyletic and thus, we have excluded this tree topology (see supplementary trees, Supplementary Material online) from our further analyses and discussion.

### Noncoding

Six terminals (*Bothriurus keyserlingi*, *Centruroides sculpturatus*, *Sofanapis antillanca*, *Euryopis* sp., *Nesticus gertschi*, and Chediminae sp.) were likewise removed from the phylogenetic analyses because they were represented by very few (<30) noncoding regions.

### Efficiency of the Spider Probes in Capturing *codingUCEs*

Out of 248 taxa in the *AllUCEs* data set, 40 genera had multiple representatives obtained from transcriptomes or UCEs. Although the UCE sequences were mapped to the spider probe set, their library preparations were enriched with either the same (Kulkarni et al. 2020) or the Arachnida probe set of Starrett et al. (2017) and Wood et al. (2018). All such genera were monophyletic, except *Segestria* (Segestriidae) and *Novanapis* (Anapidae), which were paraphyletic.

### Phylogenetic Relationships

**Fig. 1.**
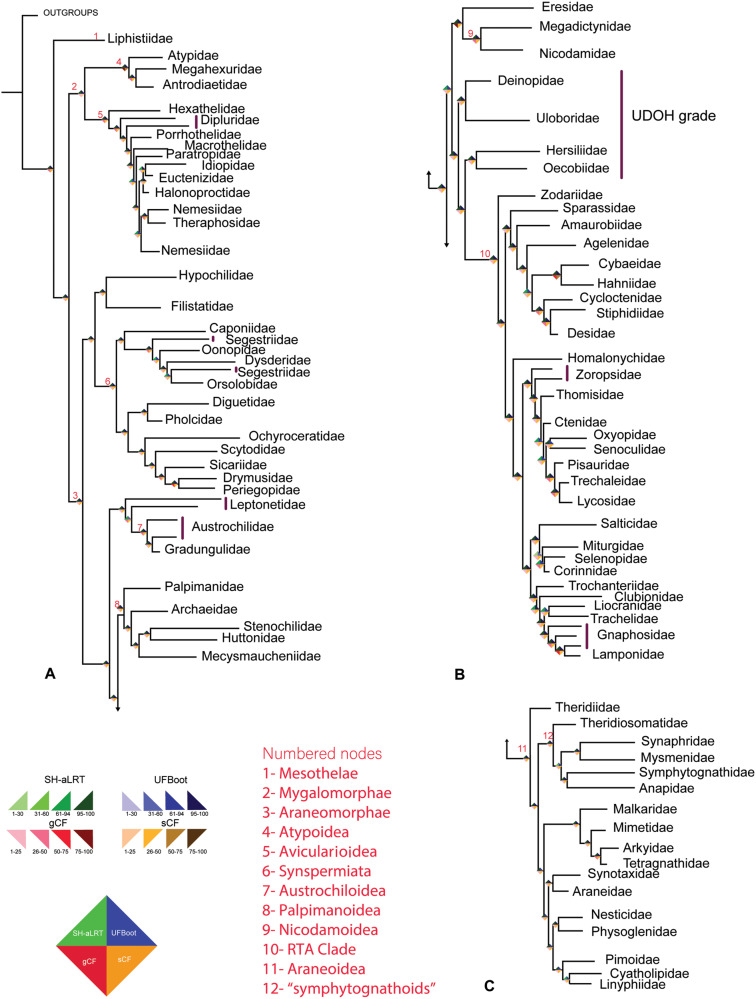
Maximum likelihood phylogeny of spiders resulting from the *AllUCEs10* data set (occupancy 10, 1,060 loci) collapsed to family level. Paraphyly is indicated by vertical violet bars. (*A*) All major lineages of spiders at family level except the RTA Clade and Araneoidea; (*B*) RTA Clade; (*C*) All 17 families of superfamily Araneoidea. The rhombi at the nodes indicate four support values: Shimodaira–Hasegawa-like approximate likelihood ratio test (left top), ultrafast bootstrap (right top), gene concordance factor (gCF) (left bottom), and site concordance factor (sCF) (right bottom). The numbers at the node indicate clades as described. Branch lengths are not to be scaled. For the original sampled tree, see supplementary figure 2, Supplementary Material online.

The *AllUCEs* data sets had the highest taxon representation of all data sets, including 88 out of 120 known spider families (World Spider Catalog 2020). Topology tests were conducted between different occupancies of the *AllUCEs* set. *AllUCEs25* was significantly rejected (supplementary table 3, Supplementary Material online) and thus, we base our discussion mainly on the *AllUCEs10* data set (fig. 1 and supplementary fig. 2, Supplementary Material online) and highlight relevant aspects of other topologies briefly below, except for “noncoding regions” which are discussed in a separate section. The nodal support values –SH-aLRT and ultrafast bootstrap (UFBoot) replicates are respectively mentioned in parentheses for each relationship. For gene and site concordance factors, refer to figure 1 and supplementary figure 2, Supplementary Material online.

**Fig. 2.**
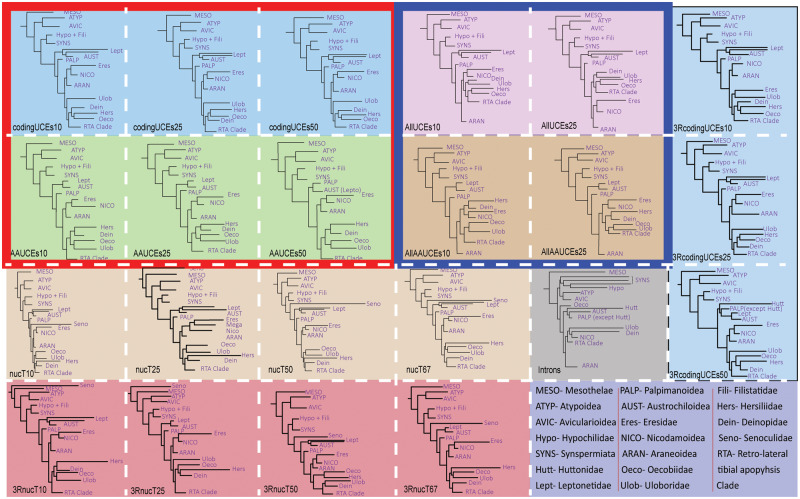
Maximum likelihood phylogenies of spiders resulting from different data sets at various occupancies. Each colored box indicates a data set corresponding to supplementary table 2, Supplementary Material online. The first and second rows represent phylogenies resulting from data analyzed as nucleotides and amino acids, respectively, of *codingUCEs* (outlined red) and *AllUCEs* (outlined blue).

All data sets (except noncoding) included a unanimously strong UFBoot support (>95%) for the major Araneae lineages such as Mesothelae, Opisthothelae, Mygalomorphae, and Araneomorphae (figs. 1 and 2; supplementary table 2, Supplementary Material online). Within Araneomorphae, conflicting relationships were recovered within the family Leptonetidae and the relationships among the UDOH families, and with Araneoidea and the RTA Clade (figs. 1 and 2; supplementary table 2, Supplementary Material online, see supplementary trees, Supplementary Material online). To briefly describe these conflicts, the UDOH families formed a clade with *AllAAUCE*s, but constituted a grade in the analyses of all other data sets. Araneoidea was recovered as the sister group to Nicodamoidea plus Eresidae in the analyses of all the data sets except *AllUCEs10* and its amino acid data sets (fig. 2). The placement of the long Senoculidae branch varied across analyses from nesting within the RTA Clade to a sister group to the Araneae branch. This recalcitrance may be indicative of a poor sequence quality.

### Phylogenomic Data as Amino Acids versus Nucleotides

**Fig. 3.**
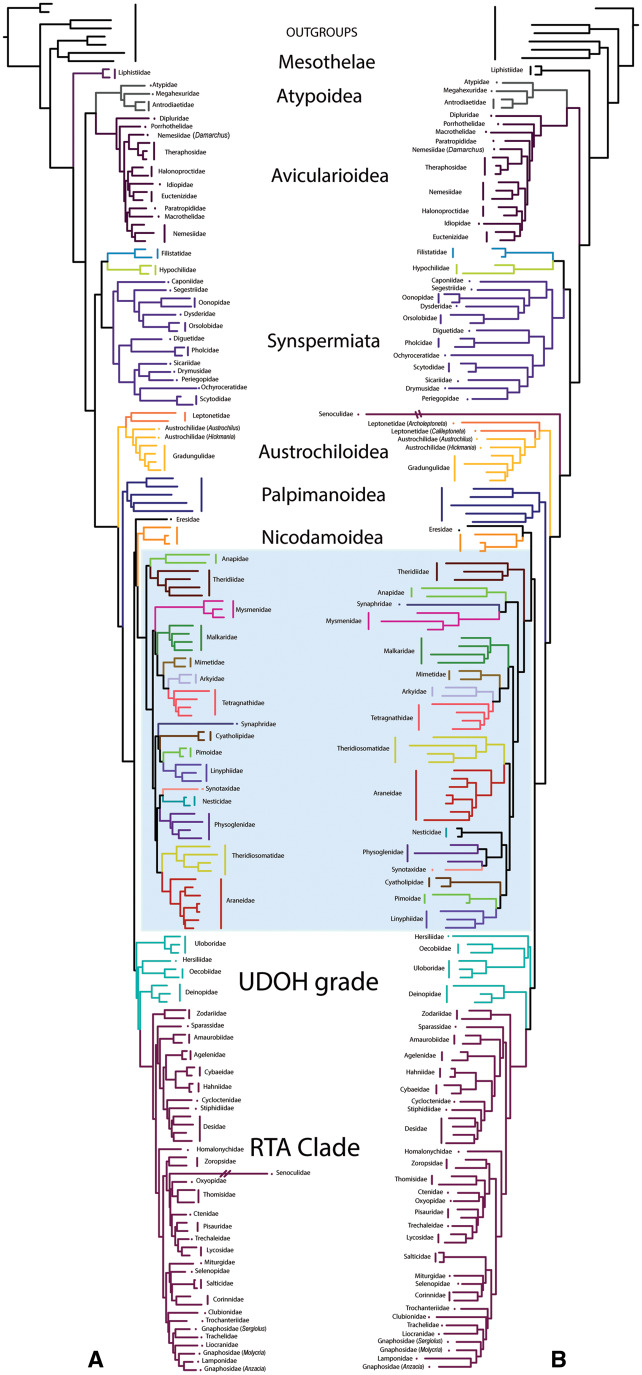
Comparison of phylogenetic relationships between (*A*) transcriptomic phylogeny as published by Fernández et al. (2018) using amino acids, and (*B*) *nucT* (Fernández et al. 2018, transcriptome data set analyzed as nucleotides). Both phylogenies were constructed using occupancy of 67%. The highlighted blue box indicates Araneoidea families.

Phylogenies resulting from the transcriptome data analyzed as amino acids (Fernández et al. 2018; fig. 3*A* of this study) and as nucleotide sequences (*nucT67* data set, fig. 3*B*) at an occupancy of 67% were congruent at many nodes. Notable differences were found among the UDOH families and in the internal arrangement of Araneoidea. Although Deinpoidae was sister group to the RTA Clade in both trees, Hersiliidae was either the sister group of Oecobiidae (amino acid data) or the sister group to Oecobiidae plus Uloboridae (nucleotide data; fig. 3). Within Araneoidea, Theridiidae plus Anapidae formed a clade sister group to all remaining araneoid families with amino acid data, however with nucleotides, Theridiiidae was the sister group of the clade that included all the remaining araneoid families. This latter placement is consistently recovered with all other data sets (see supplementary files, Supplementary Material online).

**Fig. 4.**
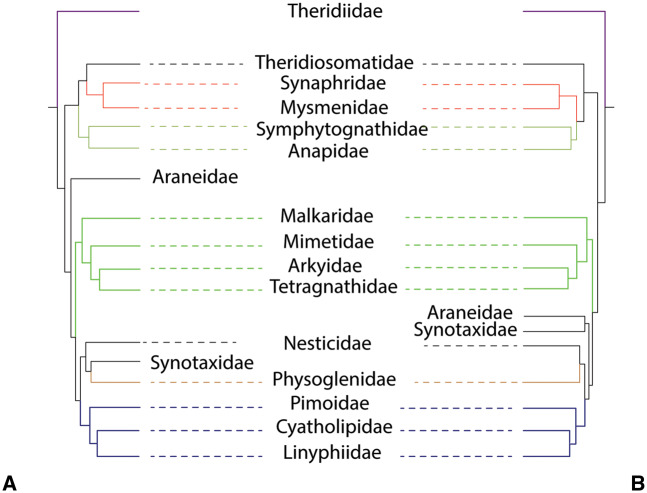
Comparison of interfamilial relationships of Araneoidea. (*A*) *AllAAUCEs* tree, (*B*) *AllUCEs* tree. Occupancy of both phylogenies was 10%. Colored branches indicate family relationships that are congruent in both trees.

In recently published phylogenomic analyses using amino acid data (Fernández et al. 2018; Michalik et al. 2019), Leptonetidae was recovered as monophyletic with all the amino acid data sets, that is the *AAUCE*, *AllAAUCE* and also in Fernández et al. (2018), but the family was paraphyletic with the nucleotide data sets (figs. 2 and 3;supplementary fig. 3, Supplementary Material online). This is notable given that *Archoleptoneta* species are cribellate whereas all other leptonetids, including other archoleptonetines (namely, *Darkoneta*), are ecribellate (Ledford and Griswold 2010). A recent UCE study (analyzed as nucleotides) using a dense sample of leptonetids also recovered diphyly with Archoleptonetinae separate from Leptonetinae (Ramírez et al. 2020).

The linyphioids (Linyphiidae and Pimoidae) were monophyletic with *nucT* data sets (>95% UFBoot), *codingUCEs* (>95% UFBoot), and *AAUCEs10* (<95% UFBoot), however other data sets obtained paraphyly of linyphioids, but the pertinent nodes were poorly supported. The monophyly of linyphioids has been supported with morphology (Hormiga 1994, 2008; Hormiga and Tu 2008), six standard Sanger markers (Arnedo et al. 2009; Dimitrov et al. 2017; Wheeler et al. 2017), and transcriptomes (Fernández et al. 2018).

Gnaphosidae was paraphyletic in both Fernández et al. (2018) (supplementary fig. 3*A*, Supplementary Material online) and the current study (figs. 1 and 3*B*; supplementary fig. 3*B*, Supplementary Material online). In the current study, Lamponidae nested within Gnaphosidae, whereas, in Fernández et al. (2018), Trachelidae, Liocranidae, and Lamponidae nested within Gnaphosidae. Optimized taxon sampling in this part of the tree would be required to stabilize these relationships.

### Removal of Third Codon Positions

Including third codon positions in phylogenetic analyses may influence inferred relationships due to saturation of synonymous nucleotide substitutions and rate heterogeneity, therefore explaining differences between analyzing data as amino acids and nucleotides, and thus, some authors recommend exclusion of saturated third codon positions (e.g., Breinholt and Kawahara 2013; O’Connor et al. 2014). In our study, the trees resulting from the analyses with (*codingUCEs* and *nucT* data sets) and without (*3RcodingUCEs* and *3RnucT* data sets) third codon positions were congruent at most nodes. The differences were as follows: the *3RcodingUCEs10* data set yielded Eresidae as the sister group of Uloboridae whereas in all the other data sets with the third codon positions removed, Eresidae was sister group to Nicodamoidea and the *3RcodingUCEs50* data set yielded a paraphyletic Palpimanoidea.

### Noncoding Regions

All spider families were monophyletic with good support (>95% UFBoot), however most interfamilial relationships and deeper nodes received poor support (see supplementary trees, Supplementary Material online). Many groups that were corroborated with all other data sets were recovered differently when noncoding regions were analyzed alone. For example, mygalomorphs were the sister group of a paraphyletic Synspermiata that included Hypochilidae, and the austrochiloids were nested within Palpimanoidea and polyphyletic UDOH families (fig. 2). These unusual relationships could be an artifact due to the overall small amount of data included in this data set; a similar pattern was also observed when analyzing high occupancy (>70%) coding region data sets (supplementary file, Supplementary Material online). The high variability in sizes of noncoding regions between distantly related taxa also requires an evaluation of the potential effect of alignment schemes on resulting relationships. Analyzing them together with exons, as in *AllUCEs*, could be a useful strategy since the conserved coding regions may alleviate the effects of alignment procedures. The use of appended exonic regions to align noncoding regions needs further exploration. HybPiper recovers nonexonic regions which may also include intergenic regions in addition to noncoding regions, which are difficult to parse.

### Monophyly of the Miniature Orb Weavers

The “symphytognathoids” were monophyletic in the trees resulting from the analyses of the *codingUCEs*, *AAUCEs*, *AllUCEs*, *AllAAUCEs*, and *nucT*, except *AAUCEs50* and *nucT67* which recovered Theridiosomatidae as sister group to Araneidae whereas the remaining “symphytognathoids” formed a clade. In the *AllUCEs* tree (fig. 4), this clade included the families Anapidae, Mysmenidae, Symphytognathidae, Synaphridae, and Theridiosomatidae (100/100 UFBoot/SH-aLRT for the whole clade), whereas the *codingUCEs* included all these families except Symphytognathidae (not sampled). The family Synaphridae was sister group to Mysmenidae in *AllUCEs* (100/100%), whereas it was sister group to Anapidae in *codingUCEs* phylogenies. Only 2.29% of loci (∼24 loci) and 29.5% of sites (∼68,655 sites) support the monophyly of “symphytognathoids” in the *AllUCEs10* data set (fig. 1), meaning that the remaining sites and loci support alternative relationships in lower fractions. In the trees resulting from the analyses of the other data sets, *AllUCEs*, *AllAAUCEs*, *codingUCEs*, and *nucT*, Theridiosomatidae was the sister group of the remaining “symphytognathoids” with two exceptions of high occupancies, as mentioned above (*AAUCEs50* and *nucT67*). The *AllAAUCEs* recovered Theridiosomatidae as sister group to Synaphridae plus Mysmenidae and this clade was sister group to Symphytognathidae plus Anapidae (fig. 4, see supplementary files, Supplementary Material online). The removal of third codon positions from the transcriptomes analyzed as nucleotides (*3RnucT* data sets) supported “symphytognathoid” monophyly at occupancies of 10%, 25%, and 50%, whereas at 67% occupancy, Theridiosomatidae was the sister group of Araneidae and the other “symphytognathoid” families formed a clade. The removal of third codon positions from UCEs derived from transcriptomes (*3RcodingUCEs* data sets) rendered the “symphytognathoid” families polyphyletic (table 2 and supplementary trees, Supplementary Material online).

The inclusion of Synaphridae within “symphytognathoids” had been suggested before (Lopardo and Hormiga 2008; Lopardo et al. 2011), although these studies were cautious about such placement due to the absence of Cyatholipidae representatives in their analyses. Fernández et al. (2018) found Synaphridae to be the sister group of the linyphioid clade. Because Kulkarni et al. (2020) did not include any synaphrid, its position using strictly UCE data could not be tested. We included a synaphrid exemplar, *Cepheia longiseta* (from Fernández et al. 2018), and our results corroborate the placement of Synaphridae within the “symphytognathoid” clade.

The monophyly of “symphytognathoids” is supported by several morphological synapomorphies (Lopardo et al. 2011). Although morphology and UCEs support the monophyly of “symphytognathoids,” six-gene Sanger-based data and sequences from transcriptomes analyzed as amino acids do not support “symphytognathoid” monophyly (Lopardo et al. 2011; . Dimitrov et al. 2012, 2017; Wheeler et al. 2017; Fernández et al. 2018; Kallal et al. forthcoming). Unstable and conflicting “symphytognathoid” familial relationships hinder addressing questions about the evolution of their unique diversity of web architectures, transformations in female pedipalps (reduction and loss), and transformations of their respiratory systems. For example, although referred to as miniature “orb weavers,” anapid web architecture is quite variable as they are known to build typical orb webs and their modifications, sheet webs or, theridiid-like cobwebs. Most mysmenids build spherical or planar orbs, symphytognathids build a 2D horizontal orb web, at least some synaphrids build sheet or irregular webs, and theridiosomatids build orb webs, some of them highly modified (e.g., sticky lines connected to water surface) (Coddington and Valerio 1980; Eberhard 1987; Rix and Harvey 2010; Lopardo et al. 2011). In each of these “symphytognathoid” families (except Synaphridae), there is at least one genus with a kleptoparasitic lifestyle accompanied by loss of the foraging web in all its constituent species. Adult anapid females have either reduced segments in the pedipalp, a knob-like protuberance, or have lost the palp entirely, like their putative sister family Symphytognathidae. Female pedipalps in the remaining “symphytognathoid” families bear all the segments, like all other spiders.

Our results and those from Kulkarni et al. (2020) indicate that “symphytognathoids” are monophyletic when analyzed as nucleotide data and when about a hundred or more loci are available. There is also a clear tradeoff between occupancy and phylogenetic signal. Low-occupancy data matrices contain more missing data than high occupancy data sets, and missing data can influence the outcome of phylogenetic analyses, both topologically and in branch lengths (Lemmon et al. 2009). In the case of “symphytognathoids,” a high occupancy data set of 70% with 433 loci (“500Spid_70” data set of Kulkarni et al. [2020]) also supported “symphytognathoid” monophyly, suggesting that miniature orb-weaving spiders are indeed a lineage.

### Unstable Nodes in the Spider Tree of Life

The phylogenetic relationships of the UDOH group of families relative to the RTA Clade and the interfamilial relationships of Araneoidea vary across analytical conditions, depending on the type (coding or coding plus noncoding) and amounts of data. For example, in the case of Araneoidea, coding data (*codingUCE*, *AAUCE*, *nucT*) exclusively recover this clade as sister group to Nicodamoidea plus Eresidae. However, when combined with nonexonic data, Araneoidea is sister group to a clade consisting of Nicodamoidea plus Eresidae, the RTA Clade, and the UDOH families–with the exception of the *AllUCEs25* data set. The UDOH grade consists of Uloboridae, Deinopidae, Oecobiidae, and Hersiliidae, of which the first two families are the only cribellate orb-weaving groups, whereas all remaining orb-weaving spider families are ecribellate and placed within Araneoidea. On the other hand, exploration of molecular data across a variety of analytical treatments has shown that many nodes in the spider tree of life are stable across different occupancies. For example, the sister group relationship of Nicodamoidea and Eresidae, the Hypochilidae plus Filistatidae clade, the monophyly of Synspermiata, and the “symphytognathoid” clade are all robust hypotheses.

### Nodal Support Values

Overall, we found that the gene concordance and site concordance factor values were correlated (supplementary fig. 1*a* and *c*, Supplementary Material online). The UFBoot was 100% for most nodes and the SH-aLRT was mostly >85% (fig. 1 and supplementary fig. 2, Supplementary Material online). Both concordance factors were >50% for congeneric taxa (fig. 1), meaning that >50% of the sites and loci support the monophyly of those genera. Gene and site concordance values ranged between 1% and 95%. These values were generally >50% for congeneric taxa and were lower between families and deeper nodes (fig. 1). Several alternative placements, including that of leptonetids, nicodamoids with respect to Araneoidea and the UDOH families, had high UFBoot within our trees (see supplementary files, Supplementary Material online) and also compared with the trees of Fernández et al. (2018).

### Occupancy and Missing Data

Our results show that high occupancy data sets may yield unstable relationships due to the small number of genes often represented in such data sets (fig. 2 and supplementary table 2 and supplementary trees, Supplementary Material online). A similar phenomenon of unusual relationships at high occupancies was observed in phylogenetic analyses of spider transcriptomes (Kallal et al. forthcoming). Low-occupancy data sets contain larger amounts of data but also contain larger amounts of missing data. An increase in the proportion of missing data is known to increase the risk of systematic error (Roure et al. 2013). However, recent empirical studies with genome-scale data have shown that excluding genes with high amounts of missing data may weaken the resolution and consistency of the resulting tree (Prasanna et al. 2020). Chan et al. (2020) found that different data classes such as UCEs, exons, and introns contain different phylogenetic signal; however, an unfiltered combination (low occupancy) of such data converged on a similar topology. One study suggests that if by allowing more missing data, taxon, and gene sampling can be improved, the lower occupancy matrices should be preferred (Streicher et al. 2016). In addition, allowing missing data may allow to detect gene gains/losses specific to certain lineages. Such information may be lost in high occupancy data sets due to the exclusion of genes present in some clade versus sequencing failures. CAT + Γ models may alleviate systematic error (Roure et al. 2013) but this was not tested in the present study. Evaluation of model adequacy (Ripplinger and Sullivan 2010; Duchêne et al. 2018) may be a potential next step to further improve the phylogenetic inference of the evolutionary history of spiders, but our goal here was to evaluate for the first time the use of amino acids versus DNA.

## Conclusions

We have used spiders (Araneae) as a study system to address incongruence among different classes of genomic data in phylogenetic analyses. We scrutinized sequence data from different sources (i.e., mRNA and DNA) and analyzed the protein-coding regions either as amino acids or as nucleotides, with and without third codon positions; we also analyzed noncoding regions. All data sets, except the noncoding data, converged upon a similar pattern of phylogenetic relationships, which was also similar to the trees derived from low-occupancy matrices resulting from the analysis of UCEs from genomic data (Kulkarni et al. 2020). It is clear that lower amounts of data either due to amino acid translation, increasing matrix occupancy, or both, can cause topological conflicts at some nodes in the spider tree of life and with the sequencing strategies employed here. Although a threshold cannot be established as to how much data are optimal to resolve such topological conflicts, at least 500 loci seem necessary, based on our results. Our results suggest that using nucleotide data and/or low occupancies to analyze thousands of loci may prove to be a better strategy for studying higher level phylogenetic relationships than using amino acids and high occupancies which would yield a much smaller data set.

Conflicting results are more difficult to interpret when mutually exclusive alternative relationships are highly supported, particularly when using bootstrapping as a measure of support on large data sets. Hence, alternative branch support measures that are computationally tractable for genome-scale data sets, like concordance factors, need to be further explored.

In the interest of spider systematics, we demonstrate that phylogenetic incongruences can be reduced by analyzing genome-scale nucleotide data sets, especially at low occupancies. Some of the contentious hypotheses, such as the phylogeny of “symphytognathoids,” were impacted by the data class, composition, and taxon sampling used. We recovered a congruent support for their monophyly across a range of low-occupancy data sets. This robustly supported hypothesis on the phylogenetic relationships of the miniature orb-weaving families will provide an opportunity to unravel the evolutionary history of foraging webs.

## Materials and Methods

### Taxon Sampling

The ultraconserved sequences (UCEs) for this study were obtained from a series of studies focusing on arachnids, including Starrett et al. (2017), Wood et al. (2018), and Kulkarni et al. (2020). Transcriptomes were obtained from Bond et al. (2014), Fernández et al. (2014, 2018), Garrison et al. (2016), Sharma et al. (2014), and Zhao et al. (2014). Ultraconserved loci were also retrieved from publicly available spider genomes of *Latrodectus hesperus* (Theridiidae; i5K Consortium 2013), *Loxosceles reclusa* (Sicariidae; i5K Consortium 2013), *Trichonephila clavipes* (Araneidae; Babb et al. 2017), *Parasteatoda tepidariorum* (Theridiidae; Schwager et al. 2017), and *Stegodyphus mimosarum* (Eresidae; Sanggaard et al. 2014). Outgroups include the horseshoe crab *L. polyphemus* and *Tachypleus tridentatus* (Xiphosura); the scorpions *B. keyserlingi*, *C. sculpturatus*, *Chaerilus celebensis*, and *Pandinus imperator* (Scorpiones); the whip-spiders *Damon variegatus*, *Damon* sp., and *P. marginemaculatus* (Amblypygi); the vinegaroon *Mastigoproctus giganteus* (Uropygi) and the short-tailed whip-scorpion *Stenochrus portoricensis* (Schizomida). The analysis was rooted using Xiphosura since it is the only member outside Arachnopulmonata, irrespective of whether we follow the traditional hypothesis of Xiphosura being an outgroup to Arachnida (e.g., Lozano-Fernández et al. 2019), or the alternative hypothesis placing them within Arachnida (see Ballesteros and Sharma 2019).

### Transcriptome Assembly

Raw sequences were corrected for read errors using Rcorrector (Song and Florea 2015). Low-quality reads and adapters were trimmed with Trim Galore! 0.2.6 (http://www.bioinformatics.babraham.ac.uk/projects/trim_galore, last accessed January 10, 2020) by setting the quality parameter to 30 and a phred cut-off to 33; reads shorter than 25 bp were discarded. Ribosomal RNA was filtered using the default settings in Bowtie 2.9.9 (Langmead and Salzberg 2012). De novo strand-specific assemblies were generated using Trinity 2.0.6 (Grabherr et al. 2011; Haas et al. 2013) with a path reinforcement set to 75. Redundancy reduction was done using CD-HIT-EST (Fu et al. 2012) with 95% global similarity. Assemblies were completed using the Colonial One High Performance Computing Cluster at The George Washington University and the Smithsonian Institution High Performance Cluster at the Smithsonian Institution. Unlike in previous phylotranscriptomic analyses of spiders (Bond et al. 2014; Fernández et al. 2014, 2018; Sharma et al. 2014; Zhao et al. 2014; Garrison et al. 2016), the final DNA sequences were not translated to amino acids.

### Recovering UCEs from Transcriptomes

The FASTA files of transcriptomes resulting from CD-HIT-EST were converted to 2-bit format using faToTwoBit, (Kent 2002). Then, in the PHYLUCE environment (publicly available at https://phyluce.readthedocs.io/en/latest/tutorial-three.html), we created a temporary relational database to summarize probe to assembly match using: *phyluce_probe_run_multiple_lastzs_sqlite* function on the 2-bit files.The ultraconserved loci were recovered by the *phyluce_probe_slice_sequence_from_genomes* command. The resulting FASTA files were treated as contigs and used to match the reads to the Spider2Kv1 probes.

### Analyzing UCEs as Amino Acids

The nucleotide reads from UCE and transcriptome contigs were assembled, aligned, trimmed, and processed to obtain selected loci with taxon occupancies of 10%, 25%, and 50% using PHYLUCE. All locus files in nexus format were converted to fasta form and translated to amino acids using seqmagick (https://seqmagick.readthedocs.io/en/latest/). These translated UCE loci were concatenated using HybPiper (Johnson et al. 2016).

### Analyzing Transcriptomes as Nucleotides

The FASTA files of transcriptomes resulting from CD-HIT-EST were translated to amino acids using Transdecoder (Haas et al. 2013). Orthologs were recovered from the peptide reads using BUSCO (Simão et al. 2015). Nucleotide data with ortholog indices and gene files were obtained using NOrthGen (https://github.com/sskspider/NOrthGen; supplementary fig. 4, Supplementary Material online). Gene files were aligned using MAFFT v7 (Katoh and Standley 2013) and trimmed using trimAl v1.2 (Capella-Gutiérrez et al. 2009). All orthologs were concatenated using the HybPiper (Johnson et al. 2016). Third codon positions were removed using rmThirdCodon (https://github.com/iamciera/rmThirdCodon).

### Obtaining Noncoding Regions

Noncoding regions were extracted from the raw UCE sequence files obtained from Starrett et al. (2017), Wood et al. (2018), and Kulkarni et al. (2020). A target file database of exons was compiled using UCEs extracted from the transcriptomes of *D. variegatus*, *Lo. deserta*, Nicodamidae sp., *T. clavipes*, *Hebestatis theveneti*, *Palpimanus gibbulus*, *Kukulcania hibernalis*, *S. mimosarum*, *Liphistius malayanus*, *Anahita punctulata*, and *Megahexura fulva* from Fernández et al. (2018) and the genome of *Par. tepidariorum* (Schwager et al. 2017). These taxa were chosen to represent Araneae-wide samples and their closest relatives used as outgroups. HybPiper (Johnson et al. 2016) was run on the raw UCE sequence files and matched against the target file. After exon matching was completed, we used the *retriever* pipeline to extract the noncoding sequences from the raw UCE sequences. Small sequences <50 bp (taken as an arbitrary threshold) were deleted and the remaining noncoding sequences were aligned using MAFFT v7 (Katoh and Standley 2013) and concatenated using HybPiper (Johnson et al. 2016).

### Phylogenomic Analyses

**Fig. 5.**
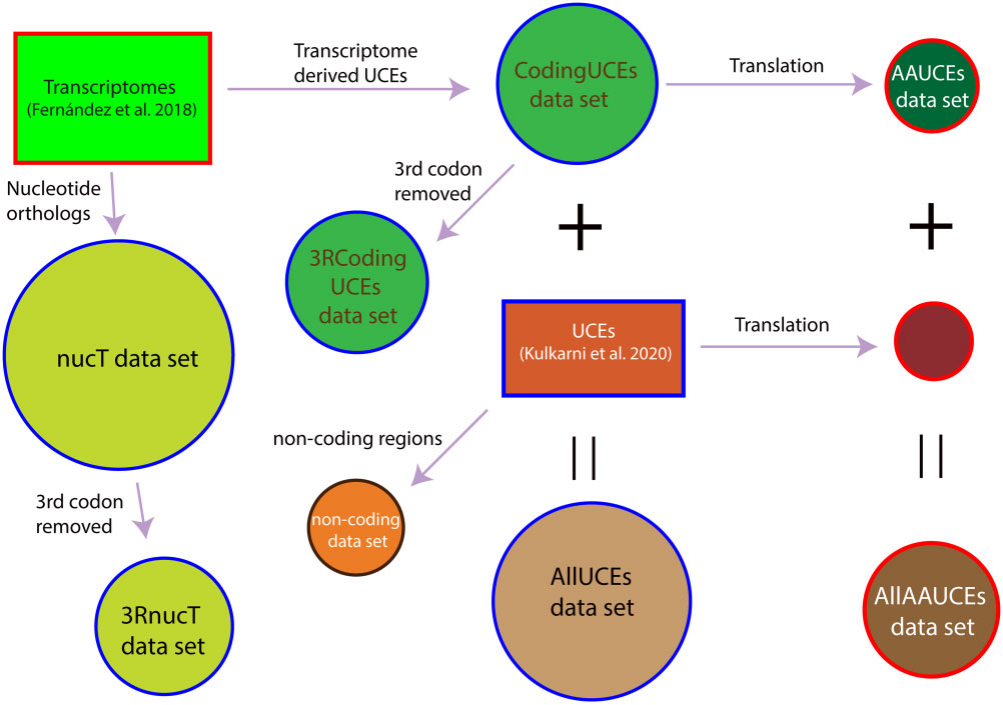
Schematic representation of data classes analyzed in this study in a maximum likelihood framework. Squares indicate original data sets from Fernández et al. (2018) and Kulkarni et al. (2020), and circles indicate matrices analyzed in our study. Circles with red outline indicate amino acid data set, black outline indicates noncoding region data set, and the circles with outline indicate nucleotide data sets. UCE, ultraconserved elements.

The ultraconserved loci recovered from the transcriptomes are referred to as *codingUCEs* in the following text. We built eight data sets (supplementary table 2, Supplementary Material online), as follows. All data sets (fig. 5) were analyzed at different occupancies, for a total of 15 different analyses (supplementary table 2, Supplementary Material online):

1. *codingUCEs* data set: The UCEs recovered from transcriptomes and analyzed as nucleotide sequences with all codon positions at occupancies of 10%, 25%, and 50%. This data set contains only exons that are ultraconserved.
2. *AAUCEs* data set: Sequences from *codingUCEs*, above, were translated to amino acids and analyzed at occupancies of 10%, 25%, and 50%.
3. *AllUCEs* data set: The *codingUCEs* data set was combined with the UCEs from taxa included in Kulkarni et al. (2020) analyzed at occupancies of 10%, 25%, and 50%. This data set of UCEs contains both exons as well as noncoding regions.
4. *AllAAUCEs* data set: The amino acid sequences for the taxon sampling similar to *AllUCEs* data sets analyzed at occupancies of 10%, 25%, and 50%. This data set contains only exons that are ultraconserved.
5. *nucT* data set: Transcriptomes analyzed as nucleotides with all codon positions at occupancies of 10%, 25%, and 50% and 67%. This data set contains only exons that may or may not be ultraconserved.
6. *noncoding regions* data set: Noncoding regions obtained from the UCE data set of Kulkarni et al. (2020).
7. *3RcodingUCEs* data set: Third codon removed from the *codingUCEs* data set.
8. *3RnucT* data set: Third codon removed from the *nucT* data set.

Contigs from all DNA sequences were matched to the Spider2Kv1 probe set (Kulkarni et al. 2020) at minimum coverage and minimum identity of 65 each. Phylogenetic analyses were performed on the unpartitioned, concatenation of loci using IQ-TREE v.1.6.9 (Nguyen et al. 2015). Model selection was allowed for each data set using the TEST function of ModelFinder in IQ-TREE (Kalyaanamoorthy et al. 2017; Hoang et al. 2018).

Nodal support was estimated via 1,000 UFBoot replicates (Hoang et al. 2018) and Shimodaira–Hasegawa-like approximate likelihood ratio test (SH-aLRT) (Guindon et al. 2010). To reduce the risk of overestimating branch support with UFBoot due to model violations, we appended the command -bnni. With this command, the UFBoot optimizes each bootstrap tree using a hill-climbing nearest-neighbor interchange search based on the corresponding bootstrap alignment (Hoang et al. 2018). We used concordance factors, a metric focusing on whether the best tree represents the signal well, as implemented in IQ-TREE v1.7-betaX (Minh et al. 2020). Gene concordance factor (gCF) indicates the percentage of gene trees containing a given branch in the maximum likelihood tree and site concordance factor (sCF) indicates the percentage of decisive alignment sites supporting a branch (Minh et al. 2020) and it provides insights into incomplete lineage sorting which may be a cause for discordance between the sites and the resulting trees (Zhang et al. 2019). We mapped the gCF against sCF with respect to UFBoot and the SH-aLRT using R version 3.6.0 (R Core Team 2019).

We chose our preferred tree to guide the discussion of the results by conducting topology tests, namely, approximately unbiased (AU), bootstrap proportion, SH-aLRT, Kishino–Hasegawa, and expected likelihood weight using 10,000 resampling-estimated log-likelihoods in IQ-TREE among the *AllUCEs* data set.

## Supplementary Material

Supplementary data are available at *Molecular Biology and Evolution* online.

## Supplementary Material

msaa251_Supplementary_Data
