# Supplementary material for: Interrogating Genomic-Scale Data to Resolve Recalcitrant Nodes in the Spider Tree of Life
Source: Mol Biol Evol. 2020 Sep 28;38(3):891–903. doi: 10.1093/molbev/msaa251 (PMC7947752; doi:10.1093/molbev/msaa251)
Supplement: msaa251_Supplementary_Data [file msaa251_supplementary_data.pdf]

Supplementary Figure 1: Site concordance factors mapped against gene concordance factors with respect to ultrafast bootstrap (A,C) and Shimodaira-Hasegawa-like approximate likelihood ratio test (B,D). Figures A and B indicate plots for the *AllUCEs10* data set, and C and D indicate plots for the *AllUCEs25* data set.

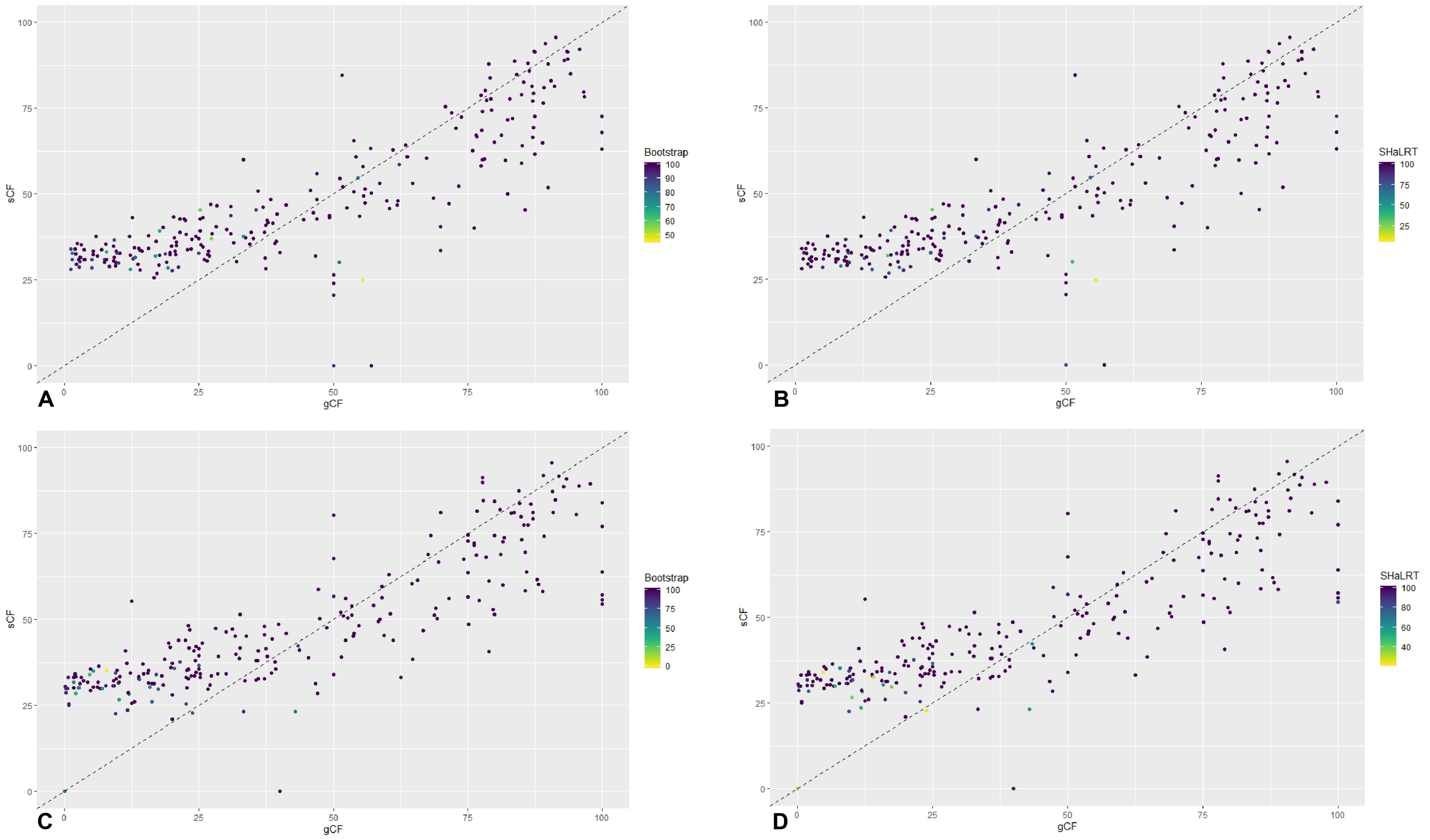

A
B
C
D
sCF
gCF
Bootstrap
SHaLRT
0
25
50
75
100

Supplementary Figure 2: Maximum likelihood phylogeny of spiders resulting from the *AllUCEs10* data set (occupancy 10, 1,060 loci). A phylogeny with 248 taxa with taxon names in black indicating taxa from UCE studies, violet indicating UCEs recovered from Fernandez et al. (2018) and red indicating UCEs recovered from genomes. The rhombi at the nodes indicate four support values: Shimodaira-Hasegawa-like approximate likelihood ratio test (left top), ultrafast bootstrap (right top), gene concordance factor (gCF) (left bottom) and site concordance factor (sCF) (right bottom). The numbers at nodes indicate clades as described.

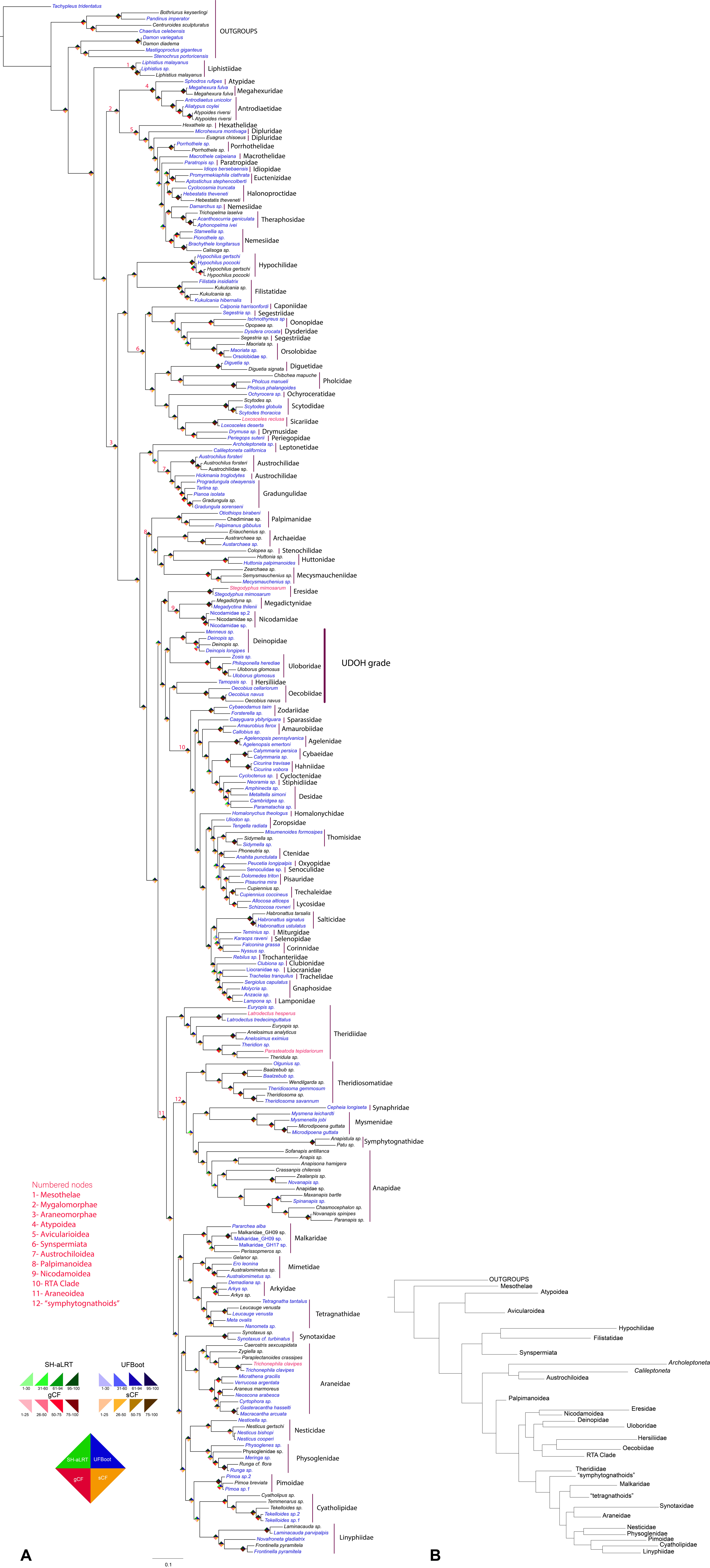

Tachypleus tridentatus
Bothriurus keyserlingi
Pandinus imperator
Centruroides sculpturatus
Chaerilus celebensis
Damon variegatus
Damon diadema
Mastigoproctus giganteus
Stenochrus portoricensis
OUTGROUPS
Liphistius malayanus
Liphistius sp.
Liphistius malayanus
Liphistiidae
Sphodros rufipes
Atypidae
Megahexura fulva
Megahexura fulva
Megahexuridae
Antrodiaetus unicolor
Aliatypus coylei
Atypoides riversi
Atypoides riversi
Antrodiaetidae
Hexathele sp.
Hexathelidae
Microhexura montivaga
Dipluridae
Euagrus chisoseus
Dipluridae
Porrhothele sp.
Porrhothele sp.
Porrhothelidae
Macrothele calpeiana
Macrothelidae
Paratropis sp.
Paratropidae
Idiops bersebaensis
Idiopidae
Promyrmekiaphila clathrata
Aptostichus stephencolberti
Euctenizidae
Cyclocosmia truncata
Hebestatis theveneti
Hebestatis theveneti
Halonoproctidae
Damarchus sp.
Nemesiidae
Trichopelma laselva
Acanthoscurria geniculata
Aphonopelma iviei
Theraphosidae
Stanwellia sp.
Pionothele sp.
Brachythele longitarsus
Calisoga sp.
Nemesiidae
Hypochilus gertschi
Hypochilus pococki
Hypochilus gertschi
Hypochilus pococki
Hypochilidae
Filistata insidiatrix
Kukulcania sp.
Kukulcania sp.
Kukulcania hibernalis
Filistatidae
Calponia harrisonfordi
Caponiidae
Segestria sp.
Segestriidae
Ischnothyreus sp.
Opopaea sp.
Oonopidae
Dysdera crocata
Dysderidae
Segestria sp.
Segestriidae
Maoriata sp.
Maoriata sp.
Orsolobidae sp.
Orsolobidae
Diguetia sp.
Diguetia signata
Diguetidae
Chibchea mapuche
Pholcus manueli
Pholcus phalangoides
Pholcidae
Ochyrocera sp.
Ochyroceratidae
Scytodes sp.
Scytodes globula
Scytodes thoracica
Scytodidae
Loxosceles reclusa
Loxosceles deserta
Sicariidae
Drymusa sp.
Drymusidae
Periegops suteri
Periegopidae
Archoleptoneta sp.
Calileptoneta californica
Leptonetidae
Austrochilus forsteri
Austrochilus forsteri
Austrochilidae sp.
Austrochilidae
Hickmania troglodytes
Austrochilidae
Progradungula otwayensis
Tarlina sp.
Pianoa isolata
Gradungula sp.
Gradungula sorenseni
Gradungulidae
Otiothops birabeni
Chediminae sp.
Palpimanus gibbulus
Palpimanidae
Eriauchenius sp.
Austrarchaea sp.
Austrarchaea sp.
Archaeidae
Colopea sp.
Stenochilidae
Huttonia sp.
Huttonia palpimanoides
Huttonidae
Zearchaea sp.
Semysmauchenius sp.
Mecysmauchenius sp.
Mecysmaucheniidae
Stegodyphus mimosarum
Stegodyphus mimosarum
Eresidae
Megadictyna sp.
Megadictyna thilenii
Megadictynidae
Nicodamidae sp.2
Nicodamidae sp.
Nicodamidae sp.
Nicodamidae
Menneus sp.
Deinopis sp.
Deinopis sp.
Deinopis longipes
Deinopidae
Zosis sp.
Philoponella herediae
Uloborus glomosus
Uloborus glomosus
Uloboridae
UDOH grade
Tamopsis sp.
Hersiliidae
Oecobius cellariorum
Oecobius navus
Oecobius navus
Oecobiidae
Cybaeodamus taim
Forsterella sp.
Zodariidae
Caayguara ybityriguara
Sparassidae
Amaurobius ferox
Callobius sp.
Amaurobiidae
Agelenopsis pennsylvanica
Agelenopsis emertoni
Agelenidae
Calymmaria persica
Calymmaria sp.
Cybaeidae
Cicurina travisae
Cicurina vobora
Hahniidae
Cycloctenus sp.
Cycloctenidae
Neoramia sp.
Stiphidiidae
Amphinecta sp.
Metaltella simoni
Cambridgea sp.
Paramatachia sp.
Desidae
Homalonychus theologus
Homalonychidae
Uliodon sp.
Tengella radiata
Zoropsidae
Misumenoides formosipes
Sidymella sp.
Sidymella sp.
Thomisidae
Phoneutria sp.
Anahita punctulata
Ctenidae
Peucetia longipalpis
Oxyopidae
Senoculidae sp.
Senoculidae
Dolomedes triton
Pisaurina mira
Pisauridae
Cupiennius sp.
Cupiennius coccineus
Trechaleidae
Allocosa alticeps
Schizocosa rovneri
Lycosidae
Habronattus tarsalis
Habronattus signatus
Habronattus ustulatus
Salticidae
Teminius sp.
Miturgidae
Karaops raveni
Selenopidae
Falconina grassa
Nyssus sp.
Corinnidae
Rebilus sp.
Trochanteriidae
Clubiona sp.
Clubionidae
Liocranidae sp.
Liocranidae
Trachelas tranquillus
Trachelidae
Sergiolus capulatus
Molycria sp.
Anzacia sp.
Gnaphosidae
Lampona sp.
Lamponidae
Euryopis sp.
Latrodectus hesperus
Latrodectus tredecimguttatus
Euryopis sp.
Anelosimus analyticus
Anelosimus eximius
Theridion sp.
Parasteatoda tepidariorum
Theridula sp.
Theridiidae
Olgunius sp.
Baalzebub sp.
Baalzebub sp.
Wendilgarda sp.
Theridiosoma gemmosum
Theridiosoma sp.
Theridiosoma savannum
Theridiosomatidae
Cepheia longiseta
Synaphridae
Mysmena leichhardti
Mysmenella jobi
Microdipoena guttata
Microdipoena guttata
Mysmenidae
Anapistula sp.
Patu sp.
Symphytognathidae
Sofanapis antillanca
Anapis sp.
Anapisona hamigera
Crassanapis chilensis
Zealanapis sp.
Novanapis sp.
Anapidae sp.
Maxanapis bartle
Spinanapis sp.
Chasmocephalon sp.
Novanapis spinipes
Paranapis sp.
Anapidae
Pararchaea alba
Malkaridae_GH09 sp.
Malkaridae_GH09 sp.
Malkaridae_GH17 sp.
Perissopmeros sp.
Malkaridae
Gelanor sp.
Ero leonina
Australomimetus sp.
Australomimetus sp.
Mimetidae
Demadiana sp.
Arkys sp.
Arkys sp.
Arkyidae
Tetragnatha tantalus
Leucauge venusta
Leucauge venusta
Meta ovalis
Nanometa sp.
Tetragnathidae
Synotaxus sp.
Synotaxus cf. turbinatus
Synotaxidae
Caerostris sexcuspidata
Zygiella sp.
Paraplectanoides crassipes
Trichonephila clavipes
Trichonephila clavipes
Micrathena gracilis
Verrucosa arenata
Araneus marmoreus
Neoscona arabesca
Cyrtophora sp.
Gasteracantha hasselti
Macracantha arcuata
Araneidae
Nesticella sp.
Nesticus gertschi
Nesticus bishopi
Nesticus cooperi
Nesticidae
Physoglenes sp.
Physoglenidae sp.
Meringa sp.
Runga cf. flora
Runga sp.
Physoglenidae
Pimoa sp.2
Pimoa breviata
Pimoa sp.1
Pimoidae
Cyatholipus sp.
Temmenarus sp.
Tekelloides sp.
Tekelloides sp.2
Tekelloides sp.1
Cyatholipidae
Laminacauda sp.
Laminacauda parvipalpis
Novafroneta gladiatrix
Frontinella pyramitela
Frontinella pyramitela
Linyphiidae
Numbered nodes
1- Mesothelae
2- Mygalomorphae
3- Araneomorphae
4- Atypoidea
5- Avicularioidea
6- Synspermiata
7- Austrochiloidea
8- Palpimanoidea
9- Nicodamoidea
10- RTA Clade
11- Araneoidea
12- "symphytognathoids"
SH-aLRT
UFBoot
1-30 31-60 61-94 95-100
1-30 31-60 61-94 95-100
gCF
sCF
1-25 26-50 50-75 75-100
1-25 26-50 50-75 75-100
SH-aLRT
UFBoot
gCF
sCF
A
0.1
OUTGROUPS
Mesothelae
Atypoidea
Avicularoidea
Hypochilidae
Filistatidae
Synspermiata
Archoleptoneta
Calileptoneta
Austrochiloidea
Palpimanoidea
Eresidae
Nicodamoidea
Deinopidae
Uloboridae
Hersiliidae
Oecobiidae
RTA Clade
Theridiidae
"symphytognathoids"
Malkaridae
"tetragnathoids"
Synotaxidae
Araneidae
Nesticidae
Physoglenidae
Pimoidae
Cyatholipidae
Linyphiidae
B

Supplementary Figure 3: Comparison of phylogenetic relationships between A. transcriptomes as published by Fernández et al. (2018), and B. *codingUCEs* (UCEs retrieved from the Fernández et al. (2018) transcriptome data set, with occupancy of 10) analyzed as DNA. The highlighted blue box indicates Araneoidea families. Note that the miniature orb weaving spider families (“symphytognathoids”)- Anapidae, Mysmenidae, Synaphridae and Theridiosomatidae (Symphytognathidae not sampled), are polyphyletic in the left tree, but monophyletic in the right tree.

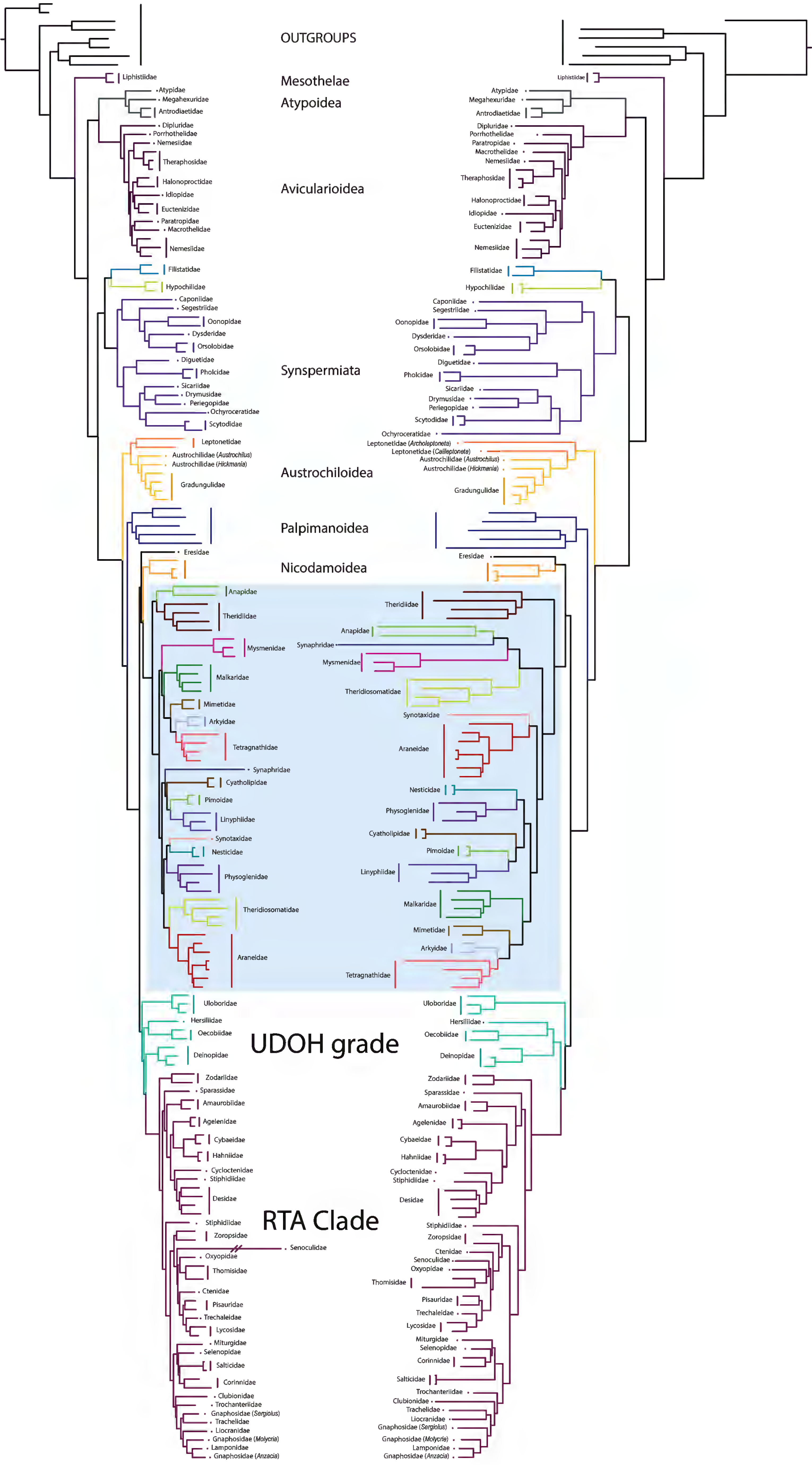

OUTGROUPS
Mesothelae
Atypoidea
Avicularioidea
Synspermiata
Austrochiloidea
Palpimanoidea
Nicodamoidea
UDOH grade
RTA Clade
Liphistiidae
Atypidae
Megahexuridae
Antrodiaetidae
Dipluridae
Porrhothelidae
Nemesiidae
Theraphosidae
Halonoproctidae
Idiopidae
Euctenizidae
Paratropidae
Macrothelidae
Nemesiidae
Filistatidae
Hypochilidae
Caponiidae
Segestriidae
Oonopidae
Dysderidae
Orsolobidae
Diguetidae
Pholcidae
Sicariidae
Drymusidae
Periegopidae
Ochyroceratidae
Scytodidae
Leptonetidae
Austrochilidae (Austrochilus)
Austrochilidae (Hickmania)
Gradungulidae
Eresidae
Anapidae
Theridiidae
Mysmenidae
Malkaridae
Mimetidae
Arkyidae
Tetragnathidae
Synaphridae
Cyatholipidae
Pimoidae
Linyphiidae
Synotaxidae
Nesticidae
Physoglenidae
Theridiosomatidae
Araneidae
Uloboridae
Hersiliidae
Oecobiidae
Deinopidae
Zodariidae
Sparassidae
Amaurobiidae
Agelenidae
Cybaeidae
Hahniidae
Cycloctenidae
Stiphidiidae
Desidae
Stiphidiidae
Zoropsidae
Senoculidae
Oxyopidae
Thomisidae
Ctenidae
Pisauridae
Trechaleidae
Lycosidae
Miturgidae
Selenopidae
Salticidae
Corinnidae
Clubionidae
Trochanteriidae
Gnaphosidae (Sergiolus)
Trachelidae
Liocranidae
Gnaphosidae (Molycria)
Lamponidae
Gnaphosidae (Anzacia)
Liphistiidae
Atypidae
Megahexuridae
Antrodiaetidae
Dipluridae
Porrhothelidae
Paratropidae
Macrothelidae
Nemesiidae
Theraphosidae
Halonoproctidae
Idiopidae
Euctenizidae
Nemesiidae
Filistatidae
Hypochilidae
Caponiidae
Segestriidae
Oonopidae
Dysderidae
Orsolobidae
Diguetidae
Pholcidae
Sicariidae
Drymusidae
Periegopidae
Scytodidae
Ochyroceratidae
Leptonetidae (Archoleptoneta)
Leptonetidae (Calileptoneta)
Austrochilidae (Austrochilus)
Austrochilidae (Hickmania)
Gradungulidae
Eresidae
Theridiidae
Anapidae
Synaphridae
Mysmenidae
Theridiosomatidae
Synotaxidae
Araneidae
Nesticidae
Physoglenidae
Cyatholipidae
Pimoidae
Linyphiidae
Malkaridae
Mimetidae
Arkyidae
Tetragnathidae
Uloboridae
Hersiliidae
Oecobiidae
Deinopidae
Zodariidae
Sparassidae
Amaurobiidae
Agelenidae
Cybaeidae
Hahniidae
Cycloctenidae
Stiphidiidae
Desidae
Stiphidiidae
Zoropsidae
Ctenidae
Senoculidae
Oxyopidae
Thomisidae
Pisauridae
Trechaleidae
Lycosidae
Miturgidae
Selenopidae
Corinnidae
Salticidae
Trochanteriidae
Clubionidae
Trachelidae
Liocranidae
Gnaphosidae (Sergiolus)
Gnaphosidae (Molycria)
Lamponidae
Gnaphosidae (Anzacia)

Supplementary Fig. 4. Schematic representation of the workflow of NOrthGen- Nucleotide Ortholog Generator modules (https://github.com/sskspider/NOrthGen) used to obtain map ortholog identifiers from amino acids to nucleotide data. ABC and DEF are taxon name exemplars. Dotted arrows indicate processes and red arrows indicate mandatory input files for using NOrthGen.

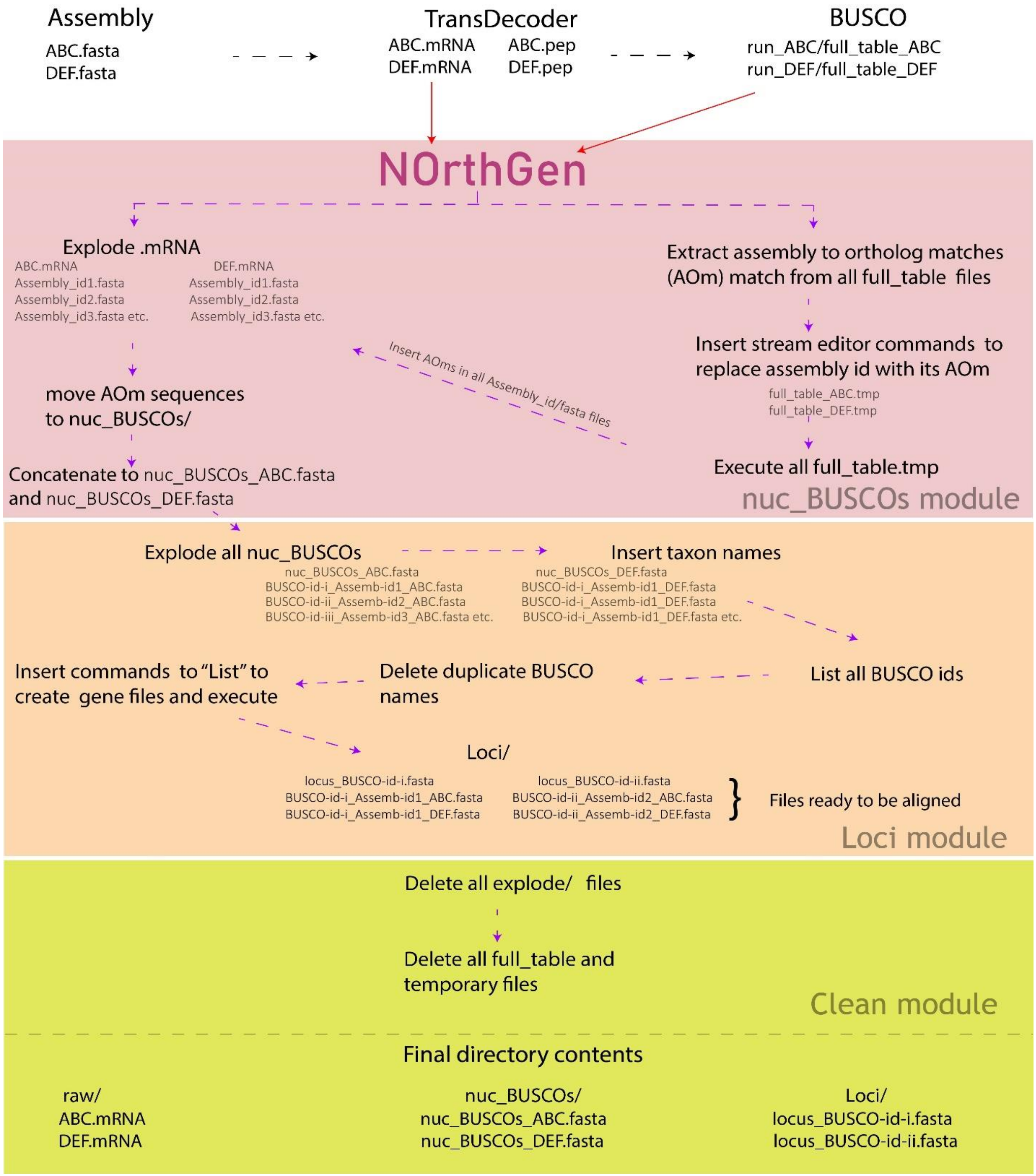

Supplementary Table 1. List of taxa with their source of data and the number of UCE loci recovered using the spider probe set. Abbreviations in the column datatype mean as follows: G- Genome, T- Transcriptomes and UCE- Ultraconserved elements.

**Family**	**Taxon**	**Reference**	**Accession code**	**Data type**	**UCE loci**
Agelenidae	*Agelenopsis emertoni*	Bond et al. 2014	SRR1514895	T	656
Agelenidae	*Agelenopsis pennsylvanica*	Fernández et al. 2014	SRR1329248	T	427
Antrodiaetidae	*Aliatypus coylei*	Bond et al. 2014	SRR1514876	T	838
Lycosidae	*Allocosa alticeps*	Fernández et al. 2018	SRR6997626	T	409
Amaurobiidae	*Amaurobius ferox*	Fernández et al. 2014	SRR1329250	T	325
Desidae	*Amphinecta* sp.	Fernández et al. 2018	SRR6997628	T	341
Ctenidae	*Anahita punctulata*	Garrison et al. 2016	SRR3144072	T	736
Anapidae	*Anapidae* sp.	Kulkarni et al. 2020	SRR10291826	U	28
Anapidae	*Anapis* sp.	Kulkarni et al. 2020	SRR10291825	U	133
Anapidae	*Anapisona hamigera*	Kulkarni et al. 2020	SRR10291824	U	55
Anapidae	*Chasmocephalon* sp.	Kulkarni et al. 2020	SRR10291816	U	523
Anapidae	*Crassanapis chilensis*	Kulkarni et al. 2020	SRR10291870	U	354
Anapidae	*Maxanapis bartle*	Kulkarni et al. 2020	SRR10291853	U	531
Theridiidae	*Anelosimus eximius*	Fernández et al. 2018	SRR6997627	T	628
Anapidae	*Novanapis spinipes*	Kulkarni et al. 2020	SRR10291847	U	380
Anapidae	*Paranapis* sp.	Kulkarni et al. 2020	SRR10291822	U	35
Anapidae	*Sofanapis antillanca*	Kulkarni et al. 2020	SRR10291837	U	643
Antrodiaetidae	*Antrodiaetus unicolor*	Bond et al. 2014	SRR1514897	T	724
Anapidae	*Zealanapis* sp.	Kulkarni et al. 2020	SRR10291830	U	440
Gnaphosidae	*Anzacia* sp.	Fernández et al. 2018	SRR6997629	T	356

Theraphosidae	*Aphonopelma iviei*	Bond et al. 2014	SRR1514871	T	403
Antrodiaetidae	*Atypoides riversi*	Starrett et al. 2017	SRR3932800	U	138
Antrodiaetidae	*Atypoides riversi*	Starrett et al. 2017	SRR3932811	U	136
Araneidae	*Araneus marmoreus*	Kulkarni et al. 2020	SRR10291862	U	899
Araneidae	*Caerostris sexcuspidata*	Kulkarni et al. 2020	SRR10291817	U	1009
Euctenizidae	*Aptostichus stephencolberti*	Bond et al. 2014	SRR1514874	T	270
Leptonetidae	*Archoleptoneta* sp.	Fernández et al. 2018	SRR6997753	T	350
Arkyidae	*Arkys* sp.	Fernández et al. 2018	SRR6997752	T	381
Mimetidae	*Australomimetus sp.*	Fernández et al. 2018	SRR6997751	T	496
Archaeidae	*Austrarchaea nr. milledgei*	Fernández et al. 2018	SRR6997750	T	225
Austrochilidae	*Austrochilus forsteri*	Fernández et al. 2018	SRR6997749	T	494
Araneidae	*Trichonephila clavipes*	Babb et al. 2017	GCA_002102615.1	G	1431
Araneidae	*Paraplectanoides crassipes*	Kulkarni et al. 2020	SRR10291845	U	876
Theridiosomatidae	*Baalzebub sp.*	Fernández et al. 2018	SRR6997748	T	257
Araneidae	*Zygiella* sp.	Kulkarni et al. 2020	SRR10291827	U	971
Archaeidae	*Austrarchaea* sp.	Kulkarni et al. 2020	SRR10291828	U	929
Nemesiidae	*Brachythele longitarsus*	Bond et al. 2014	SRR1514875	T	605
Archaeidae	*Eriauchenius* sp.	Kulkarni et al. 2020	SRR10291867	U	837
Arkyidae	*Arkys* sp.	Kulkarni et al. 2020	SRR10291850	U	474
Sparassidae	*Caayguara ybytyriguara*	Fernández et al. 2018	SRR6997747	T	211
Leptonetidae	*Calileptoneta californica*	Garrison et al. 2016	SRR3144085	T	897
Amaurobiidae	*Callobius* sp.	Garrison et al. 2016	SRR3144088	T	554
Austrochilidae	Austrochilidae sp.	Wood et al. 2018	SRR7387522	U	130
Austrochilidae	*Austrochilus forsteri*	Kulkarni et al. 2020	SRR10291819	U	962

Caponiidae	*Calponia harrisonfordi*	Garrison et al. 2016	SRR3144089	T	631
Cybaeidae	*Calymmaria persica*	Garrison et al. 2016	SRR3144091	T	759
Cybaeidae	*Calymmaria* sp.	Fernández et al. 2018	SRR6997746	T	366
Desidae	*Cambridgea* sp.	Fernández et al. 2018	SRR6997745	T	327
Synaphridae	*Cepheia longiseta*	Fernández et al. 2018	SRR6997742	T	416
Outgroup	*Chaerilus celebensis*	Sharma et al. 2015	SRR1721804	T	452
Dictynidae	*Cicurina travisae*	Hedin 2015	SRR1654705	T	362
Dictynidae	*Cicurina vobora*	Bond et al. 2014	SRR1514883	T	314
Ctenidae	*Phoneutria* sp.	Kulkarni et al. 2020	SRR10291843	U	914
Clubionidae	*Clubiona* sp.	Fernández et al. 2018	SRR6997741	T	454
Trechaleidae	*Cupiennius coccineus*	Fernández et al. 2018	SRR7028538	T	343
Halonoproctidae	*Hebestatis theveneti*	Starrett et al. 2017	SRR3932816	U	158
Cyatholipidae	*Cyatholipus* sp.	Kulkarni et al. 2020	SRR10291823	U	39
Cyatholipidae	*Tekelloides* sp.	Kulkarni et al. 2020	SRR10291834	U	761
Zodariidae	*Cybaeodamus taim*	Fernández et al. 2018	SRR6997744	T	245
Halonoproctidae	*Cyclocosmia truncata*	Bond et al. 2014	SRR1514884	T	644
Cyatholipidae	*Teemenaarus* sp.	Kulkarni et al. 2020	SRR10291820	U	50
Cycloctenidae	*Cycloctenus* sp.	Fernández et al. 2018	SRR6997743	T	267
Araneidae	*Cyrtophora* sp.	Fernández et al. 2018	SRR6997738	T	397
Deinopidae	*Deinopis* sp.	Kulkarni et al. 2020	SRR10291868	U	1066
Nemesiidae	*Damarchus* sp.	Bond et al. 2014	SRR3144092	T	359
Outgroup	*Damon variegatus*	Sharma et al. 2014	SRR1145694	T	284
Deinopidae	*Deinopis longipes*	Bond et al. 2014	SRR1514879	T	70
Deinopidae	*Deinopis* sp.	Fernández et al. 2018	SRR6997737	T	290
Arkyidae	*Demadiana* sp.	Fernández et al. 2018	SRR6997740	T	271

Diguetidae	*Diguetia* sp.	Garrison et al. 2016	SRR3144093	T	617
Diguetidae	*Diguetia signata*	Starrett et al. 2017	SRR3932813	U	142
Pisauridae	*Dolomedes triton*	Garrison et al. 2016	SRR3144094	T	676
Dipluridae	*Euagrus chisoeus*	Starrett et al. 2017	SRR3932814	U	170
Drymusidae	*Drymusa* sp.	Fernández et al. 2018	SRR6997739	T	264
Dysderidae	*Dysdera crocota*	Fernández et al. 2014	SRR1328258	T	403
Mimetidae	*Ero leonina*	Bond et al. 2014	SRR1514886	T	607
Eresidae	*Stegodyphus mimosarum*	Sanggaard et al. 2014	SRR1015314	T	272
Eresidae	*Stegodyphus mimosarum*	Sanggaard et al. 2014	GCA_000611955.2	G	1543
Theridiidae	*Euryopis* sp.	Fernández et al. 2018	SRR6997867	T	298
Corinnidae	*Falconina gracilis*	Fernández et al. 2018	SRR6997866	T	284
Filistatidae	*Kukulcania* sp.	Kulkarni et al. 2020	SRR10291859	U	353
Filistatidae	*Kukulcania* sp.	Starrett et al. 2017	SRR3932790	U	113
Filistatidae	*Filistata insidiatrix*	Fernández et al. 2018	SRR6997865	T	270
Zodariidae	*Forsterella* sp.	Fernández et al. 2018	SRR6997864	T	399
Linyphiidae	*Frontinella communis*	Sharma et al. 2014	SRR1145739	T	338
Gradungulidae	*Gradungula* sp.	Kulkarni et al. 2020	SRR10291863	U	1081
Araneidae	*Gasteracantha hasselti*	Zhao et al 2014	SRR1048659	T	641
Gradungulidae	*Gradungula sorenseni*	Fernández et al. 2018	SRR6997863	T	366
Salticidae	*Habronattus signatus*	Bond et al. 2014	SRR1514888	T	476
Halonoproctidae	*Hebestatis theveneti*	Bond et al. 2014	SRR1514887	T	520
Austrochilidae	*Hickmania troglodytes*	Fernández et al. 2018	SRR6997862	T	438
Homalonychidae	*Homalonychus theologus*	Garrison et al. 2016	SRR3144075	T	530
Huttoniidae	*Huttonia palpimanoides*	Fernández et al. 2018	SRR6997861	T	320
Hexathelidae	*Hexathele* sp.	Kulkarni et al. 2020	SRR10291836	U	449

Hypochilidae	*Hypochilus gertschi*	Fernández et al. 2018	SRR6997860	T	399
Hypochilidae	*Hypochilus pococki*	Bond et al. 2014	SRR1514889	T	62
Huttoniidae	*Huttonia* sp.	Kulkarni et al. 2020	SRR10291861	U	852
Idiopidae	*Idiops bersebaensis*	Bond et al. 2014	SRR1514907	T	475
Oonopidae	*Ischnothyreus* sp.	Fernández et al. 2018	SRR6997859	T	322
Hypochilidae	*Hypochilus gertschi*	Kulkarni et al. 2020	SRR10291860	U	971
Selenopidae	*Karaops raveni (juv)*	Fernández et al. 2018	SRR6997858	T	378
Hypochilidae	*Hypochilus pococki*	Starrett et al. 2017	SRR3932817	U	140
Filistatidae	*Kukulcania hibernalis*	Bond et al. 2014	SRR1514878	T	832
Linyphiidae	*Laminacauda parvipalpis*	Fernández et al. 2018	SRR6997873	T	475
Lamponidae	*Lampona* sp.	Fernández et al. 2018	SRR6997872	T	398
Theridiidae	*Latrodectus tredecimguttatus*	He et al. 2013	SRR954929	T	486
Tetragnathidae	*Leucauge venusta*	Sharma et al. 2014	SRR1145740	T	376
Linyphiidae	*Frontinella pyramitela*	Kulkarni et al. 2020	SRR10291865	U	638
Linyphiidae	*Laminacauda* sp.	Kulkarni et al. 2020	SRR10291858	U	905
Liocranidae	*Liocranidae* sp.	Fernández et al. 2018	SRR6997875	T	327
Liphistiidae	*Liphistius malayanus*	Sharma et al 2014	SRR1145736	T	355
Liphistiidae	*Liphistius* sp.	Bond et al 2014	SRR1514873	T	100
Liphistiidae	*Liphistius malayanus*	Starrett et al. 2017	SRR3932791	U	130
Sicariidae	*Loxosceles deserta*	Garrison et al. 2016	SRR3144077	T	866
Araneidae	*Macracantha arcuata*	Zhao et al 2014	SRR1048826	T	640
Macrothelidae	*Macrothele calpeiana*	Fernández et al. 2018	SRR6994009	T	140
Malkaridae	Malkaridae sp.09	Fernández et al. 2018	SRR6997874	T	430
Malkaridae	Malkaridae sp.17	Fernández et al. 2018	SRR6997869	T	248
Orsolobidae	*Maoriata* sp.	Fernández et al. 2018	SRR6997868	T	369

Malkaridae	Malkaridae sp.09	Kulkarni et al. 2020	SRR10291855	U	758
Outgroup	*Mastigoproctus giganteus*	Sharma et al. 2014	SRR1145698	T	506
Mecysmaucheniidae	*Mecysmauchenius* sp.	Fernández et al. 2018	SRR6997871	T	324
Megadictynidae	*Megadictyna thilenii*	Fernández et al. 2018	SRR6997870	T	386
Mecysmaucheniidae	*Semecysmauchenius* sp.	Kulkarni et al. 2020	SRR10291840	U	848
Mecysmaucheniidae	*Zearchaea* sp.	Kulkarni et al. 2020	SRR10291829	U	858
Megadictynidae	*Megadictyna thilenii*	Kulkarni et al. 2020	SRR10291852	U	920
Megahexuridae	*Megahexura fulva*	Bond et al. 2014	SRR1514891	T	861
Deinopidae	*Menneus* sp.	Fernández et al. 2018	SRR6997878	T	434
Megahexuridae	*Megahexura fulva*	Starrett et al. 2017	SRR8519232	U	148
Mimetidae	*Australomimetus* sp.	Kulkarni et al. 2020	SRR10291839	U	650
Physoglenidae	*Meringa* sp.	Fernández et al. 2018	SRR6997877	T	365
Tetragnathidae	*Meta ovalis*	Fernández et al. 2018	SRR6997876	T	415
Mimetidae	*Gelanor* sp.	Kulkarni et al. 2020	SRR10291864	U	767
Desidae	*Metaltella simoni*	Garrison et al. 2016	SRR3144079	T	574
Araneidae	*Micrathena gracilis*	Bond et al. 2014	SRR1514882	T	134
Mysmenidae	*Microdipoena guttata*	Kulkarni et al. 2020	SRR10291851	U	613
Mysmenidae	*Microdipoena guttata*	Fernández et al. 2014	SRR1333842	T	394
Dipluridae	*Microhexura montivaga*	Bond et al. 2014	SRR1514890	T	568
Thomisidae	*Misumenoides formosipes*	Garrison et al. 2016	SRR3144080	T	369
Nemesiidae	*Calisoga* sp.	Starrett et al. 2017	SRR3932812	U	151
Gnaphosidae	*Molycria* sp.	Fernández et al. 2018	SRR6998664	T	426
Mysmenidae	*Mysmena leichardti*	Fernández et al. 2018	SRR6998665	T	471
Mysmenidae	*Mysmenella jobi*	Fernández et al. 2018	SRR6998666	T	378

Nesticidae	*Nesticella* sp.	Kulkarni et al. 2020	SRR10291849	U	571
Tetragnathidae	*Nanometa* sp.	Fernández et al. 2018	SRR6998667	T	415
Agelenidae	*Neoramia* sp.	Fernández et al. 2018	SRR6998660	T	428
Nesticidae	*Nesticus gertschi*	Starrett et al. 2017	SRR3932793	U	128
Araneidae	*Neoscona arabesca*	Sharma et al. 2014	SRR1145741	T	378
Nicodamidae	Nicodamidae *sp.*	Kulkarni et al. 2020	SRR10291848	U	1064
Nesticidae	*Nesticus bishopi*	Hedin 2015	SRR1655191	T	752
Nesticidae	*Nesticus cooperi*	Bond et al. 2014	SRR1514892	T	74
Nicodamidae	Nicodamidae sp. 1	Fernández et al. 2018	SRR7028539	T	423
Nicodamidae	Nicodamidae_sp 2	Fernández et al. 2018	SRR6998661	T	564
Oecobiidae	*Oecobius navus*	Starrett et al. 2017	SRR3932794	U	154
Linyphiidae	*Novafroneta* sp. *(*nr. *gladiatrix)*	Fernández et al. 2018	SRR6998662	T	467
Anapidae	*Novanapis* sp.	Fernández et al. 2018	SRR6998663	T	226
Orsolobidae	*Maoriata* sp.	Kulkarni et al. 2020	SRR10291854	U	668
Corinnidae	*Nyssus* sp.	Fernández et al. 2018	SRR6998658	T	436
Ochyroceratidae	*Ochyrocera* sp.	Fernández et al. 2018	SRR7028536	T	357
Outgroup	*Bothriurus keyserlingi*	Starrett et al. 2017	SRR3932805	U	118
Outgroup	*Centruroides sculpturatus*	Starrett et al. 2017	SRR3932806	U	259
Oecobiidae	*Oecobius cellariorum*	Fernández et al. 2014	SRR1365089	T	375
Outgroup	*Damon diadema*	Starrett et al. 2017	SRR3932789	U	99
Oecobiidae	*Oecobius navus*	Bond et al. 2014	SRR1514899	T	830
Theridiosomatidae	*Ogulnius* sp.	Fernández et al. 2018	SRR7028537	T	393
Oonopidae	*Opopaea* sp.	Fernández et al. 2018	SRR6998659	T	517
Orsolobidae	Orsolobidae sp.(*Tangata*?)	Fernández et al. 2018	SRR6998651	T	474

Palpimanidae	*Otiothops birabeni*	Fernández et al. 2018	SRR6998652	T	386
Palpimanidae	*Palpimanus gibbulus*	Fernández et al. 2018	SRR6998653	T	527
Palpimanidae	Chediminae sp.	Kulkarni et al. 2020	SRR10291872	U	748
Outgroup	*Pandinus imperator*	Sharma et al. 2014	SRR1721600	T	400
Desidae	*Paramatachia* sp.	Fernández et al. 2018	SRR6998654	T	502
Malkaridae	*Pararchaea alba*	Fernández et al. 2018	SRR6998655	T	294
Paratropididae	*Paratropis* sp.	Bond et al. 2014	SRR1514893	T	355
Pholcidae	*Chibchea mapuche*	Kulkarni et al. 2020	SRR10291871	U	669
Periegopidae	*Periegops suterii*	Fernández et al. 2018	SRR6998656	T	419
Malkaridae	*Perissopmeros* sp.	Fernández et al. 2018	SRR6998657	T	436
Oxyopidae	*Peucetia longipalpis*	Bond et al. 2014	SRR1514898	T	677
Uloboridae	*Philoponella herediae*	Bond et al. 2014	SRR1514880	T	86
Physoglenidae	Physoglenidae sp.	Kulkarni et al. 2020	SRR10291821	U	99
Physoglenidae	*Runga* cf. *flora*	Kulkarni et al. 2020	SRR10291846	U	987
Pholcidae	*Pholcus manueli*	Fernández et al. 2014	SRR1365208	T	364
Pimoidae	*Pimoa breviata*	Kulkarni et al. 2020	SRR10291842	U	871
Pholcidae	*Pholcus phalangioides*	Bond et al. 2014	SRR3144082	T	659
Physoglenidae	*Physoglenes* sp.	Fernández et al. 2018	SRR6998913	T	252
Gradungulidae	*Pianoa isolata*	Fernández et al. 2018	SRR6998914	T	320
Pimoidae	*Pimoa* sp.1	Garrison et al. 2016	SRR3144083	T	795
Pimoidae	*Pimoa* sp.2	Fernández et al. 2018	SRR6998915	T	448
Porrhothelidae	*Porrhothele* sp.	Starrett et al. 2017	SRR3932795	U	146
Nemesiidae	*Pionothele* sp.	Bond et al. 2014	SRR1514906	T	417
Pisauridae	*Pisaurina mira*	Fernández et al. 2014	SRR1365651	T	326
Salticidae	*Habronattus tarsalis*	Starrett et al. 2017	SRR3932815	U	112
Salticidae	*Habronattus ustulatus*	Hedin 2015	SRR1656783	T	762

Scytodidae	*Scytodes* sp.	Kulkarni et al. 2020	SRR10291841	U	724
Porrhothelidae	*Porrhothele* sp.	Fernández et al. 2018	SRR6997604	T	448
Gradungulidae	*Progradungula otwayensis*	Fernández et al. 2018	SRR6998916	T	395
Segestriidae	*Segestria* sp.	Wood et al. 2018	SRR7363162	U	78
Euctenizidae	*Promyrmekiaphila clathrata*	Bond et al. 2014	SRR1514896	T	584
Trochanteriidae	*Rebilus* sp.	Fernández et al. 2018	SRR6998909	T	612
Physoglenidae	*Runga* sp.	Fernández et al. 2018	SRR6998910	T	435
Lycosidae	*Schizocosa rovneri*	Bond et al. 2014	SRR1514894	T	807
Sicariidae	*Loxosceles reclusa*	i5k Consortium 2013	GCA_001188405.1	G	1416
Scytodidae	*Scytodes globula*	Fernández et al. 2018	SRR6998911	T	774
Stenochilidae	*Colopea* sp.	Wood et al. 2018	SRR7363181	U	120
Scytodidae	*Scytodes thoracica*	Bond et al. 2014	SRR1514872	T	201
Symphytognathidae	*Anapistula* sp.	Kulkarni et al. 2020	SRR10291857	U	475
Symphytognathidae	*Patu* sp.	Kulkarni et al. 2020	SRR10291844	U	594
Segestriidae	*Segestria* sp.	Garrison et al. 2016	SRR3144084	T	550
Senoculidae	*Senoculus* sp.	Fernández et al. 2018	SRR7028535	T	116
Synotaxidae	*Synotaxus* sp.	Kulkarni et al. 2020	SRR10291835	U	888
Gnaphosidae	*Sergiolus capulatus*	Bond et al. 2014	SRR1514903	T	800
Thomisidae	*Sidymella* sp.	Fernández et al. 2018	SRR6998912	T	390
Tetragnathidae	*Leucauge venusta*	Kulkarni et al. 2020	SRR10291856	U	672
Atypidae	*Sphodros rufipes*	Bond et al. 2014	SRR1514908	T	828
Anapidae	*Spinanapis* sp.	Fernández et al. 2018	SRR6998907	T	405
Nemesiidae	*Stanwellia* sp.	Fernández et al. 2018	SRR6997603	T	436
Theraphosidae	*Acanthoscurria geniculata*	PRJNA222762	SRR1024076	T	496
Outgroup	*Stenochrus* cf. *portoricensis*	Fernández et al. 2018	SRR6997625	T	498
Theridiidae	*Anelosimus analyticus*	Kulkarni et al. 2020	SRR10291873	U	941

Synotaxidae	*Synotaxus* cf. *turbinatus*	Fernández et al. 2018	SRR6998908	T	410
Theridiidae	*Euryopis* sp.	Kulkarni et al. 2020	SRR10291866	U	691
Outgroup	*Tachypleus tridentatus*	Li et al. 2015	SRR946952	T	495
Theridiidae	*Latrodectus hesperus*	i5k Consortium 2013	GCA_000697925.1	G	1498
Hersiliidae	*Tamopsis* sp.	Fernández et al. 2018	SRR6998917	T	475
Theridiidae	*Parasteatoda tepidariorum*	Schwager et al. 2017	GCA_000365465.1	G	1526
Gradungulidae	*Tarlina* sp.	Fernández et al. 2018	SRR6998918	T	480
Theridiidae	*Theridula* sp.	Kulkarni et al. 2020	SRR10291832	U	1393
Theridiosomatidae	*Baalzebub* sp.	Kulkarni et al. 2020	SRR10291818	U	767
Cyatholipidae	*Tekelloides* sp. 1	Fernández et al. 2018	SRR6998919	T	323
Cyatholipidae	*Tekelloides* sp. 2	Fernández et al. 2018	SRR6998920	T	328
Theridiosomatidae	*Theridiosoma* sp.	Kulkarni et al. 2020	SRR10291833	U	753
Miturgidae	*Teminius* sp.	Fernández et al. 2018	SRR6998921	T	404
Tengellidae	*Tengella radiata*	Fernández et al. 2018	SRR7028532	T	383
Theridiosomatidae	*Wendilgarda* sp.	Wood et al. 2018	SRR7363155	U	67
Tetragnathidae	*Tetragnatha tantalus*	Brewer et al 2015	SRR1427108	T	173
Thomisidae	*Sidymella* sp.	Kulkarni et al. 2020	SRR10291838	U	797
Theridiidae	*Theridion* sp.	Bond et al. 2014	SRR1514902	T	117
Theridiosomatidae	*Theridiosoma gemmosum*	Fernández et al. 2018	SRR6998922	T	370
Trechaleidae	*Cupiennius* sp.	Kulkarni et al. 2020	SRR10291869	U	995
Theridiosomatidae	*Theridiosoma savannum*	Fernández et al. 2018	SRR7028533	T	509
Trachelidae	*Trachelas tranquillus*	Fernández et al. 2014	SRR1329247	T	333
Araneidae	*Trichonephila clavipes*	Bond et al. 2014	SRR1514901	T	120
Theraphosidae	*Trichopelma laselva*	Bond et al. 2014	SRR1514881	T	536
Uloboridae	*Uloborus glomosus*	Kulkarni et al. 2020	SRR10291831	U	802

Zoropsidae	*Uliodon* sp.	Fernández et al. 2018	SRR6998923	T	417
Uloboridae	*Uloborus glomosus*	Fernández et al. 2014	SRR1328334	T	423
Araneidae	*Verrucosa arenata*	Garrison et al. 2016	SRR3144087	T	546
Uloboridae	*Zosis* sp.	Fernández et al. 2018	SRR6998924	T	510

Supplementary Table 2. Settings for different data sets used in phylogenetic analyses at minimum identity 65 and minimum coverage 65 used to match the Spider 2Kv1 probe set (Kulkarni et al. 2020) to contigs. Appended columns with black cells indicate relationships written in newick format recovered from the respective data set and the white cells indicate an alternative relationship.

Dataset	Occupancy/ Threshold	Taxa	Loci	Model for IQ-TREE	Composition	Mesothelae, Opisthothelae	Mygalomorphae, Araneomorphae	Atypoidea, Avicularioidea	Synspermiata (Hypochilidae, Filistatidae)	*Archoleptoneta* (Austrochiloidea, *Calileptoneta*)	Araneoidea (Eresidae, Nicodamoidea)	("symphytognathoids")	(UDOH,RTA)
codingUCEs	10	164	960	TVM+F+I+G4									
codingUCEs	25	164	600	GTR+F+I+G4	DNA sequences of the coding UCEs								
codingUCEs	50	164	52	GTR+F+I+G4									
AAUCEs	10	164	960	HIVw+F+I+G4									
AAUCEs	25	164	600	HIVw+F+I+G4	Sequences from codingUCEs translated to amino acids								
AAUCEs	50	164	52	HIVw+F+I+G4									
AllUCEs	10	248	1060	TVM+F+I+G4	Nucleotide DNA sequences of the codingUCEs plus all UCEs from taxa included in Kulkarni et al. (2020)								
AllUCEs	25	248	597	GTR+F+I+G4									
AllAAUCEs	10	248	1060	HIVw+F+I+G4	The amino acid sequences for the taxa similar to AllUCEs datasets								
AllAAUCEs	25	248	597	HIVw+F+I+G4									
nucT	10	165	2474	GTR+F+I+G4									
nucT	25	165	2056	GTR+F+I+G4	Transcriptomes analyzed as nucleotides						incl. Megadictynidae		
nucT	50	165	1041	GTR+F+I+G4									
nucT	67	165	305	GTR+F+I+G4								Except Theridiosomatidae	
non-coding	>50 bp	56	1204	K3Pu+F+I+G4	non-coding regions obtained from UCE data set of Kulkarni et al. (2020)								
3RcodingUCEs	10	164	960	TVM+F+I+G4								Except Theridiosomatidae	
3RcodingUCEs	25	164	600	TVM+F+I+G4	Third codon removed of *codingUCE* data sets							Except Theridiosomatidae	
3RcodingUCEs	50	164	52	GTR+F+I+G4									
3RnucT	10	165	2474	GTR+F+I+G4									
3RnucT	25	165	2056	GTR+F+I+G4	Third codon removed from *nucT* data sets								
3RnucT	50	165	1041	GTR+F+I+G4									
3RnucT	67	165	305	GTR+F+I+G4								Except Theridiosomatidae	

Supplementary Table 3: Results of the topology tests conducted on the *AllUCEs* data set.

Tree	logL	deltaL	bp-RELL	p-KH	p-SH	c-ELW	p-AU
AllUCE10	-5685294.422	0	0.993 +	0.993 +	1 +	0.993 +	0.994 +
AllUCE25	-5685669.492	375.07	0.0072 -	0.0073 -	0.0073 -	0.0073 -	0.00617 -

deltaL : logL difference from the maximal logl in the set.
bp-RELL : bootstrap proportion using RELL method (Kishino et al. 1990).
p-KH : p-value of one sided Kishino-Hasegawa test (1989).
p-SH : p-value of Shimodaira-Hasegawa test (2000).
c-ELW : Expected Likelihood Weight (Strimmer & Rambaut 2002).
p-AU : p-value of approximately unbiased (AU) test (Shimodaira, 2002).

Plus signs denote the 95% confidence sets.
Minus signs denote significant exclusion.
All tests performed 10000 resamplings using the RELL method.

Supplementary trees. Phylogenetic relationships obtained for all data sets.

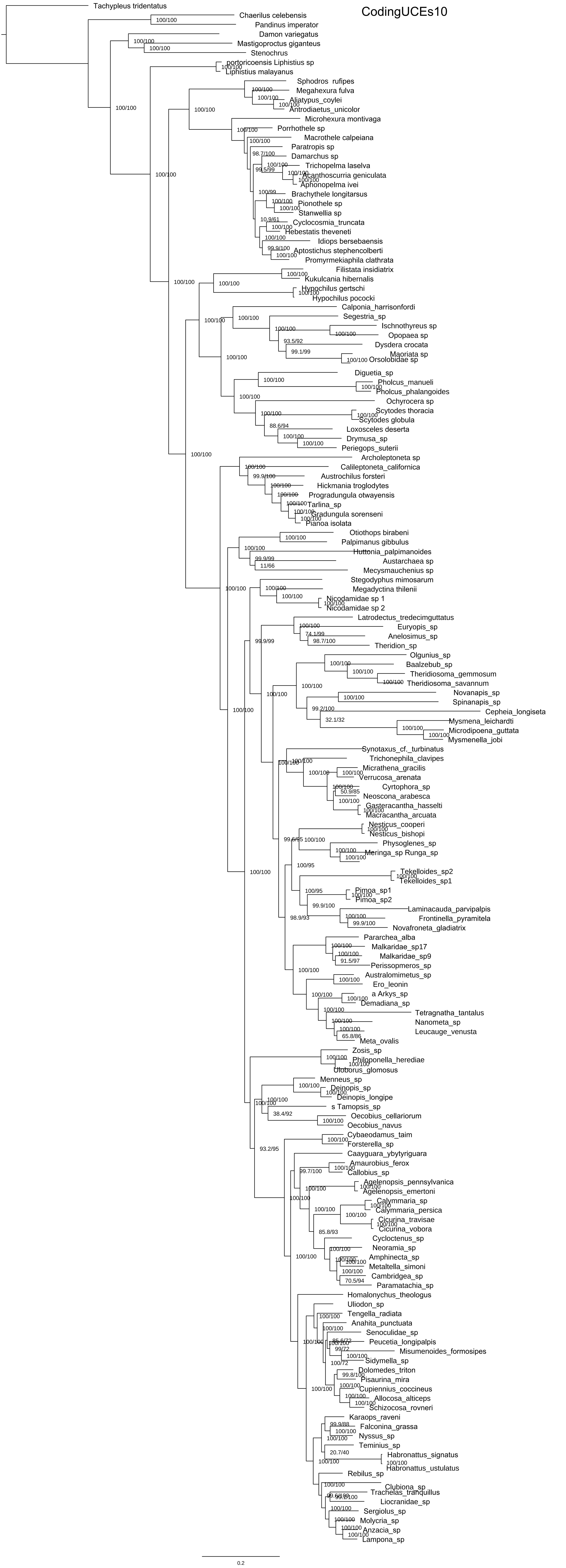
CodingUCEs10
Tachypleus tridentatus
Chaerilus celebensis
Pandinus imperator
Damon variegatus
Mastigoproctus giganteus
Stenochrus
portoricoensis Liphistius sp
Liphistius malayanus
Sphodros rufipes
Megahexura fulva
Aliatypus_coylei
Antrodiaetus_unicolor
Microhexura montivaga
Porrhothele sp
Macrothele calpeiana
Paratropis sp
Damarchus sp
Trichopelma laselva
Acanthoscurria geniculata
Aphonopelma ivei
Brachythele longitarsus
Pionothele sp
Stanwellia sp
Cyclocosmia_truncata
Hebestatis theveneti
Idiops bersebaensis
Aptostichus stephencolberti
Promyrmekiaphila clathrata
Filistata insidiatrix
Kukulcania hibernalis
Hypochilus gertschi
Hypochilus pococki
Calponia_harrisonfordi
Segestria_sp
Ischnothyreus sp
Opopaea sp
Dysdera crocata
Maoriata sp
Orsolobidae sp
Diguetia_sp
Pholcus_manueli
Pholcus_phalangoides
Ochyrocera sp
Scytodes thoracia
Scytodes globula
Loxosceles deserta
Drymusa_sp
Periegops_suterii
Archoleptoneta sp
Calileptoneta_californica
Austrochilus forsteri
Hickmania troglodytes
Progradungula otwayensis
Tarlina_sp
Gradungula sorenseni
Pianoa isolata
Otiothops birabeni
Palpimanus gibbulus
Huttonia_palpimanoides
Austarchaea sp
Mecysmauchenius sp
Stegodyphus mimosarum
Megadyctina thilenii
Nicodamidae sp 1
Nicodamidae sp 2
Latrodectus_tredecimguttatus
Euryopis_sp
Anelosimus_sp
Theridion_sp
Olgunius_sp
Baalzebub_sp
Theridiosoma_gemmosum
Theridiosoma_savannum
Novanapis_sp
Spinanapis_sp
Cepheia_longiseta
Mysmena_leichardti
Microdipoena_guttata
Mysmenella_jobi
Synotaxus_cf._turbinatus
Trichonephila_clavipes
Micrathena_gracilis
Verrucosa_arenata
Cyrtophora_sp
Neoscona_arabesca
Gasteracantha_hasselti
Macracantha_arcuata
Nesticus_cooperi
Nesticus_bishopi
Physoglenes_sp
Meringa_sp Runga_sp
Tekelloides_sp2
Tekelloides_sp1
Pimoa_sp1
Pimoa_sp2
Laminacauda_parvipalpis
Frontinella_pyramitela
Novafroneta_gladiatrix
Pararchea_alba
Malkaridae_sp17
Malkaridae_sp9
Perissopmeros_sp
Australomimetus_sp
Ero_leonin
a Arkys_sp
Demadiana_sp
Tetragnatha_tantalus
Nanometa_sp
Leucauge_venusta
Meta_ovalis
Zosis_sp
Philoponella_herediae
Uloborus_glomosus
Menneus_sp
Deinopis_sp
Deinopis_longipe
s Tamopsis_sp
Oecobius_cellariorum
Oecobius_navus
Cybaeodamus_taim
Forsterella_sp
Caayguara_ybytyriguara
Amaurobius_ferox
Callobius_sp
Agelenopsis_pennsylvanica
Agelenopsis_emertoni
Calymmaria_sp
Calymmaria_persica
Cicurina_travisae
Cicurina_vobora
Cycloctenus_sp
Neoramia_sp
Amphinecta_sp
Metaltella_simoni
Cambridgea_sp
Paramatachia_sp
Homalonychus_theologus
Uliodon_sp
Tengella_radiata
Anahita_punctuata
Senoculidae_sp
Peucetia_longipalpis
Misumenoides_formosipes
Sidymella_sp
Dolomedes_triton
Pisaurina_mira
Cupiennius_coccineus
Allocosa_alticeps
Schizocosa_rovneri
Karaops_raveni
Falconina_grassa
Nyssus_sp
Teminius_sp
Habronattus_signatus
Habronattus_ustulatus
Rebilus_sp
Clubiona_sp
Trachelas_tranquillus
Liocranidae_sp
Sergiolus_sp
Molycria_sp
Anzacia_sp
Lampona_sp
0.2

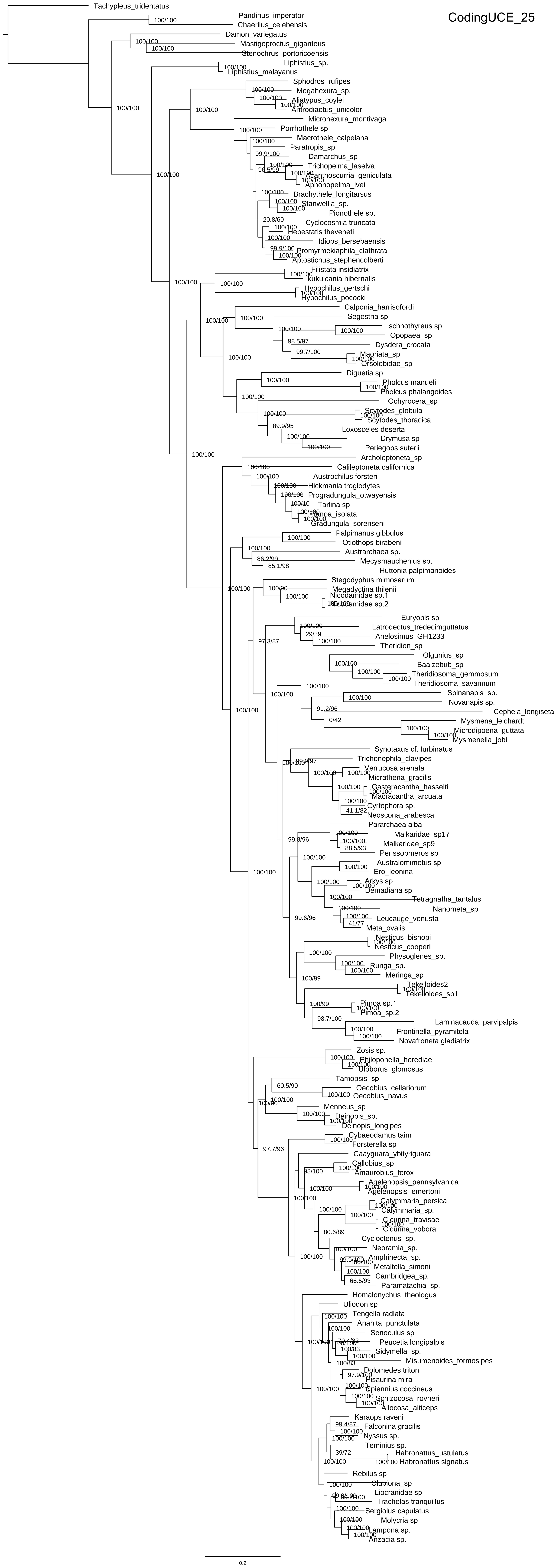

CodingUCE_25
Tachypleus_tridentatus
Pandinus_imperator
Chaerilus_celebensis
Damon_variegatus
Mastigoproctus_giganteus
Stenochrus_portoricoensis
Liphistius_sp.
Liphistius_malayanus
Sphodros_rufipes
Megahexura_sp.
Aliatypus_coylei
Antrodiaetus_unicolor
Microhexura_montivaga
Porrhothele sp
Macrothele_calpeiana
Paratropis_sp
Damarchus_sp
Trichopelma_laselva
Acanthoscurria_geniculata
Aphonopelma_iveі
Brachythele_longitarsus
Stanwellia_sp.
Pionothele sp.
Cyclocosmia truncata
Hebestatis theveneti
Idiops_bersebaensis
Promyrmekiaphila_clathrata
Aptostichus_stephencolberti
Filistata insidiatrix
kukulcania hibernalis
Hypochilus_gertschi
Hypochilus_pococki
Calponia_harrisofordi
Segestria sp
ischnothyreus sp
Opopaea_sp
Dysdera_crocata
Maoriata_sp
Orsolobidae_sp
Diguetia sp
Pholcus manueli
Pholcus phalangoides
Ochyrocera_sp
Scytodes_globula
Scytodes_thoracica
Loxosceles deserta
Drymusa sp
Periegops suterii
Archoleptoneta_sp
Calileptoneta californica
Austrochilus forsteri
Hickmania troglodytes
Progradungula_otwayensis
Tarlina sp
Pianoa_isolata
Gradungula_sorenseni
Palpimanus gibbulus
Otiothops birabeni
Austrarchaea sp.
Mecysmauchenius sp.
Huttonia palpimanoides
Stegodyphus mimosarum
Megadyctina thilenii
Nicodamidae sp.1
Nicodamidae sp.2
Euryopis sp
Latrodectus_tredecimguttatus
Anelosimus_GH1233
Theridion_sp
Olgunius_sp
Baalzebub_sp
Theridiosoma_gemmosum
Theridiosoma_savannum
Spinanapis sp.
Novanapis sp.
Cepheia_longiseta
Mysmena_leichardti
Microdipoena_guttata
Mysmenella_jobi
Synotaxus cf. turbinatus
Trichonephila_clavipes
Verrucosa arenata
Micrathena_gracilis
Gasteracantha_hasselti
Macracantha_arcuata
Cyrtophora sp.
Neoscona_arabesca
Pararchaea alba
Malkaridae_sp17
Malkaridae_sp9
Perissopmeros sp
Australomimetus sp
Ero_leonina
Arkys sp
Demadiana sp
Tetragnatha_tantalus
Nanometa_sp
Leucauge_venusta
Meta_ovalis
Nesticus_bishopi
Nesticus_cooperi
Physoglenes_sp.
Runga_sp.
Meringa_sp
Tekelloides2
Tekelloides_sp1
Pimoa sp.1
Pimoa_sp.2
Laminacauda parvipalpis
Frontinella_pyramitela
Novafroneta gladiatrix
Zosis sp.
Philoponella_herediae
Uloborus glomosus
Tamopsis_sp
Oecobius cellariorum
Oecobius_navus
Menneus_sp
Deinopis_sp.
Deinopis_longipes
Cybaeodamus taim
Forsterella sp
Caayguara_ybityriguara
Callobius_sp
Amaurobius_ferox
Agelenopsis_pennsylvanica
Agelenopsis_emertoni
Calymmaria_persica
Calymmaria_sp.
Cicurina_travisae
Cicurina_vobora
Cycloctenus_sp.
Neoramia_sp.
Amphinecta_sp.
Metaltella_simoni
Cambridgea_sp.
Paramatachia_sp.
Homalonychus theologus
Uliodon sp
Tengella radiata
Anahita punctulata
Senoculus sp
Peucetia longipalpis
Sidymella_sp.
Misumenoides_formosipes
Dolomedes triton
Pisaurina mira
Cpiennius coccineus
Schizocosa_rovneri
Allocosa_alticeps
Karaops raveni
Falconina gracilis
Nyssus sp.
Teminius sp.
Habronattus_ustulatus
Habronattus signatus
Rebilus sp
Clubiona_sp
Liocranidae sp
Trachelas tranquillus
Sergiolus capulatus
Molycria sp
Lampona sp.
Anzacia sp.
0.2

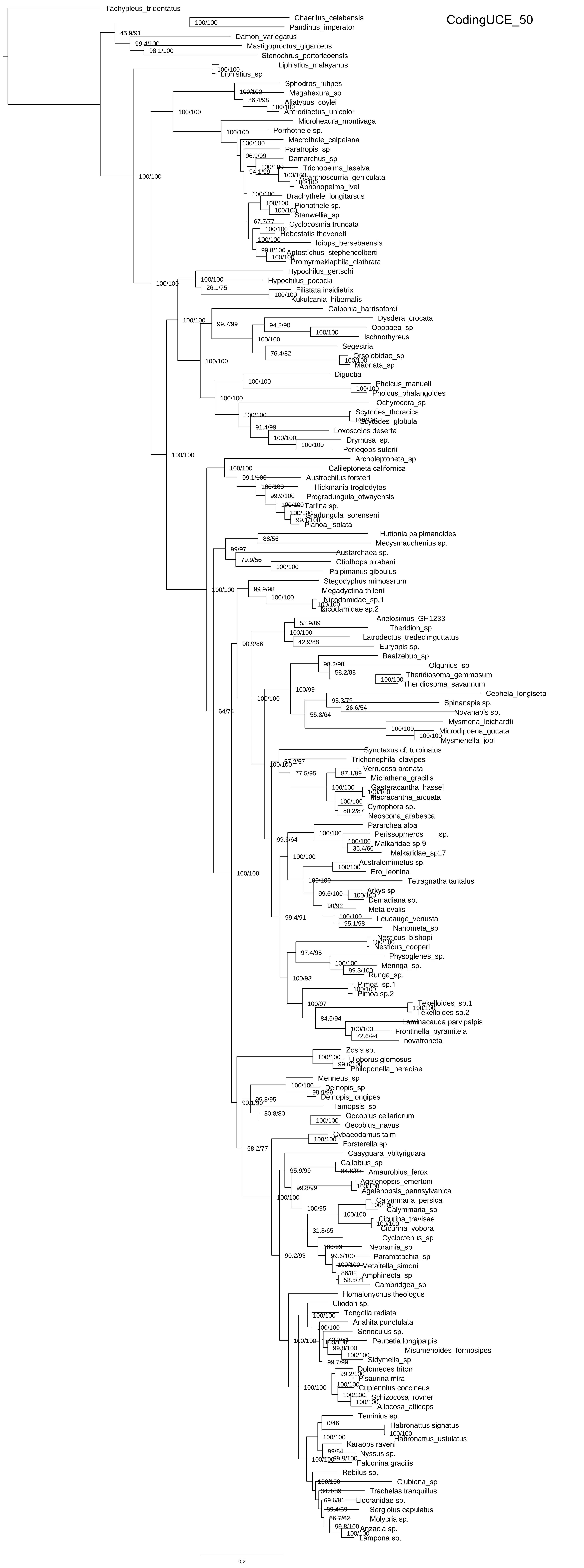
CodingUCE_50
Tachypleus_tridentatus
Chaerilus_celebensis
Pandinus_imperator
Damon_variegatus
Mastigoproctus_giganteus
Stenochrus_portoricoensis
Liphistius_malayanus
Liphistius_sp
Sphodros_rufipes
Megahexura_sp
Aliatypus_coylei
Antrodiaetus_unicolor
Microhexura_montivaga
Porrhothele sp.
Macrothele_calpeiana
Paratropis_sp
Damarchus_sp
Trichopelma_laselva
Acanthoscurria_geniculata
Aphonopelma_ivei
Brachythele_longitarsus
Pionothele sp.
Stanwellia_sp
Cyclocosmia truncata
Hebestatis theveneti
Idiops_bersebaensis
Aptostichus_stephencolberti
Promyrmekiaphila_clathrata
Hypochilus_gertschi
Hypochilus_pococki
Filistata insidiatrix
Kukulcania_hibernalis
Calponia_harrisofordi
Dysdera_crocata
Opopaea_sp
Ischnothyreus
Segestria
Orsolobidae_sp
Maoriata_sp
Diguetia
Pholcus_manueli
Pholcus_phalangoides
Ochyrocera_sp
Scytodes_thoracica
Scytodes_globula
Loxosceles deserta
Drymusa sp.
Periegops suterii
Archoleptoneta_sp
Calileptoneta californica
Austrochilus forsteri
Hickmania troglodytes
Progradungula_otwayensis
Tarlina sp.
Gradungula_sorenseni
Pianoa_isolata
Huttonia palpimanoides
Mecysmauchenius sp.
Austarchaea sp.
Otiothops birabeni
Palpimanus gibbulus
Stegodyphus mimosarum
Megadyctina thilenii
Nicodamidae_sp.1
Nicodamidae_sp.2
Anelosimus_GH1233
Theridion_sp
Latrodectus_tredecimguttatus
Euryopis sp.
Baalzebub_sp
Olgunius_sp
Theridiosoma_gemmosum
Theridiosoma_savannum
Cepheia_longiseta
Spinanapis sp.
Novanapis sp.
Mysmena_leichardti
Microdipoena_guttata
Mysmenella_jobi
Synotaxus cf. turbinatus
Trichonephila_clavipes
Verrucosa arenata
Micrathena_gracilis
Gasteracantha_hassel
Macracantha_arcuata
Cyrtophora sp.
Neoscona_arabesca
Pararchea alba
Perissopmeros sp.
Malkaridae sp.9
Malkaridae_sp17
Australomimetus sp.
Ero_leonina
Tetragnatha tantalus
Arkys sp.
Demadiana sp.
Meta ovalis
Leucauge_venusta
Nanometa_sp
Nesticus_bishopi
Nesticus_cooperi
Physoglenes_sp.
Meringa_sp.
Runga_sp.
Pimoa sp.1
Pimoa sp.2
Tekelloides_sp.1
Tekelloides sp.2
Laminacauda parvipalpis
Frontinella_pyramitela
novafroneta
Zosis sp.
Uloborus glomosus
Philoponella_herediae
Menneus_sp
Deinopis_sp
Deinopis_longipes
Tamopsis_sp
Oecobius cellariorum
Oecobius_navus
Cybaeodamus taim
Forsterella sp.
Caayguara_ybityriguara
Callobius_sp
Amaurobius_ferox
Agelenopsis_emertoni
Agelenopsis_pennsylvanica
Calymmaria_persica
Calymmaria_sp
Cicurina_travisae
Cicurina_vobora
Cycloctenus_sp
Neoramia_sp
Paramatachia_sp
Metaltella_simoni
Amphinecta_sp
Cambridgea_sp
Homalonychus theologus
Uliodon sp.
Tengella radiata
Anahita punctulata
Senoculus sp.
Peucetia longipalpis
Misumenoides_formosipes
Sidymella_sp
Dolomedes triton
Pisaurina mira
Cupiennius coccineus
Schizocosa_rovneri
Allocosa_alticeps
Teminius sp.
Habronattus signatus
Habronattus_ustulatus
Karaops raveni
Nyssus sp.
Falconina gracilis
Rebilus sp.
Clubiona_sp
Trachelas tranquillus
Liocranidae sp.
Sergiolus capulatus
Molycria sp.
Anzacia sp.
Lampona sp.
0.2

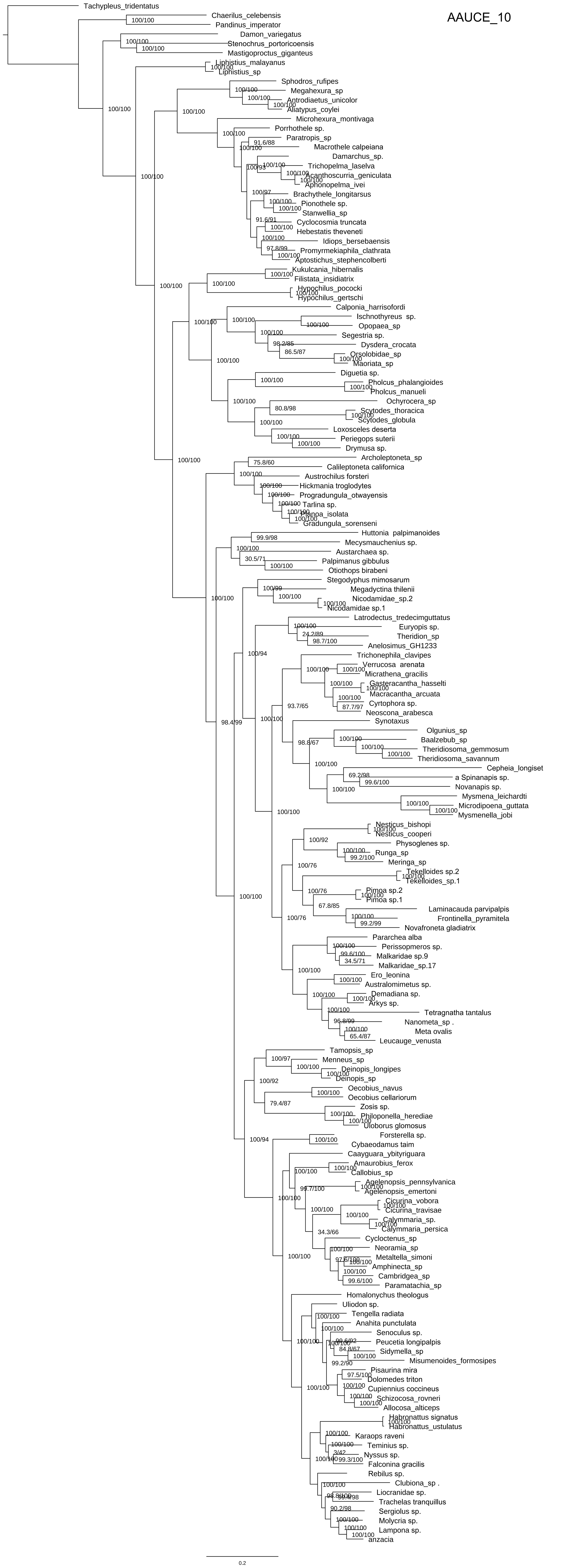
AAUCE_10
Tachypleus_tridentatus
Chaerilus_celebensis
Pandinus_imperator
Damon_variegatus
Stenochrus_portoricoensis
Mastigoproctus_giganteus
Liphistius_malayanus
Liphistius_sp
Sphodros_rufipes
Megahexura_sp
Antrodiaetus_unicolor
Aliatypus_coylei
Microhexura_montivaga
Porrhothele sp.
Paratropis_sp
Macrothele calpeiana
Damarchus_sp.
Trichopelma_laselva
Acanthoscurria_geniculata
Aphonopelma_ivei
Brachythele_longitarsus
Pionothele sp.
Stanwellia_sp
Cyclocosmia truncata
Hebestatis theveneti
Idiops_bersebaensis
Promyrmekiaphila_clathrata
Aptostichus_stephencolberti
Kukulcania_hibernalis
Filistata_insidiatrix
Hypochilus_pococki
Hypochilus_gertschi
Calponia_harrisofordi
Ischnothyreus sp.
Opopaea_sp
Segestria sp.
Dysdera_crocata
Orsolobidae_sp
Maoriata_sp
Diguetia sp.
Pholcus_phalangioides
Pholcus_manueli
Ochyrocera_sp
Scytodes_thoracica
Scytodes_globula
Loxosceles deserta
Periegops suterii
Drymusa sp.
Archoleptoneta_sp
Calileptoneta californica
Austrochilus forsteri
Hickmania troglodytes
Progradungula_otwayensis
Tarlina sp.
Pianoa_isolata
Gradungula_sorenseni
Huttonia palpimanoides
Mecysmauchenius sp.
Austarchaea sp.
Palpimanus gibbulus
Otiothops birabeni
Stegodyphus mimosarum
Megadyctina thilenii
Nicodamidae_sp.2
Nicodamidae sp.1
Latrodectus_tredecimguttatus
Euryopis sp.
Theridion_sp
Anelosimus_GH1233
Trichonephila_clavipes
Verrucosa arenata
Micrathena_gracilis
Gasteracantha_hasselti
Macracantha_arcuata
Cyrtophora sp.
Neoscona_arabesca
Synotaxus
Olgunius_sp
Baalzebub_sp
Theridiosoma_gemmosum
Theridiosoma_savannum
Cepheia_longiset
a Spinanapis sp.
Novanapis sp.
Mysmena_leichardti
Microdipoena_guttata
Mysmenella_jobi
Nesticus_bishopi
Nesticus_cooperi
Physoglenes sp.
Runga_sp
Meringa_sp
Tekelloides sp.2
Tekelloides_sp.1
Pimoa sp.2
Pimoa sp.1
Laminacauda parvipalpis
Frontinella_pyramitela
Novafroneta gladiatrix
Pararchea alba
Perissopmeros sp.
Malkaridae sp.9
Malkaridae_sp.17
Ero_leonina
Australomimetus sp.
Demadiana sp.
Arkys sp.
Tetragnatha tantalus
Nanometa_sp .
Meta ovalis
Leucauge_venusta
Tamopsis_sp
Menneus_sp
Deinopis_longipes
Deinopis_sp
Oecobius_navus
Oecobius cellariorum
Zosis sp.
Philoponella_herediae
Uloborus glomosus
Forsterella sp.
Cybaeodamus taim
Caayguara_ybityriguara
Amaurobius_ferox
Callobius_sp
Agelenopsis_pennsylvanica
Agelenopsis_emertoni
Cicurina_vobora
Cicurina_travisae
Calymmaria_sp.
Calymmaria_persica
Cycloctenus_sp
Neoramia_sp
Metaltella_simoni
Amphinecta_sp
Cambridgea_sp
Paramatachia_sp
Homalonychus theologus
Uliodon sp.
Tengella radiata
Anahita punctulata
Senoculus sp.
Peucetia longipalpis
Sidymella_sp
Misumenoides_formosipes
Pisaurina mira
Dolomedes triton
Cupiennius coccineus
Schizocosa_rovneri
Allocosa_alticeps
Habronattus signatus
Habronattus_ustulatus
Karaops raveni
Teminius sp.
Nyssus sp.
Falconina gracilis
Rebilus sp.
Clubiona_sp .
Liocranidae sp.
Trachelas tranquillus
Sergiolus sp.
Molycria sp.
Lampona sp.
anzacia
0.2

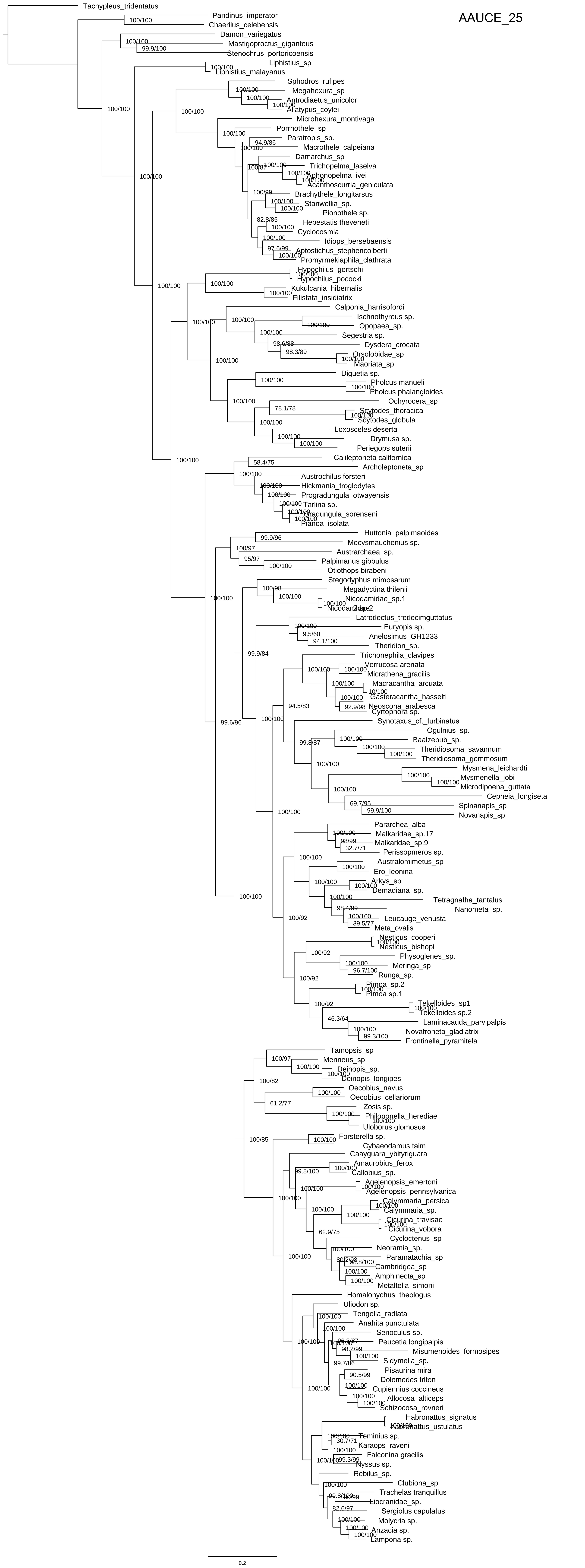
AAUCE_25
Tachypleus_tridentatus
Pandinus_imperator
Chaerilus_celebensis
Damon_variegatus
Mastigoproctus_giganteus
Stenochrus_portoricoensis
Liphistius_sp
Liphistius_malayanus
Sphodros_rufipes
Megahexura_sp
Antrodiaetus_unicolor
Aliatypus_coylei
Microhexura_montivaga
Porrhothele_sp
Paratropis_sp.
Macrothele_calpeiana
Damarchus_sp
Trichopelma_laselva
Aphonopelma_ivei
Acanthoscurria_geniculata
Brachythele_longitarsus
Stanwellia_sp.
Pionothele sp.
Hebestatis theveneti
Cyclocosmia
Idiops_bersebaensis
Aptostichus_stephencolberti
Promyrmekiaphila_clathrata
Hypochilus_gertschi
Hypochilus_pococki
Kukulcania_hibernalis
Filistata_insidiatrix
Calponia_harrisofordi
Ischnothyreus sp.
Opopaea_sp.
Segestria sp.
Dysdera_crocata
Orsolobidae_sp
Maoriata_sp
Diguetia sp.
Pholcus manueli
Pholcus phalangioides
Ochyrocera_sp
Scytodes_thoracica
Scytodes_globula
Loxosceles deserta
Drymusa sp.
Periegops suterii
Calileptoneta californica
Archoleptoneta_sp
Austrochilus forsteri
Hickmania_troglodytes
Progradungula_otwayensis
Tarlina sp.
Gradungula_sorenseni
Pianoa_isolata
Huttonia palpimaoides
Mecysmauchenius sp.
Austrarchaea sp.
Palpimanus gibbulus
Otiothops birabeni
Stegodyphus mimosarum
Megadyctina thilenii
Nicodamidae_sp.1
Nicodamidae_sp.2
Latrodectus_tredecimguttatus
Euryopis sp.
Anelosimus_GH1233
Theridion_sp.
Trichonephila_clavipes
Verrucosa arenata
Micrathena_gracilis
Macracantha_arcuata
Gasteracantha_hasselti
Neoscona_arabesca
Cyrtophora sp.
Synotaxus_cf._turbinatus
Ogulnius_sp.
Baalzebub_sp.
Theridiosoma_savannum
Theridiosoma_gemmosum
Mysmena_leichardti
Mysmenella_jobi
Microdipoena_guttata
Cepheia_longiseta
Spinanapis_sp
Novanapis_sp
Pararchea_alba
Malkaridae_sp.17
Malkaridae_sp.9
Perissopmeros sp.
Australomimetus_sp
Ero_leonina
Arkys_sp
Demadiana_sp.
Tetragnatha_tantalus
Nanometa_sp.
Leucauge_venusta
Meta_ovalis
Nesticus_cooperi
Nesticus_bishopi
Physoglenes_sp.
Meringa_sp
Runga_sp.
Pimoa_sp.2
Pimoa sp.1
Tekelloides_sp1
Tekelloides sp.2
Laminacauda_parvipalpis
Novafroneta_gladiatrix
Frontinella_pyramitela
Tamopsis_sp
Menneus_sp
Deinopis_sp.
Deinopis_longipes
Oecobius_navus
Oecobius cellariorum
Zosis sp.
Philoponella_herediae
Uloborus glomosus
Forsterella sp.
Cybaeodamus taim
Caayguara_ybityriguara
Amaurobius_ferox
Callobius_sp.
Agelenopsis_emertoni
Agelenopsis_pennsylvanica
Calymmaria_persica
Calymmaria_sp.
Cicurina_travisae
Cicurina_vobora
Cycloctenus_sp
Neoramia_sp.
Paramatachia_sp
Cambridgea_sp
Amphinecta_sp
Metaltella_simoni
Homalonychus theologus
Uliodon sp.
Tengella_radiata
Anahita punctulata
Senoculus sp.
Peucetia longipalpis
Misumenoides_formosipes
Sidymella_sp.
Pisaurina mira
Dolomedes triton
Cupiennius coccineus
Allocosa_alticeps
Schizocosa_rovneri
Habronattus_signatus
Habronattus_ustulatus
Teminius sp.
Karaops_raveni
Falconina gracilis
Nyssus sp.
Rebilus_sp.
Clubiona_sp
Trachelas tranquillus
Liocranidae_sp.
Sergiolus capulatus
Molycria sp.
Anzacia sp.
Lampona sp.
0.2

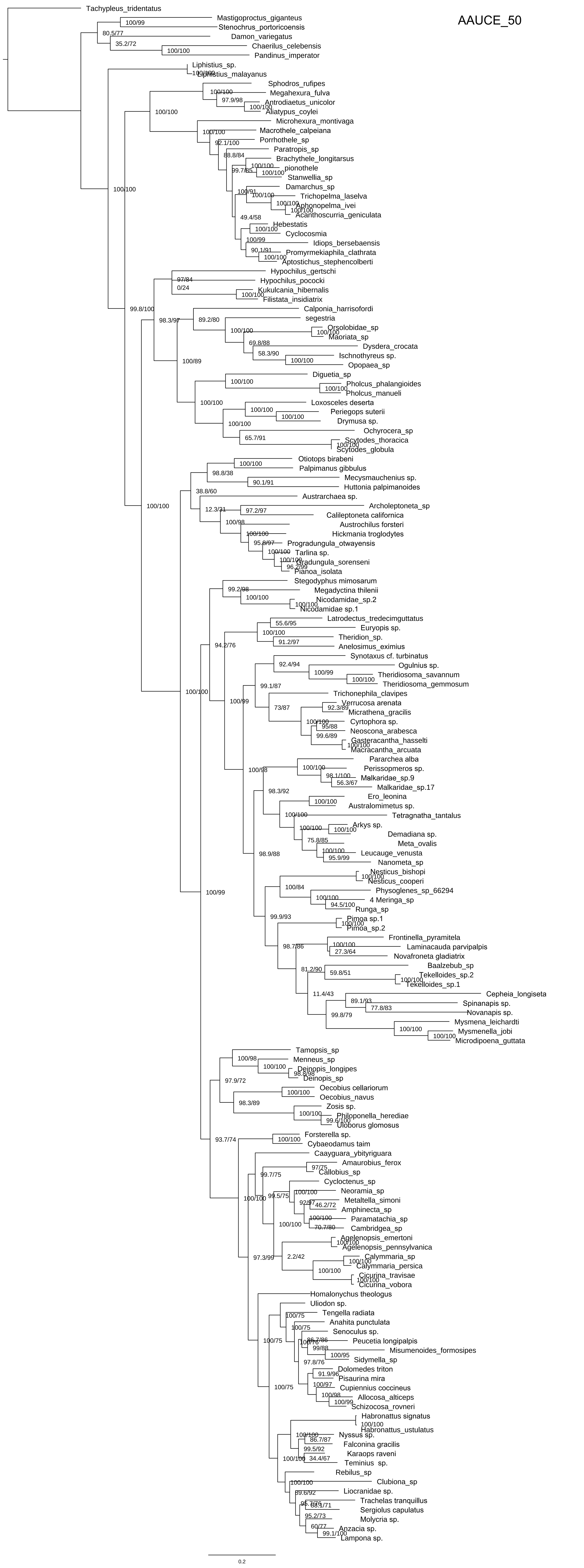

AAUCE_50
Tachypleus_tridentatus
Mastigoproctus_giganteus
Stenochrus_portoricoensis
Damon_variegatus
Chaerilus_celebensis
Pandinus_imperator
Liphistius_sp.
Liphistius_malayanus
Sphodros_rufipes
Megahexura_fulva
Antrodiaetus_unicolor
Aliatypus_coylei
Microhexura_montivaga
Macrothele_calpeiana
Porrhothele_sp
Paratropis_sp
Brachythele_longitarsus
pionothele
Stanwellia_sp
Damarchus_sp
Trichopelma_laselva
Aphonopelma_ivei
Acanthoscurria_geniculata
Hebestatis
Cyclocosmia
Idiops_bersebaensis
Promyrmekiaphila_clathrata
Aptostichus_stephencolberti
Hypochilus_gertschi
Hypochilus_pococki
Kukulcania_hibernalis
Filistata_insidiatrix
Calponia_harrisofordi
segestria
Orsolobidae_sp
Maoriata_sp
Dysdera_crocata
Ischnothyreus sp.
Opopaea_sp
Diguetia_sp
Pholcus_phalangioides
Pholcus_manueli
Loxosceles deserta
Periegops suterii
Drymusa sp.
Ochyrocera_sp
Scytodes_thoracica
Scytodes_globula
Otiotops birabeni
Palpimanus gibbulus
Mecysmauchenius sp.
Huttonia palpimanoides
Austrarchaea sp.
Archoleptoneta_sp
Calileptoneta californica
Austrochilus forsteri
Hickmania troglodytes
Progradungula_otwayensis
Tarlina sp.
Gradungula_sorenseni
Pianoa_isolata
Stegodyphus mimosarum
Megadyctina thilenii
Nicodamidae_sp.2
Nicodamidae sp.1
Latrodectus_tredecimguttatus
Euryopis sp.
Theridion_sp.
Anelosimus_eximius
Synotaxus cf. turbinatus
Ogulnius sp.
Theridiosoma_savannum
Theridiosoma_gemmosum
Trichonephila_clavipes
Verrucosa arenata
Micrathena_gracilis
Cyrtophora sp.
Neoscona_arabesca
Gasteracantha_hasselti
Macracantha_arcuata
Pararchea alba
Perissopmeros sp.
Malkaridae_sp.9
Malkaridae_sp.17
Ero_leonina
Australomimetus sp.
Tetragnatha_tantalus
Arkys sp.
Demadiana sp.
Meta_ovalis
Leucauge_venusta
Nanometa_sp
Nesticus_bishopi
Nesticus_cooperi
Physoglenes_sp_66294
4 Meringa_sp
Runga_sp
Pimoa sp.1
Pimoa_sp.2
Frontinella_pyramitela
Laminacauda parvipalpis
Novafroneta gladiatrix
Baalzebub_sp
Tekelloides_sp.2
Tekelloides_sp.1
Cepheia_longiseta
Spinanapis sp.
Novanapis sp.
Mysmena_leichardti
Mysmenella_jobi
Microdipoena_guttata
Tamopsis_sp
Menneus_sp
Deinopis_longipes
Deinopis_sp
Oecobius cellariorum
Oecobius_navus
Zosis sp.
Philoponella_herediae
Uloborus glomosus
Forsterella sp.
Cybaeodamus taim
Caayguara_ybityriguara
Amaurobius_ferox
Callobius_sp
Cycloctenus_sp
Neoramia_sp
Metaltella_simoni
Amphinecta_sp
Paramatachia_sp
Cambridgea_sp
Agelenopsis_emertoni
Agelenopsis_pennsylvanica
Calymmaria_sp
Calymmaria_persica
Cicurina_travisae
Cicurina_vobora
Homalonychus theologus
Uliodon sp.
Tengella radiata
Anahita punctulata
Senoculus sp.
Peucetia longipalpis
Misumenoides_formosipes
Sidymella_sp
Dolomedes triton
Pisaurina mira
Cupiennius coccineus
Allocosa_alticeps
Schizocosa_rovneri
Habronattus signatus
Habronattus_ustulatus
Nyssus sp.
Falconina gracilis
Karaops raveni
Teminius sp.
Rebilus_sp
Clubiona_sp
Liocranidae sp.
Trachelas tranquillus
Sergiolus capulatus
Molycria sp.
Anzacia sp.
Lampona sp.
0.2

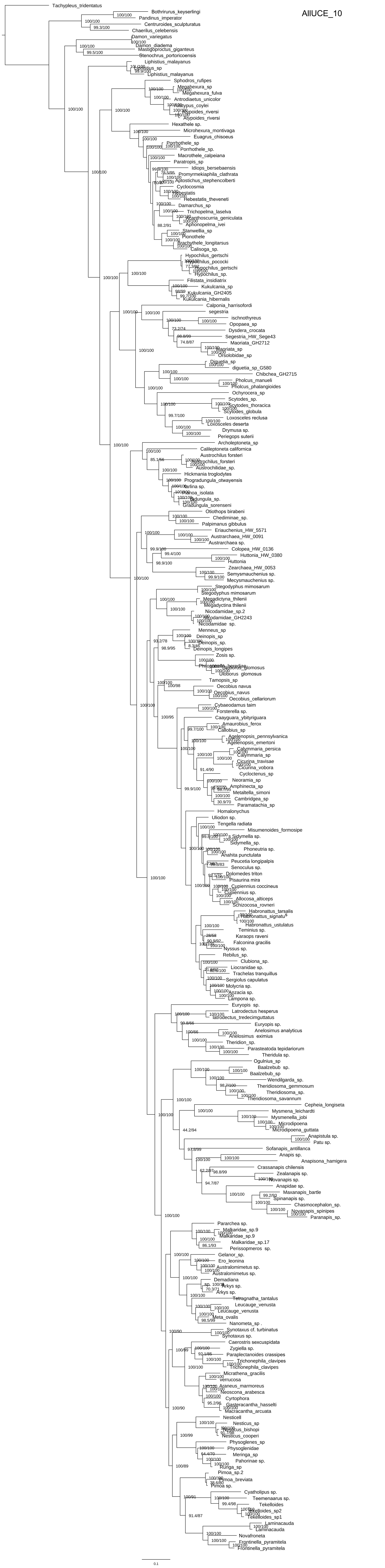
AllUCE_10
0.1

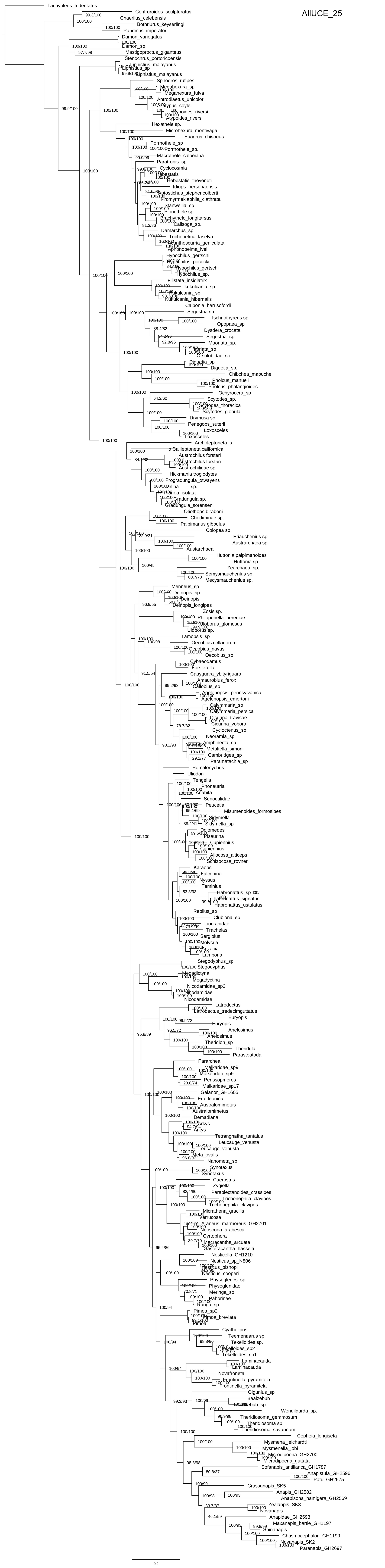
AllUCE_25
Tachypleus_tridentatus
Centruroides_sculpturatus
Chaerilus_celebensis
Bothriurus_keyserlingi
Pandinus_imperator
Damon_variegatus
Damon_sp
Mastigoproctus_giganteus
Stenochrus_portoricensis
Liphistius_malayanus
Liphistius_sp
Liphistius_malayanus
Sphodros_rufipes
Megahexura_sp
Megahexura_fulva
Antrodiaetus_unicolor
Atypus_coylei
Atypoides_riversi
Atypoides_riversi
Hexathele sp.
Microhexura_montivaga
Euagrus_chisoseus
Porrhothele_sp
Porrhothele_sp.
Macrothele_calpeiana
Paratropis_sp
Cyclocosmia
Hebestatis
Hebestatis_theveneti
Idiops_bersebaensis
Aptostichus_stephencolberti
Promyrmekiaphila_clathrata
Stanwellia_sp
Pionothele sp.
Brachythele_longitarsus
Calisoga_sp.
Damarchus_sp
Trichopelma_laselva
Acanthoscurria_geniculata
Aphonopelma_iviei
Hypochilus_gertschi
Hypochilus_pococki
Hypochilus_gertschi
Hypochilus_sp.
Filistata_insidiatrix
kukulcania_sp.
Kukulcania_sp.
Kukulcania_hibernalis
Calponia_harrisofordi
Segestria sp.
Ischnothyreus sp.
Opopaea_sp
Dysdera_crocata
Segestria_sp.
Maoriata_sp.
Ariadna_sp
Orsolobidae_sp
Diguetia_sp
Diguetia_sp.
Chibchea_mapuche
Pholcus_manueli
Pholcus_phalangioides
Ochyrocera_sp
Scytodes_sp.
Scytodes_thoracica
Scytodes_globula
Drymusa sp.
Periegops_suterii
Loxosceles
Loxosceles
Archoleptoneta_s
Calileptoneta californica
Austrochilus forsteri
Austrochilus forsteri
Austrochilidae sp.
Hickmania troglodytes
Progradungula_otwayens
Tarlina sp.
Kaitoa_isolata
Gradungula sp.
Gradungula_sorenseni
Otiothops birabeni
Chediminae sp.
Palpimanus gibbulus
Colopea sp.
Eriauchenius sp.
Austrarchaea sp.
Austarchaea
Huttonia palpimanoides
Huttonia sp.
Zearchaea sp.
Semysmauchenius sp.
Mecysmauchenius sp.
Menneus_sp
Deinopis_sp
Deinopis
Deinopis_longipes
Zosis sp.
Philoponella_herediae
Uloborus_glomosus
Uloborus sp.
Tamopsis_sp
Oecobius cellariorum
Oecobius_navus
Oecobius_sp
Cybaeodamus
Forsterella
Caayguara_ybityriguara
Amaurobius_ferox
Callobius_sp.
Agelenopsis_pennsylvanica
Agelenopsis_emertoni
Calymmaria_sp
Calymmaria_persica
Cicurina_travisae
Cicurina_vobora
Cycloctenus_sp
Neoramia_sp
Amphinecta_sp
Metaltella_simoni
Cambridgea_sp
Paramatachia_sp
Homalonychus
Uliodon
Tengella
Phoneutria
Anahita
Senoculidae
Peucetia
Misumenoides_formosipes
Sidymella
Sidymella_sp
Dolomedes
Pisaurina
Cupiennius
Cupiennius
Allocosa_alticeps
Schizocosa_rovneri
Karaops
Falconina
Nyssus
Teminius
Habronattus_sp
Habronattus_signatus
Habronattus_ustulatus
Rebilus_sp
Clubiona_sp
Liocranidae
Trachelas
Sergiolus
Molycria
Anzacia
Lampona
Stegodyphus_sp
Stegodyphus
Megadictyna
Megadyctina
Nicodamidae_sp2
Nicodamidae
Nicodamidae
Latrodectus
Latrodectus_tredecimguttatus
Euryopis
Euryopis
Anelosimus
Anelosimus
Theridion_sp
Theridula
Parasteatoda
Pararchaea
Malkaridae_sp9
Malkaridae_sp9
Perissopmeros
Malkaridae_sp17
Gelanor_GH1605
Ero_leonina
Australomimetus
Australomimetus
Demadiana
Arkys
Arkys
Tetragnatha_tantalus
Leucauge_venusta
Leucauge_venusta
Meta_ovalis
Nanometa_sp
Synotaxus
Synotaxus
Caerostris
Zygiella
Paraplectanoides_crassipes
Trichonephila_clavipes
Trichonephila_clavipes
Micrathena_gracilis
Verrucosa
Araneus_marmoreus_GH2701
Neoscona_arabesca
Cyrtophora
Macracantha_arcuata
Gasteracantha_hasselti
Nesticella_GH1210
Nesticus_sp_N806
Nesticus_bishopi
Nesticus_cooperi
Physoglenes_sp
Physoglenidae
Meringa_sp
Pahorinae
Runga_sp
Pimoa_sp2
Pimoa_breviata
Pimoa
Cyatholipus
Teemenaarus sp.
Tekelloides sp.
Tekelloides_sp2
Tekelloides_sp1
Laminacauda
Laminacauda
Novafroneta
Frontinella_pyramitela
Frontinella_pyramitela
Olgunius_sp
Baalzebub
Baalzebub_sp
Wendilgarda_sp.
Theridiosoma_gemmosum
Theridiosoma sp.
Theridiosoma_savannum
Cepheia_longiseta
Mysmena_leichardti
Mysmenella_jobi
Microdipoena_GH2700
Microdipoena_guttata
Sofanapis_antillanca_GH1787
Anapistula_GH2596
Patu_GH2575
Crassanapis_SK5
Anapis_GH2582
Anapisona_hamigera_GH2569
Zealanpis_SK3
Novanapis
Anapidae_GH2593
Maxanapis_bartle_GH1197
Spinanapis
Chasmocephalon_GH1199
Novanapis_SK2
Paranapis_GH2697
0.2

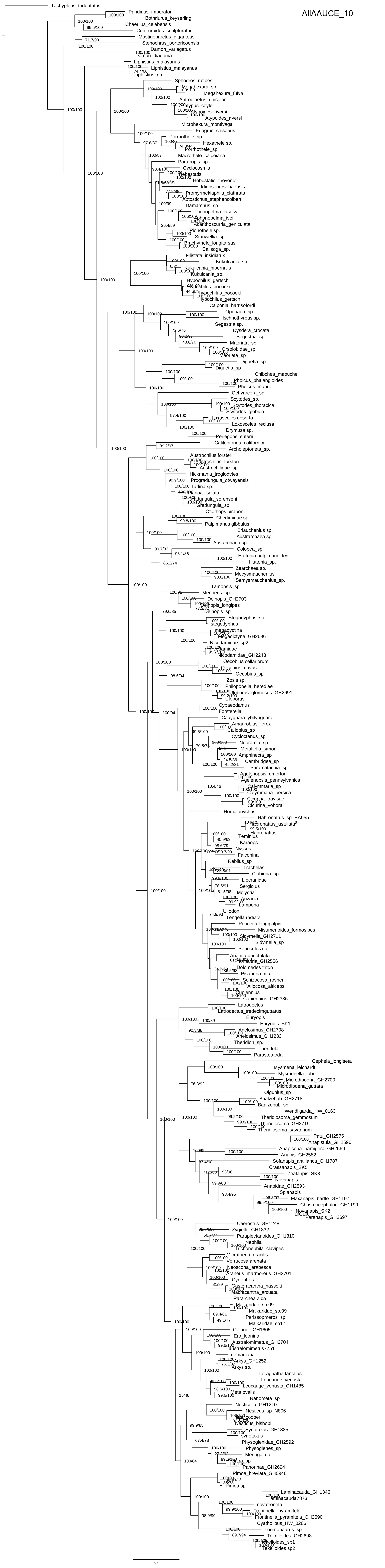
AllAAUCE_10
Tachypleus_tridentatus
Pandinus_imperator
Bothriurus_keyserlingi
Chaerilus_celebensis
Centruroides_sculpturatus
Mastigoproctus_giganteus
Stenochrus_portoricensis
Damon_variegatus
Damon_diadema
Liphistius_malayanus
Liphistius_sp
Sphodros_rufipes
Megahexura_sp
Megahexura_fulva
Antrodiaetus_unicolor
Aptypus_coylei
Atypoides_riversi
Microhexura_montivaga
Euagrus_chisoeus
Porrhothele_sp
Hexathele sp.
Porrhothele_sp.
Macrothele_calpeiana
Paratropis_sp
Cyclocosmia
Hebestatis
Hebestatis_theveneti
Idiops_bersebaensis
Promyrmekiaphila_clathrata
Aptostichus_stephencolberti
Damarchus_sp
Trichopelma_laselva
Aphonopelma_ivei
Acanthoscurria_geniculata
Pionothele sp.
Stanwellia_sp
Brachythele_longitarsus
Calisoga_sp.
Filistata_insidiatrix
Kukulcania_sp.
Kukulcania_hibernalis
Hypochilus_gertschi
Hypochilus_pococki
Calponia_harrisofordi
Opopaea_sp
Ischnothyreus sp.
Segestria sp.
Dysdera_crocata
Segestria_sp.
Maoriata_sp.
Orsolobidae_sp
Diguetia_sp.
Chibchea_mapuche
Pholcus_phalangioides
Pholcus_manueli
Ochyrocera_sp
Scytodes_sp.
Scytodes_thoracica
Scytodes_globula
Loxosceles deserta
Loxosceles reclusa
Drymusa sp.
Periegops_suterii
Calleptoneta californica
Archoleptoneta_sp.
Austrochilus forsteri
Austrochilidae_sp.
Hickmania_troglodytes
Progradungula_otwayensis
Tarlina sp.
Pianoa_isolata
Gradungula_sorenseni
Otiothops birabeni
Chediminae sp.
Palpimanus gibbulus
Eriauchenius sp.
Austrarchaea sp.
Colopea_sp.
Huttonia palpimanoides
Huttonia_sp.
Zearchaea sp.
Mecysmauchenius
Semysmauchenius_sp.
Tamopsis_sp
Menneus_sp
Deinopis_GH2703
Deinopis_longipes
Deinopis_sp
Stegodyphus_sp
stegodyphus
megadyctina
Megadictyna_GH2696
Nicodamidae_sp2
Nicodamidae
Nicodamidae_GH2243
Oecobius cellariorum
Oecobius_navus
Oecobius_sp
Zosis sp.
Philoponella_herediae
Uloborus_glomosus_GH2691
Uloborus
Cybaeodamus
Forsterella
Caayguara_ybityriguara
Amaurobius_ferox
Callobius_sp
Cycloctenus_sp
Neoramia_sp
Metaltella_simoni
Amphinecta_sp
Cambridgea_sp
Paramatachia_sp
Agelenopsis_emertoni
Agelenopsis_pennsylvanica
Calymmaria_sp
Calymmaria_persica
Cicurina_travisae
Cicurina_vobora
Homalonychus
Habronattus_sp_HA955
Habronattus_ustulatus
Habronattus
Teminius
Karaops
Nyssus
Falconina
Rebilus_sp
Trachelas
Clubiona_sp
Liocranidae
Sergiolus
Molycria
Anzacia
Lampona
Uliodon
Tengella radiata
Peucetia longipalpis
Misumenoides_formosipes
Sidymella_GH2711
Sidymella_sp
Senoculus sp.
Anahita punctulata
Nilus GH2556
Dolomedes triton
Pisaurina mira
Schizocosa_rovneri
Allocosa_alticeps
Cupiennius
Cupiennius_GH2386
Latrodectus
Latrodectus_tredecimguttatus
Euryopis
Euryopis_SK1
Anelosimus_GH2708
Anelosimus_GH1233
Theridion_sp.
Theridula
Parasteatoda
Cepheia_longiseta
Mysmena_leichardti
Mysmenella_jobi
Microdipoena_GH2700
Microdipoena_guttata
Olgunius_sp
Baalzebub_GH2718
Baalzebub_sp
Wendilgarda_HW_0163
Theridiosoma_gemmosum
Theridiosoma_GH2719
Theridiosoma_savannum
Patu_GH2575
Anapistula_GH2596
Anapisona_hamigera_GH2569
Anapis_GH2582
Sofanapis_antillanca_GH1787
Crassanapis_SK5
Zealanpis_SK3
Novanapis
Anapidae_GH2593
Spinanapis
Maxanapis_bartle_GH1197
Chasmocephalon_GH1199
Novanapis_SK2
Paranapis_GH2697
Caerostris_GH1248
Zygiella_GH1832
Paraplectanoides_GH1810
Nephila
Trichonephila_clavipes
Micrathena_gracilis
Verrucosa arenata
Neoscona_arabesca
Araneus_marmoreus_GH2701
Cyrtophora
Gasteracantha_hasselti
Macracantha_arcuata
Pararchaea alba
Malkaridae_sp.09
Malkaridae_sp.09
Perissopmeros sp.
Malkaridae_sp17
Gelanor_GH1605
Ero_leonina
Australomimetus_GH2704
australomimetus7751
demadiana
Arkys_GH1252
Arkys sp.
Tetragnatha tantalus
Leucauge_venusta
Leucauge_venusta_GH1485
Meta ovalis
Nanometa_sp
Nesticella_GH1210
Nesticus_sp_N806
Nesticus cooperi
Nesticus_bishopi
Synotaxus_GH1385
synotaxus
Physoglenidae_GH2592
Physoglenes_sp
Meringa_sp
Pahorinae_GH2694
Pimoa_breviata_GH0946
Pimoa sp.
Laminacauda_GH1346
laminacauda7873
novafroneta
Frontinella_pyramitela
Frontinella_pyramitela_GH2690
Cyatholipus_HW_0266
Teemenaarus_sp.
Tekelloides_GH2698
Tekelloides_sp1
Tekelloides_sp2
0.2

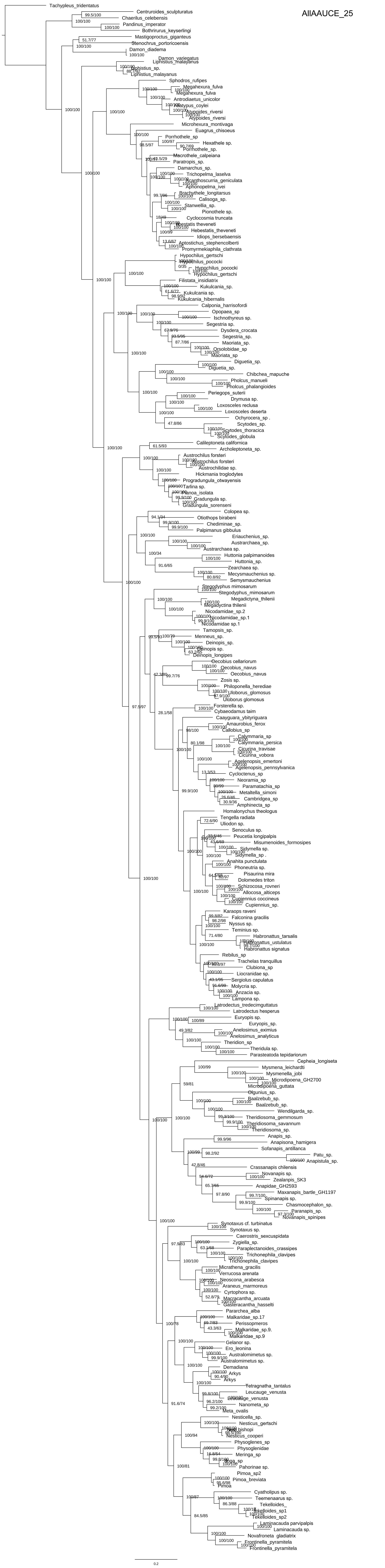

AllAAUCE_25
Tachypleus_tridentatus
Centruroides_sculpturatus
Chaerilus_celebensis
Pandinus_imperator
Bothriurus_keyserlingi
Mastigoproctus_giganteus
Stenochrus_portoricoensis
Damon_diadema
Damon_variegatus
Liphistius_malayanus
Liphistius_sp.
Liphistius_malayanus
Sphodros_rufipes
Megahexura_fulva
Megahexura_fulva
Antrodiaetus_unicolor
Atypus_coylei
Atypoides_riversi
Atypoides_riversi
Microhexura_montivaga
Euagrus_chisoseus
Porrhothele_sp
Hexathele sp.
Porrhothele_sp.
Macrothele_calpeiana
Paratropis_sp.
Damarchus_sp.
Trichopelma_laselva
Acanthoscurria_geniculata
Aphonopelma_iviei
Brachythele_longitarsus
Calisoga_sp.
Stanwellia_sp.
Pionothele sp.
Cyclocosmia truncata
Hebestatis theveneti
Hebestatis_theveneti
Idiops_bersebaensis
Aptostichus_stephencolberti
Promyrmekiaphila_clathrata
Hypochilus_gertschi
Hypochilus_pococki
Hypochilus_pococki
Hypochilus_gertschi
Filistata_insidiatrix
Kukulcania_sp.
Kukulcania sp.
Kukulcania_hibernalis
Calponia_harrisofordi
Opopaea_sp
Ischnothyreus sp.
Segestria sp.
Dysdera_crocata
Segestria_sp.
Maoriata_sp.
Orsolobidae_sp
Maoriata_sp
Diguetia_sp.
Diguetia_sp.
Chibchea_mapuche
Pholcus_manueli
Pholcus_phalangioides
Periegops_suterii
Drymusa sp.
Loxosceles reclusa
Loxosceles deserta
Ochyrocera_sp .
Scytodes_sp.
Scytodes_thoracica
Scytodes_globula
Calileptoneta californica
Archoleptoneta_sp.
Austrochilus forsteri
Austrochilus forsteri
Austrochilidae sp.
Hickmania troglodytes
Progradungula_otwayensis
Tarlina sp.
Hypochilus_pococki
Gradungula sp.
Gradungula_sorenseni
Colopea sp.
Otiothops birabeni
Chediminae_sp.
Palpimanus gibbulus
Eriauchenius_sp.
Austrarchaea_sp.
Austrarchaea sp.
Huttonia palpimanoides
Huttonia_sp.
Zearchaea sp.
Mecysmauchenius sp.
Semysmauchenius
Stegodyphus mimosarum
Stegodyphus_mimosarum
Megadictyna_thilenii
Megadictyna thilenii
Nicodamidae_sp.2
Nicodamidae_sp.1
Nicodamidae sp.1
Tamopsis_sp.
Menneus_sp.
Deinopis_sp.
Deinopis sp.
Deinopis_longipes
Oecobius cellariorum
Oecobius_navus
Oecobius_navus
Zosis sp.
Philoponella_herediae
Uloborus_glomosus
Uloborus glomosus
Forsterella sp.
Cybaeodamus taim
Caayguara_ybityriguara
Amaurobius_ferox
Callobius_sp
Calymmaria_sp
Calymmaria_persica
Cicurina_travisae
Cicurina_vobora
Agelenopsis_emertoni
Agelenopsis_pennsylvanica
Cycloctenus_sp
Neoramia_sp
Paramatachia_sp
Metaltella_simoni
Cambridgea_sp
Amphinecta_sp
Homalonychus theologus
Tengella radiata
Uliodon sp.
Senoculus sp.
Peucetia longipalpis
Misumenoides_formosipes
Sidymella sp.
Sidymella_sp .
Anahita punctulata
Phoneutria sp.
Pisaurina mira
Dolomedes triton
Schizocosa_rovneri
Allocosa_alticeps
Cupiennius coccineus
Cupiennius_sp.
Karaops raveni
Falconina gracilis
Nyssus sp.
Teminius sp.
Habronattus_tarsalis
Habronattus_ustulatus
Habronattus signatus
Rebilus_sp
Trachelas tranquillus
Clubiona_sp
Liocranidae sp.
Sergiolus capulatus
Molycria sp.
Anzacia sp.
Lampona sp.
Latrodectus_tredecimguttatus
Latrodectus hesperus
Euryopis sp.
Euryopis_sp.
Anelosimus_eximius
Anelosimus_analyticus
Theridion_sp
Theridula sp.
Parasteatoda tepidariorum
Cepheia_longiseta
Mysmena_leichardti
Mysmenella_jobi
Microdipoena_GH2700
Microdipoena_guttata
Ogulnius_sp.
Baalzebub_sp.
Baalzebub_sp.
Wendilgarda_sp.
Theridiosoma_gemmosum
Theridiosoma_savannum
Theridiosoma_sp.
Anapis_sp.
Anapisona_hamigera
Sofanapis_antillanca
Patu_sp.
Anapistula_sp.
Crassanapis chilensis
Novanapis sp.
Zealanapis_SK3
Anapidae_GH2593
Maxanapis_bartle_GH1197
Spinanapis sp.
Chasmocephalon_sp.
Paranapis_sp.
Novanapis_spinipes
Synotaxus cf. turbinatus
Synotaxus sp.
Caerostris_sexcuspidata
Zygiella_sp.
Paraplectanoides_crassipes
Trichonephila_clavipes
Trichonephila_clavipes
Micrathena_gracilis
Verrucosa arenata
Neoscona_arabesca
Araneus_marmoreus
Cyrtophora sp.
Macracantha_arcuata
Gasteracantha_hasselti
Pararchaea_alba
Malkaridae_sp.17
Perissopmeros
Malkaridae_sp.9
Malkaridae_sp.9
Gelanor sp.
Ero_leonina
Australomimetus sp.
Australomimetus sp.
Demadiana
Arkys
Arkys
Tetragnatha_tantalus
Leucauge_venusta
Leucauge_venusta
Nanometa_sp
Meta_ovalis
Nesticella_sp.
Nesticus_gertschi
Nesticus_bishopi
Nesticus_cooperi
Physoglenes_sp
Physoglenidae
Meringa_sp
Pahorinae sp.
Pimoa_sp2
Pimoa_breviata
Pimoa
Cyatholipus sp.
Teemenaarus sp.
Tekelloides_
Tekelloides_sp1
Tekelloides_sp2
Laminacauda parvipalpis
Laminacauda sp.
Novafroneta_gladiatrix
Frontinella_pyramitela
Frontinella_pyramitela
0.2

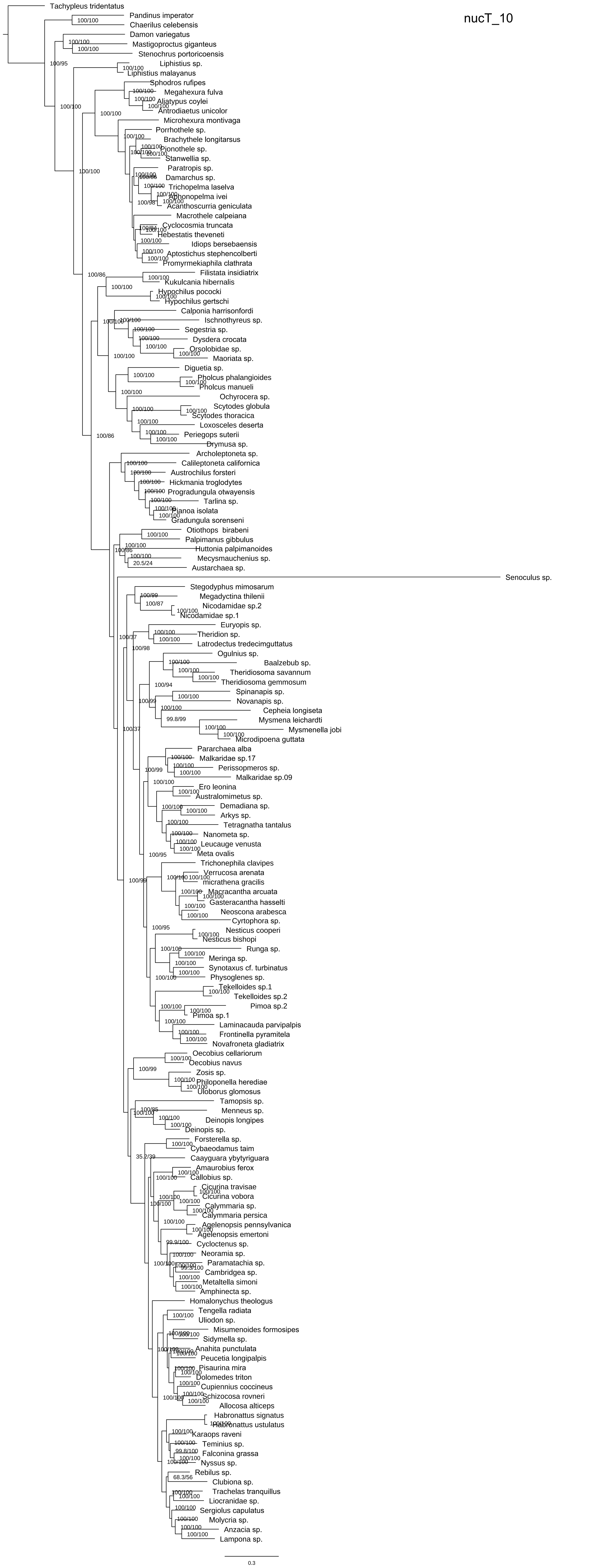
nucT_10
Tachypleus tridentatus
Pandinus imperator
Chaerilus celebensis
Damon variegatus
Mastigoproctus giganteus
Stenochrus portoricoensis
Liphistius sp.
Liphistius malayanus
Sphodros rufipes
Megahexura fulva
Aliatypus coylei
Antrodiaetus unicolor
Microhexura montivaga
Porrhothele sp.
Brachythele longitarsus
Pionothele sp.
Stanwellia sp.
Paratropis sp.
Damarchus sp.
Trichopelma laselva
Aphonopelma ivei
Acanthoscurria geniculata
Macrothele calpeiana
Cyclocosmia truncata
Hebestatis theveneti
Idiops bersebaensis
Aptostichus stephencolberti
Promyrmekiaphila clathrata
Filistata insidiatrix
Kukulcania hibernalis
Hypochilus pococki
Hypochilus gertschi
Calponia harrisonfordi
Ischnothyreus sp.
Segestria sp.
Dysdera crocata
Orsolobidae sp.
Maoriata sp.
Diguetia sp.
Pholcus phalangioides
Pholcus manueli
Ochyrocera sp.
Scytodes globula
Scytodes thoracica
Loxosceles deserta
Periegops suterii
Drymusa sp.
Archoleptoneta sp.
Calileptoneta californica
Austrochilus forsteri
Hickmania troglodytes
Progradungula otwayensis
Tarlina sp.
Pianoa isolata
Gradungula sorenseni
Otiothops birabeni
Palpimanus gibbulus
Huttonia palpimanoides
Mecysmauchenius sp.
Austarchaea sp.
Senoculus sp.
Stegodyphus mimosarum
Megadyctina thilenii
Nicodamidae sp.2
Nicodamidae sp.1
Euryopis sp.
Theridion sp.
Latrodectus tredecimguttatus
Ogulnius sp.
Baalzebub sp.
Theridiosoma savannum
Theridiosoma gemmosum
Spinanapis sp.
Novanapis sp.
Cepheia longiseta
Mysmena leichardti
Mysmenella jobi
Microdipoena guttata
Pararchaea alba
Malkaridae sp.17
Perissopmeros sp.
Malkaridae sp.09
Ero leonina
Australomimetus sp.
Demadiana sp.
Arkys sp.
Tetragnatha tantalus
Nanometa sp.
Leucauge venusta
Meta ovalis
Trichonephila clavipes
Verrucosa arenata
micrathena gracilis
Macracantha arcuata
Gasteracantha hasselti
Neoscona arabesca
Cyrtophora sp.
Nesticus cooperi
Nesticus bishopi
Runga sp.
Meringa sp.
Synotaxus cf. turbinatus
Physoglenes sp.
Tekelloides sp.1
Tekelloides sp.2
Pimoa sp.2
Pimoa sp.1
Laminacauda parvipalpis
Frontinella pyramitela
Novafroneta gladiatrix
Oecobius cellariorum
Oecobius navus
Zosis sp.
Philoponella herediae
Uloborus glomosus
Tamopsis sp.
Menneus sp.
Deinopis longipes
Deinopis sp.
Forsterella sp.
Cybaeodamus taim
Caayguara ybytyriguara
Amaurobius ferox
Callobius sp.
Cicurina travisae
Cicurina vobora
Calymmaria sp.
Calymmaria persica
Agelenopsis pennsylvanica
Agelenopsis emertoni
Cycloctenus sp.
Neoramia sp.
Paramatachia sp.
Cambridgea sp.
Metaltella simoni
Amphinecta sp.
Homalonychus theologus
Tengella radiata
Uliodon sp.
Misumenoides formosipes
Sidymella sp.
Anahita punctulata
Peucetia longipalpis
Pisaurina mira
Dolomedes triton
Cupiennius coccineus
Schizocosa rovneri
Allocosa alticeps
Habronattus signatus
Habronattus ustulatus
Karaops raveni
Teminius sp.
Falconina grassa
Nyssus sp.
Rebilus sp.
Clubiona sp.
Trachelas tranquillus
Liocranidae sp.
Sergiolus capulatus
Molycria sp.
Anzacia sp.
Lampona sp.
0.3

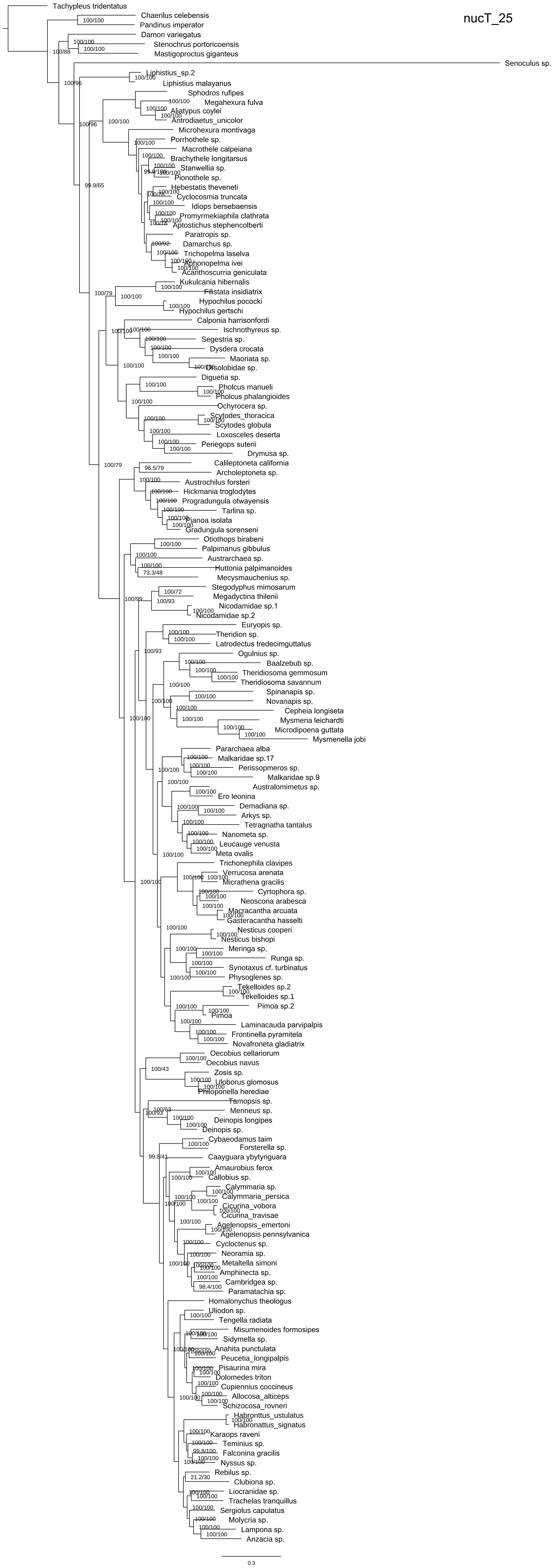

nucT_25
Tachypleus tridentatus
Chaerilus celebensis
Pandinus imperator
Damon variegatus
Stenochrus portoricoensis
Mastigoproctus giganteus
Senoculus sp.
Liphistius_sp.2
Liphistius malayanus
Sphodros rufipes
Megahexura fulva
Aliatypus coylei
Antrodiaetus_unicolor
Microhexura montivaga
Porrhothele sp.
Macrothele calpeiana
Brachythele longitarsus
Stanwellia sp.
Pionothele sp.
Hebestatis theveneti
Cyclocosmia truncata
Idiops bersebaensis
Promyrmekiaphila clathrata
Aptostichus stephencolberti
Paratropis sp.
Damarchus sp.
Trichopelma laselva
Aphonopelma ivei
Acanthoscurria geniculata
Kukulcania hibernalis
Filistata insidiatrix
Hypochilus pococki
Hypochilus gertschi
Calponia harrisonfordi
Ischnothyreus sp.
Segestria sp.
Dysdera crocata
Maoriata sp.
Orsolobidae sp.
Diguetia sp.
Pholcus manueli
Pholcus phalangioides
Ochyrocera sp.
Scytodes_thoracica
Scytodes globula
Loxosceles deserta
Periegops suterii
Drymusa sp.
Calileptoneta californica
Archoleptoneta sp.
Austrochilus forsteri
Hickmania troglodytes
Progradungula otwayensis
Tarlina sp.
Pianoa isolata
Gradungula sorenseni
Otiothops birabeni
Palpimanus gibbulus
Austrarchaea sp.
Huttonia palpimanoides
Mecysmauchenius sp.
Stegodyphus mimosarum
Megadyctina thilenii
Nicodamidae sp.1
Nicodamidae sp.2
Euryopis sp.
Theridion sp.
Latrodectus tredecimguttatus
Ogulnius sp.
Baalzebub sp.
Theridiosoma gemmosum
Theridiosoma savannum
Spinanapis sp.
Novanapis sp.
Cepheia longiseta
Mysmena leichardti
Microdipoena guttata
Mysmenella jobi
Pararchaea alba
Malkaridae sp.17
Perissopmeros sp.
Malkaridae sp.9
Australomimetus sp.
Ero leonina
Demadiana sp.
Arkys sp.
Tetragnatha tantalus
Nanometa sp.
Leucauge venusta
Meta ovalis
Trichonephila clavipes
Verrucosa arenata
Micrathena gracilis
Cyrtophora sp.
Neoscona arabesca
Macracantha arcuata
Gasteracantha hasselti
Nesticus cooperi
Nesticus bishopi
Meringa sp.
Runga sp.
Synotaxus cf. turbinatus
Physoglenes sp.
Tekelloides sp.2
Tekelloides sp.1
Pimoa sp.2
Pimoa
Laminacauda parvipalpis
Frontinella pyramitela
Novafroneta gladiatrix
Oecobius cellariorum
Oecobius navus
Zosis sp.
Uloborus glomosus
Philoponella herediae
Tamopsis sp.
Menneus sp.
Deinopis longipes
Deinopis sp.
Cybaeodamus taim
Forsterella sp.
Caayguara ybytyriguara
Amaurobius ferox
Callobius sp.
Calymmaria sp.
Calymmaria_persica
Cicurina_vobora
Cicurina_travisae
Agelenopsis_emertoni
Agelenopsis pennsylvanica
Cycloctenus sp.
Neoramia sp.
Metaltella simoni
Amphinecta sp.
Cambridgea sp.
Paramatachia sp.
Homalonychus theologus
Uliodon sp.
Tengella radiata
Misumenoides formosipes
Sidymella sp.
Anahita punctulata
Peucetia_longipalpis
Pisaurina mira
Dolomedes triton
Cupiennius coccineus
Allocosa_alticeps
Schizocosa_rovneri
Habronttus_ustulatus
Habronattus_signatus
Karaops raveni
Teminius sp.
Falconina gracilis
Nyssus sp.
Rebilus sp.
Clubiona sp.
Liocranidae sp.
Trachelas tranquillus
Sergiolus capulatus
Molycria sp.
Lampona sp.
Anzacia sp.
0.3

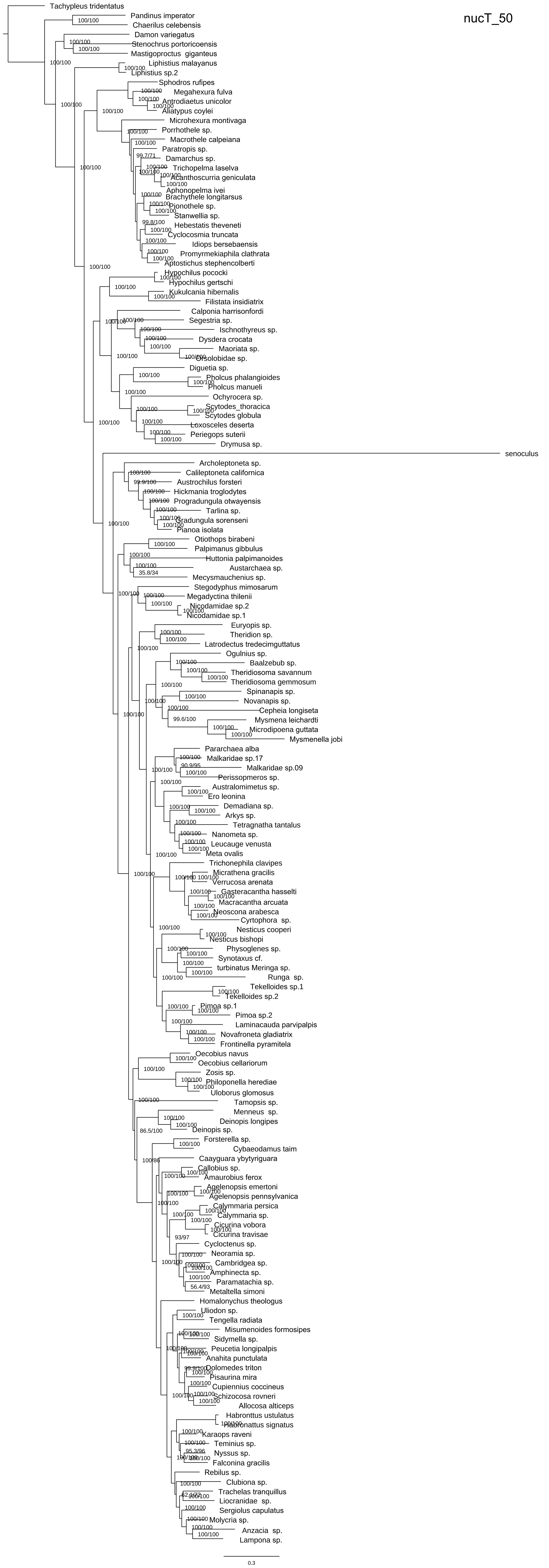

nucT_50
Tachypleus tridentatus
Pandinus imperator
Chaerilus celebensis
Damon variegatus
Stenochrus portoricoensis
Mastigoproctus giganteus
Liphistius malayanus
Liphistius sp.2
Sphodros rufipes
Megahexura fulva
Antrodiaetus unicolor
Aliatypus coylei
Microhexura montivaga
Porrhothele sp.
Macrothele calpeiana
Paratropis sp.
Damarchus sp.
Trichopelma laselva
Acanthoscurria geniculata
Aphonopelma ivei
Brachythele longitarsus
Pionothele sp.
Stanwellia sp.
Hebestatis theveneti
Cyclocosmia truncata
Idiops bersebaensis
Promyrmekiaphila clathrata
Aptostichus stephencolberti
Hypochilus pococki
Hypochilus gertschi
Kukulcania hibernalis
Filistata insidiatrix
Calponia harrisonfordi
Segestria sp.
Ischnothyreus sp.
Dysdera crocata
Maoriata sp.
Orsolobidae sp.
Diguetia sp.
Pholcus phalangioides
Pholcus manueli
Ochyrocera sp.
Scytodes_thoracica
Scytodes globula
Loxosceles deserta
Periegops suterii
Drymusa sp.
senoculus
Archoleptoneta sp.
Calileptoneta californica
Austrochilus forsteri
Hickmania troglodytes
Progradungula otwayensis
Tarlina sp.
Gradungula sorenseni
Pianoa isolata
Otiothops birabeni
Palpimanus gibbulus
Huttonia palpimanoides
Austarchaea sp.
Mecysmauchenius sp.
Stegodyphus mimosarum
Megadyctina thilenii
Nicodamidae sp.2
Nicodamidae sp.1
Euryopis sp.
Theridion sp.
Latrodectus tredecimguttatus
Ogulnius sp.
Baalzebub sp.
Theridiosoma savannum
Theridiosoma gemmosum
Spinanapis sp.
Novanapis sp.
Cepheia longiseta
Mysmena leichardti
Microdipoena guttata
Mysmenella jobi
Pararchaea alba
Malkaridae sp.17
Malkaridae sp.09
Perissopmeros sp.
Australomimetus sp.
Ero leonina
Demadiana sp.
Arkys sp.
Tetragnatha tantalus
Nanometa sp.
Leucauge venusta
Meta ovalis
Trichonephila clavipes
Micrathena gracilis
Verrucosa arenata
Gasteracantha hasselti
Macracantha arcuata
Neoscona arabesca
Cyrtophora sp.
Nesticus cooperi
Nesticus bishopi
Physoglenes sp.
Synotaxus cf.
turbinatus Meringa sp.
Runga sp.
Tekelloides sp.1
Tekelloides sp.2
Pimoa sp.1
Pimoa sp.2
Laminacauda parvipalpis
Novafroneta gladiatrix
Frontinella pyramitela
Oecobius navus
Oecobius cellariorum
Zosis sp.
Philoponella herediae
Uloborus glomosus
Tamopsis sp.
Menneus sp.
Deinopis longipes
Deinopis sp.
Forsterella sp.
Cybaeodamus taim
Caayguara ybytyriguara
Callobius sp.
Amaurobius ferox
Agelenopsis emertoni
Agelenopsis pennsylvanica
Calymmaria persica
Calymmaria sp.
Cicurina vobora
Cicurina travisae
Cycloctenus sp.
Neoramia sp.
Cambridgea sp.
Amphinecta sp.
Paramatachia sp.
Metaltella simoni
Homalonychus theologus
Uliodon sp.
Tengella radiata
Misumenoides formosipes
Sidymella sp.
Peucetia longipalpis
Anahita punctulata
Dolomedes triton
Pisaurina mira
Cupiennius coccineus
Schizocosa rovneri
Allocosa alticeps
Habronttus ustulatus
Habronattus signatus
Karaops raveni
Teminius sp.
Nyssus sp.
Falconina gracilis
Rebilus sp.
Clubiona sp.
Trachelas tranquillus
Liocranidae sp.
Sergiolus capulatus
Molycria sp.
Anzacia sp.
Lampona sp.
0.3

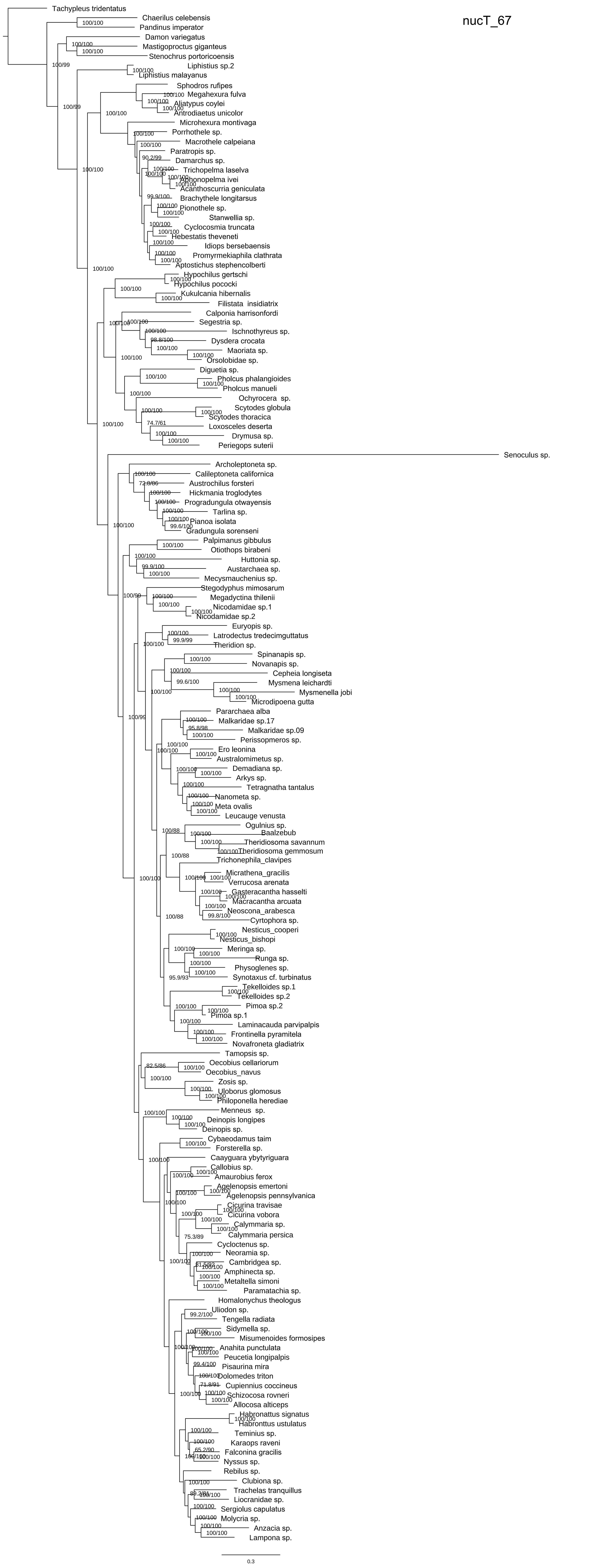
nucT_67
Tachypleus tridentatus
Chaerilus celebensis
Pandinus imperator
Damon variegatus
Mastigoproctus giganteus
Stenochrus portoricoensis
Liphistius sp.2
Liphistius malayanus
Sphodros rufipes
Megahexura fulva
Aliatypus coylei
Antrodiaetus unicolor
Microhexura montivaga
Porrhothele sp.
Macrothele calpeiana
Paratropis sp.
Damarchus sp.
Trichopelma laselva
Aphonopelma iviei
Acanthoscurria geniculata
Brachythele longitarsus
Pionothele sp.
Stanwellia sp.
Cyclocosmia truncata
Hebestatis theveneti
Idiops bersebaensis
Promyrmekiaphila clathrata
Aptostichus stephencolberti
Hypochilus gertschi
Hypochilus pococki
Kukulcania hibernalis
Filistata insidiatrix
Calponia harrisonfordi
Segestria sp.
Ischnothyreus sp.
Dysdera crocata
Maoriata sp.
Orsolobidae sp.
Diguetia sp.
Pholcus phalangioides
Pholcus manueli
Ochyrocera sp.
Scytodes globula
Scytodes thoracica
Loxosceles deserta
Drymusa sp.
Periegops suterii
Senoculus sp.
Archoleptoneta sp.
Calileptoneta californica
Austrochilus forsteri
Hickmania troglodytes
Progradungula otwayensis
Tarlina sp.
Pianoa isolata
Gradungula sorenseni
Palpimanus gibbulus
Otiothops birabeni
Huttonia sp.
Austarchaea sp.
Mecysmauchenius sp.
Stegodyphus mimosarum
Megadyctina thilenii
Nicodamidae sp.1
Nicodamidae sp.2
Euryopis sp.
Latrodectus tredecimguttatus
Theridion sp.
Spinanapis sp.
Novanapis sp.
Cepheia longiseta
Mysmena leichardti
Mysmenella jobi
Microdipoena gutta
Pararchaea alba
Malkaridae sp.17
Malkaridae sp.09
Perissopmeros sp.
Ero leonina
Australomimetus sp.
Demadiana sp.
Arkys sp.
Tetragnatha tantalus
Nanometa sp.
Meta ovalis
Leucauge venusta
Ogulnius sp.
Baalzebub
Theridiosoma savannum
Theridiosoma gemmosum
Trichonephila_clavipes
Micrathena_gracilis
Verrucosa arenata
Gasteracantha hasselti
Macracantha arcuata
Neoscona_arabesca
Cyrtophora sp.
Nesticus_cooperi
Nesticus_bishopi
Meringa sp.
Runga sp.
Physoglenes sp.
Synotaxus cf. turbinatus
Tekelloides sp.1
Tekelloides sp.2
Pimoa sp.2
Pimoa sp.1
Laminacauda parvipalpis
Frontinella pyramitela
Novafroneta gladiatrix
Tamopsis sp.
Oecobius cellariorum
Oecobius_navus
Zosis sp.
Uloborus glomosus
Philoponella herediae
Menneus sp.
Deinopis longipes
Deinopis sp.
Cybaeodamus taim
Forsterella sp.
Caayguara ybytyriguara
Callobius sp.
Amaurobius ferox
Agelenopsis emertoni
Agelenopsis pennsylvanica
Cicurina travisae
Cicurina vobora
Calymmaria sp.
Calymmaria persica
Cycloctenus sp.
Neoramia sp.
Cambridgea sp.
Amphinecta sp.
Metaltella simoni
Paramatachia sp.
Homalonychus theologus
Uliodon sp.
Tengella radiata
Sidymella sp.
Misumenoides formosipes
Anahita punctulata
Peucetia longipalpis
Pisaurina mira
Dolomedes triton
Cupiennius coccineus
Schizocosa rovneri
Allocosa alticeps
Habronattus signatus
Habronttus ustulatus
Teminius sp.
Karaops raveni
Falconina gracilis
Nyssus sp.
Rebilus sp.
Clubiona sp.
Trachelas tranquillus
Liocranidae sp.
Sergiolus capulatus
Molycria sp.
Anzacia sp.
Lampona sp.
0.3

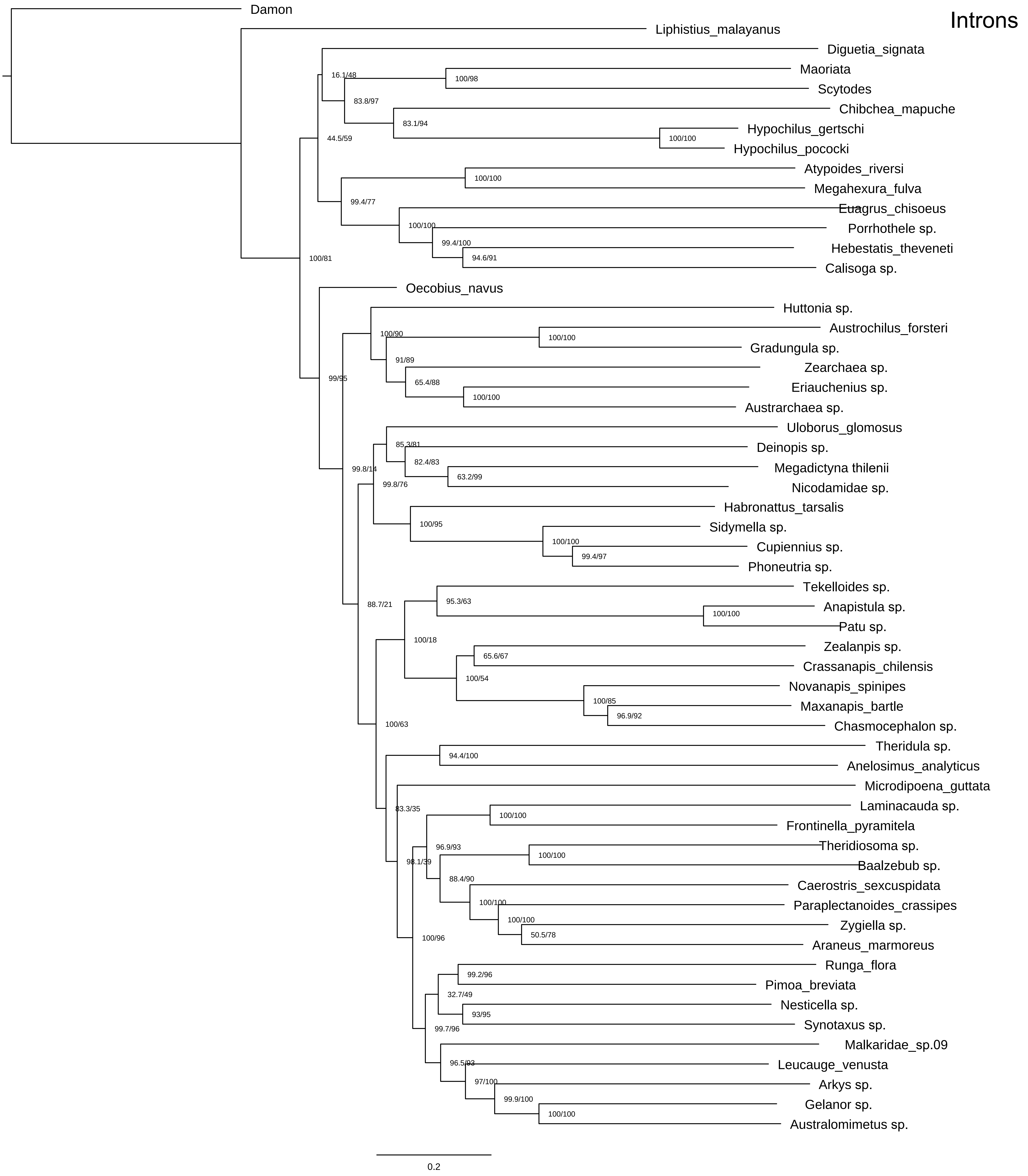
Introns
Damon
Liphistius_malayanus
Diguetia_signata
Maoriata
Scytodes
Chibchea_mapuche
Hypochilus_gertschi
Hypochilus_pococki
Atypoides_riversi
Megahexura_fulva
Euagrus_chisoeus
Porrhothele sp.
Hebestatis_theveneti
Calisoga sp.
Oecobius_navus
Huttonia sp.
Austrochilus_forsteri
Gradungula sp.
Zearchaea sp.
Eriauchenius sp.
Austrarchaea sp.
Uloborus_glomosus
Deinopis sp.
Megadictyna thilenii
Nicodamidae sp.
Habronattus_tarsalis
Sidymella sp.
Cupiennius sp.
Phoneutria sp.
Tekelloides sp.
Anapistula sp.
Patu sp.
Zealanpis sp.
Crassanapis_chilensis
Novanapis_spinipes
Maxanapis_bartle
Chasmocephalon sp.
Theridula sp.
Anelosimus_analyticus
Microdipoena_guttata
Laminacauda sp.
Frontinella_pyramitela
Theridiosoma sp.
Baalzebub sp.
Caerostris_sexcuspidata
Paraplectanoides_crassipes
Zygiella sp.
Araneus_marmoreus
Runga_flora
Pimoa_breviata
Nesticella sp.
Synotaxus sp.
Malkaridae_sp.09
Leucauge_venusta
Arkys sp.
Gelanor sp.
Australomimetus sp.
16.1/48
100/98
83.8/97
83.1/94
100/100
44.5/59
100/100
99.4/77
100/100
99.4/100
94.6/91
100/81
100/90
100/100
91/89
99/95
65.4/88
100/100
85.3/81
82.4/83
99.8/14
63.2/99
99.8/76
100/95
100/100
99.4/97
88.7/21
95.3/63
100/100
100/18
65.6/67
100/54
100/85
96.9/92
100/63
94.4/100
83.3/35
100/100
96.9/93
100/100
98.1/39
88.4/90
100/100
100/100
50.5/78
100/96
99.2/96
32.7/49
93/95
99.7/96
96.5/93
97/100
99.9/100
100/100
0.2

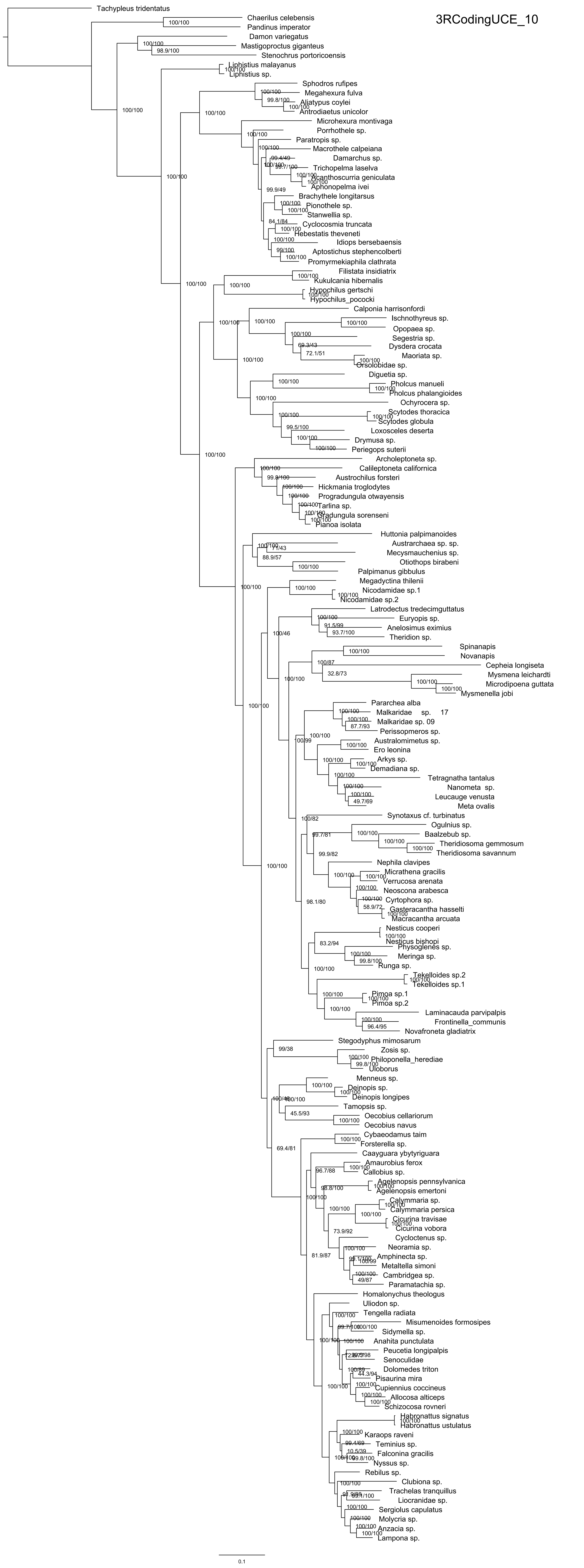
3RCodingUCE_10
Tachypleus tridentatus
Chaerilus celebensis
Pandinus imperator
Damon variegatus
Mastigoproctus giganteus
Stenochrus portoricoensis
Liphistius malayanus
Liphistius sp.
Sphodros rufipes
Megahexura fulva
Aliatypus coylei
Antrodiaetus unicolor
Microhexura montivaga
Porrhothele sp.
Paratropis sp.
Macrothele calpeiana
Damarchus sp.
Trichopelma laselva
Acanthoscurria geniculata
Aphonopelma iveі
Brachythele longitarsus
Pionothele sp.
Stanwellia sp.
Cyclocosmia truncata
Hebestatis theveneti
Idiops bersebaensis
Aptostichus stephencolberti
Promyrmekiaphila clathrata
Filistata insidiatrix
Kukulcania hibernalis
Hypochilus gertschi
Hypochilus_pococki
Calponia harrisonfordi
Ischnothyreus sp.
Opopaea sp.
Segestria sp.
Dysdera crocata
Maoriata sp.
Orsolobidae sp.
Diguetia sp.
Pholcus manueli
Pholcus phalangioides
Ochyrocera sp.
Scytodes thoracica
Scytodes globula
Loxosceles deserta
Drymusa sp.
Periegops suteri
Archoleptoneta sp.
Calileptoneta californica
Austrochilus forsteri
Hickmania troglodytes
Progradungula otwayensis
Tarlina sp.
Gradungula sorenseni
Pianoa isolata
Huttonia palpimanoides
Austrarchaea sp. sp.
Mecysmauchenius sp.
Otiothops birabeni
Palpimanus gibbulus
Megadyctina thilenii
Nicodamidae sp.1
Nicodamidae sp.2
Latrodectus tredecimguttatus
Euryopis sp.
Anelosimus eximius
Theridion sp.
Spinanapis
Novanapis
Cepheia longiseta
Mysmena leichardti
Microdipoena guttata
Mysmenella jobi
Pararchea alba
Malkaridae sp. 17
Malkaridae sp. 09
Perissopmeros sp.
Australomimetus sp.
Ero leonina
Arkys sp.
Demadiana sp.
Tetragnatha tantalus
Nanometa sp.
Leucauge venusta
Meta ovalis
Synotaxus cf. turbinatus
Ogulnius sp.
Baalzebub sp.
Theridiosoma gemmosum
Theridiosoma savannum
Nephila clavipes
Micrathena gracilis
Verrucosa arenata
Neoscona arabesca
Cyrtophora sp.
Gasteracantha hasselti
Macracantha arcuata
Nesticus cooperi
Nesticus bishopi
Physoglenes sp.
Meringa sp.
Runga sp.
Tekelloides sp.2
Tekelloides sp.1
Pimoa sp.1
Pimoa sp.2
Laminacauda parvipalpis
Frontinella_communis
Novafroneta gladiatrix
Stegodyphus mimosarum
Zosis sp.
Philoponella_herediae
Uloborus
Menneus sp.
Deinopis sp.
Deinopis longipes
Tamopsis sp.
Oecobius cellariorum
Oecobius navus
Cybaeodamus taim
Forsterella sp.
Caayguara ybytyriguara
Amaurobius ferox
Callobius sp.
Agelenopsis pennsylvanica
Agelenopsis emertoni
Calymmaria sp.
Calymmaria persica
Cicurina travisae
Cicurina vobora
Cycloctenus sp.
Neoramia sp.
Amphinecta sp.
Metaltella simoni
Cambridgea sp.
Paramatachia sp.
Homalonychus theologus
Uliodon sp.
Tengella radiata
Misumenoides formosipes
Sidymella sp.
Anahita punctulata
Peucetia longipalpis
Senoculidae
Dolomedes triton
Pisaurina mira
Cupiennius coccineus
Allocosa alticeps
Schizocosa rovneri
Habronattus signatus
Habronattus ustulatus
Karaops raveni
Teminius sp.
Falconina gracilis
Nyssus sp.
Rebilus sp.
Clubiona sp.
Trachelas tranquillus
Liocranidae sp.
Sergiolus capulatus
Molycria sp.
Anzacia sp.
Lampona sp.
0.1

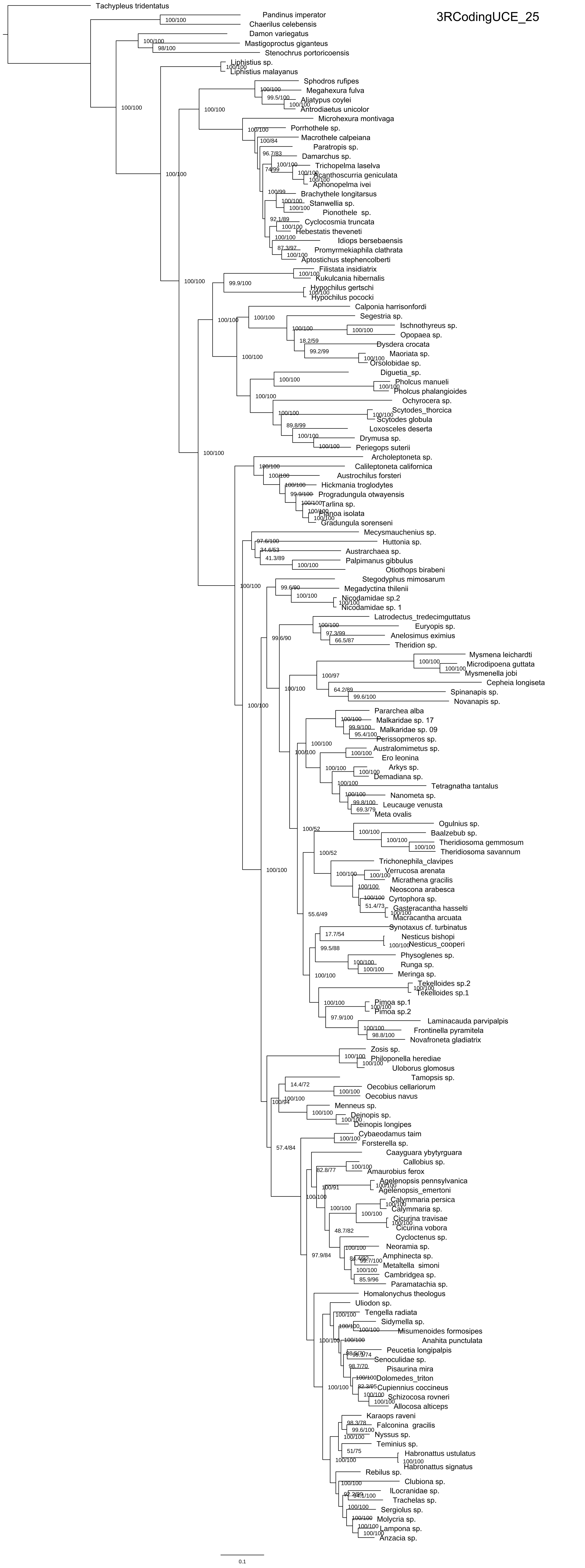
3RCodingUCE_25
Tachypleus tridentatus
Pandinus imperator
Chaerilus celebensis
Damon variegatus
Mastigoproctus giganteus
Stenochrus portoricoensis
Liphistius sp.
Liphistius malayanus
Sphodros rufipes
Megahexura fulva
Aliatypus coylei
Antrodiaetus unicolor
Microhexura montivaga
Porrhothele sp.
Macrothele calpeiana
Paratropis sp.
Damarchus sp.
Trichopelma laselva
Acanthoscurria geniculata
Aphonopelma ivei
Brachythele longitarsus
Stanwellia sp.
Pionothele sp.
Cyclocosmia truncata
Hebestatis theveneti
Idiops bersebaensis
Promyrmekiaphila clathrata
Aptostichus stephencolberti
Filistata insidiatrix
Kukulcania hibernalis
Hypochilus gertschi
Hypochilus pococki
Calponia harrisonfordi
Segestria sp.
Ischnothyreus sp.
Opopaea sp.
Dysdera crocata
Maoriata sp.
Orsolobidae sp.
Diguetia_sp.
Pholcus manueli
Pholcus phalangioides
Ochyrocera sp.
Scytodes_thorcica
Scytodes globula
Loxosceles deserta
Drymusa sp.
Periegops suterii
Archoleptoneta sp.
Calileptoneta californica
Austrochilus forsteri
Hickmania troglodytes
Progradungula otwayensis
Tarlina sp.
Pianoa isolata
Gradungula sorenseni
Mecysmauchenius sp.
Huttonia sp.
Austrarchaea sp.
Palpimanus gibbulus
Otiothops birabeni
Stegodyphus mimosarum
Megadyctina thilenii
Nicodamidae sp.2
Nicodamidae sp. 1
Latrodectus_tredecimguttatus
Euryopis sp.
Anelosimus eximius
Theridion sp.
Mysmena leichardti
Microdipoena guttata
Mysmenella jobi
Cepheia longiseta
Spinanapis sp.
Novanapis sp.
Pararchea alba
Malkaridae sp. 17
Malkaridae sp. 09
Perissopmeros sp.
Australomimetus sp.
Ero leonina
Arkys sp.
Demadiana sp.
Tetragnatha tantalus
Nanometa sp.
Leucauge venusta
Meta ovalis
Ogulnius sp.
Baalzebub sp.
Theridiosoma gemmosum
Theridiosoma savannum
Trichonephila_clavipes
Verrucosa arenata
Micrathena gracilis
Neoscona arabesca
Cyrtophora sp.
Gasteracantha hasselti
Macracantha arcuata
Synotaxus cf. turbinatus
Nesticus bishopi
Nesticus_cooperi
Physoglenes sp.
Runga sp.
Meringa sp.
Tekelloides sp.2
Tekelloides sp.1
Pimoa sp.1
Pimoa sp.2
Laminacauda parvipalpis
Frontinella pyramitela
Novafroneta gladiatrix
Zosis sp.
Philoponella herediae
Uloborus glomosus
Tamopsis sp.
Oecobius cellariorum
Oecobius navus
Menneus sp.
Deinopis sp.
Deinopis longipes
Cybaeodamus taim
Forsterella sp.
Caayguara ybytyrguara
Callobius sp.
Amaurobius ferox
Agelenopsis pennsylvanica
Agelenopsis_emertoni
Calymmaria persica
Calymmaria sp.
Cicurina travisae
Cicurina vobora
Cycloctenus sp.
Neoramia sp.
Amphinecta sp.
Metaltella simoni
Cambridgea sp.
Paramatachia sp.
Homalonychus theologus
Uliodon sp.
Tengella radiata
Sidymella sp.
Misumenoides formosipes
Anahita punctulata
Peucetia longipalpis
Senoculidae sp.
Pisaurina mira
Dolomedes_triton
Cupiennius coccineus
Schizocosa rovneri
Allocosa alticeps
Karaops raveni
Falconina gracilis
Nyssus sp.
Teminius sp.
Habronattus ustulatus
Habronattus signatus
Rebilus sp.
Clubiona sp.
ILocranidae sp.
Trachelas sp.
Sergiolus sp.
Molycria sp.
Lampona sp.
Anzacia sp.
0.1

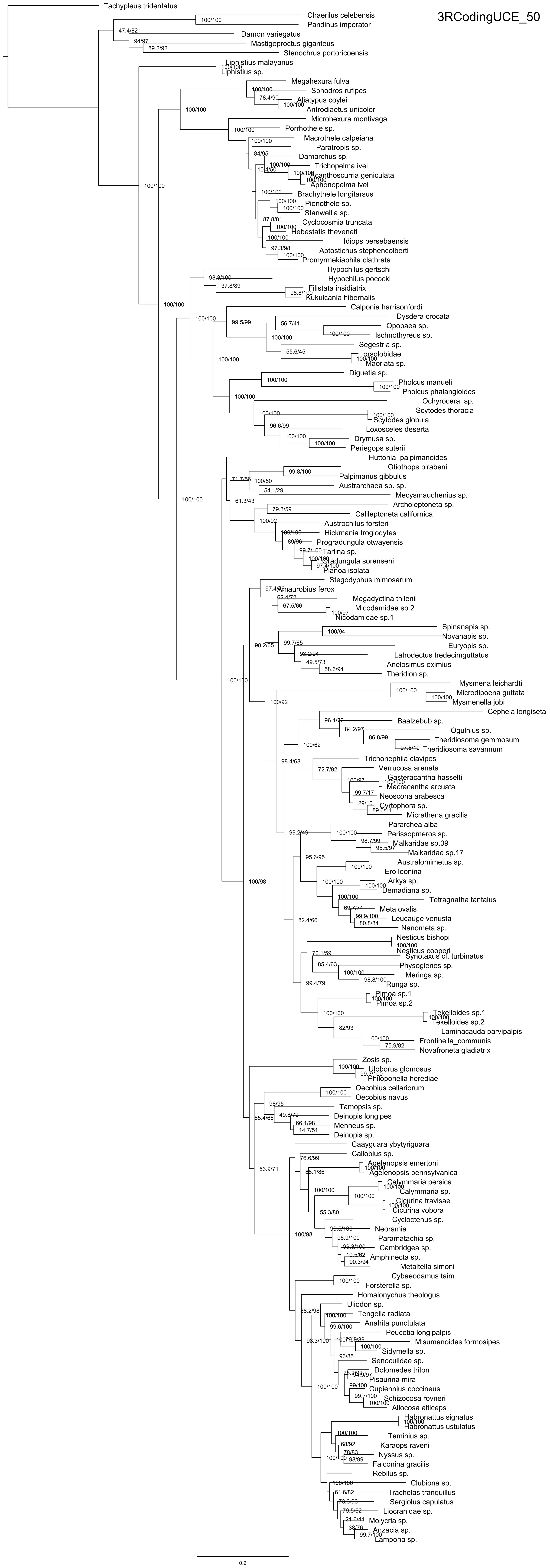

3RCodingUCE_50
Tachypleus tridentatus
Chaerilus celebensis
Pandinus imperator
Damon variegatus
Mastigoproctus giganteus
Stenochrus portoricoensis
Liphistius malayanus
Liphistius sp.
Megahexura fulva
Sphodros rufipes
Aliatypus coylei
Antrodiaetus unicolor
Microhexura montivaga
Porrhothele sp.
Macrothele calpeiana
Paratropis sp.
Damarchus sp.
Trichopelma ivei
Acanthoscurria geniculata
Aphonopelma ivei
Brachythele longitarsus
Pionothele sp.
Stanwellia sp.
Cyclocosmia truncata
Hebestatis theveneti
Idiops bersebaensis
Aptostichus stephencolberti
Promyrmekiaphila clathrata
Hypochilus gertschi
Hypochilus pococki
Filistata insidiatrix
Kukulcania hibernalis
Calponia harrisonfordi
Dysdera crocata
Opopaea sp.
Ischnothyreus sp.
Segestria sp.
orsolobidae
Maoriata sp.
Diguetia sp.
Pholcus manueli
Pholcus phalangioides
Ochyrocera sp.
Scytodes thoracia
Scytodes globula
Loxosceles deserta
Drymusa sp.
Periegops suterii
Huttonia palpimanoides
Otiothops birabeni
Palpimanus gibbulus
Austrarchaea sp. sp.
Mecysmauchenius sp.
Archoleptoneta sp.
Calileptoneta californica
Austrochilus forsteri
Hickmania troglodytes
Progradungula otwayensis
Tarlina sp.
Gradungula sorenseni
Pianoa isolata
Stegodyphus mimosarum
Amaurobius ferox
Megadyctina thilenii
Micodamidae sp.2
Nicodamidae sp.1
Spinanapis sp.
Novanapis sp.
Euryopis sp.
Latrodectus tredecimguttatus
Anelosimus eximius
Theridion sp.
Mysmena leichardti
Microdipoena guttata
Mysmenella jobi
Cepheia longiseta
Baalzebub sp.
Ogulnius sp.
Theridiosoma gemmosum
Theridiosoma savannum
Trichonephila clavipes
Verrucosa arenata
Gasteracantha hasselti
Macracantha arcuata
Neoscona arabesca
Cyrtophora sp.
Micrathena gracilis
Pararchea alba
Perissopmeros sp.
Malkaridae sp.09
Malkaridae sp.17
Australomimetus sp.
Ero leonina
Arkys sp.
Demadiana sp.
Tetragnatha tantalus
Meta ovalis
Leucauge venusta
Nanometa sp.
Nesticus bishopi
Nesticus cooperi
Synotaxus cf. turbinatus
Physoglenes sp.
Meringa sp.
Runga sp.
Pimoa sp.1
Pimoa sp.2
Tekelloides sp.1
Tekelloides sp.2
Laminacauda parvipalpis
Frontinella_communis
Novafroneta gladiatrix
Zosis sp.
Uloborus glomosus
Philoponella herediae
Oecobius cellariorum
Oecobius navus
Tamopsis sp.
Deinopis longipes
Menneus sp.
Deinopis sp.
Caayguara ybytyriguara
Callobius sp.
Agelenopsis emertoni
Agelenopsis pennsylvanica
Calymmaria persica
Calymmaria sp.
Cicurina travisae
Cicurina vobora
Cycloctenus sp.
Neoramia
Paramatachia sp.
Cambridgea sp.
Amphinecta sp.
Metaltella simoni
Cybaeodamus taim
Forsterella sp.
Homalonychus theologus
Uliodon sp.
Tengella radiata
Anahita punctulata
Peucetia longipalpis
Misumenoides formosipes
Sidymella sp.
Senoculidae sp.
Dolomedes triton
Pisaurina mira
Cupiennius coccineus
Schizocosa rovneri
Allocosa alticeps
Habronattus signatus
Habronattus ustulatus
Teminius sp.
Karaops raveni
Nyssus sp.
Falconina gracilis
Rebilus sp.
Clubiona sp.
Trachelas tranquillus
Sergiolus capulatus
Liocranidae sp.
Molycria sp.
Anzacia sp.
Lampona sp.
0.2

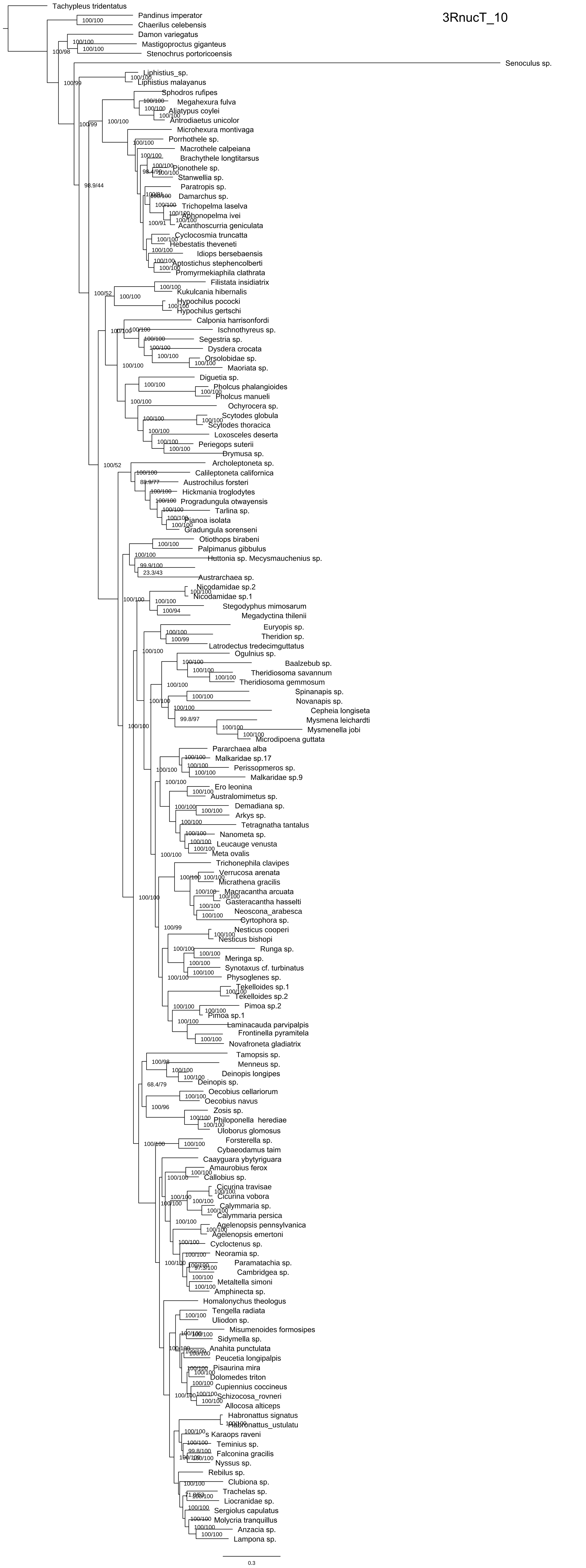
3RnucT_10
Tachypleus tridentatus
Pandinus imperator
Chaerilus celebensis
Damon variegatus
Mastigoproctus giganteus
Stenochrus portoricoensis
Senoculus sp.
Liphistius_sp.
Liphistius malayanus
Sphodros rufipes
Megahexura fulva
Aliatypus coylei
Antrodiaetus unicolor
Microhexura montivaga
Porrhothele sp.
Macrothele calpeiana
Brachythele longtitarsus
Pionothele sp.
Stanwellia sp.
Paratropis sp.
Damarchus sp.
Trichopelma laselva
Aphonopelma ivei
Acanthoscurria geniculata
Cyclocosmia truncatta
Hebestatis theveneti
Idiops bersebaensis
Aptostichus stephencolberti
Promyrmekiaphila clathrata
Filistata insidiatrix
Kukulcania hibernalis
Hypochilus pococki
Hypochilus gertschi
Calponia harrisonfordi
Ischnothyreus sp.
Segestria sp.
Dysdera crocata
Orsolobidae sp.
Maoriata sp.
Diguetia sp.
Pholcus phalangioides
Pholcus manueli
Ochyrocera sp.
Scytodes globula
Scytodes thoracica
Loxosceles deserta
Periegops suterii
Drymusa sp.
Archoleptoneta sp.
Calileptoneta californica
Austrochilus forsteri
Hickmania troglodytes
Progradungula otwayensis
Tarlina sp.
Pianoa isolata
Gradungula sorenseni
Otiothops birabeni
Palpimanus gibbulus
Huttonia sp. Mecysmauchenius sp.
Austrarchaea sp.
Nicodamidae sp.2
Nicodamidae sp.1
Stegodyphus mimosarum
Megadyctina thilenii
Euryopis sp.
Theridion sp.
Latrodectus tredecimguttatus
Ogulnius sp.
Baalzebub sp.
Theridiosoma savannum
Theridiosoma gemmosum
Spinanapis sp.
Novanapis sp.
Cepheia longiseta
Mysmena leichardti
Mysmenella jobi
Microdipoena guttata
Pararchaea alba
Malkaridae sp.17
Perissopmeros sp.
Malkaridae sp.9
Ero leonina
Australomimetus sp.
Demadiana sp.
Arkys sp.
Tetragnatha tantalus
Nanometa sp.
Leucauge venusta
Meta ovalis
Trichonephila clavipes
Verrucosa arenata
Micrathena gracilis
Macracantha arcuata
Gasteracantha hasselti
Neoscona_arabesca
Cyrtophora sp.
Nesticus cooperi
Nesticus bishopi
Runga sp.
Meringa sp.
Synotaxus cf. turbinatus
Physoglenes sp.
Tekelloides sp.1
Tekelloides sp.2
Pimoa sp.2
Pimoa sp.1
Laminacauda parvipalpis
Frontinella pyramitela
Novafroneta gladiatrix
Tamopsis sp.
Menneus sp.
Deinopis longipes
Deinopis sp.
Oecobius cellariorum
Oecobius navus
Zosis sp.
Philoponella herediae
Uloborus glomosus
Forsterella sp.
Cybaeodamus taim
Caayguara ybytyriguara
Amaurobius ferox
Callobius sp.
Cicurina travisae
Cicurina vobora
Calymmaria sp.
Calymmaria persica
Agelenopsis pennsylvanica
Agelenopsis emertoni
Cycloctenus sp.
Neoramia sp.
Paramatachia sp.
Cambridgea sp.
Metaltella simoni
Amphinecta sp.
Homalonychus theologus
Tengella radiata
Uliodon sp.
Misumenoides formosipes
Sidymella sp.
Anahita punctulata
Peucetia longipalpis
Pisaurina mira
Dolomedes triton
Cupiennius coccineus
Schizocosa_rovneri
Allocosa alticeps
Habronattus signatus
Habronattus_ustulatu
s Karaops raveni
Teminius sp.
Falconina gracilis
Nyssus sp.
Rebilus sp.
Clubiona sp.
Trachelas sp.
Liocranidae sp.
Sergiolus capulatus
Molycria tranquillus
Anzacia sp.
Lampona sp.
0.3

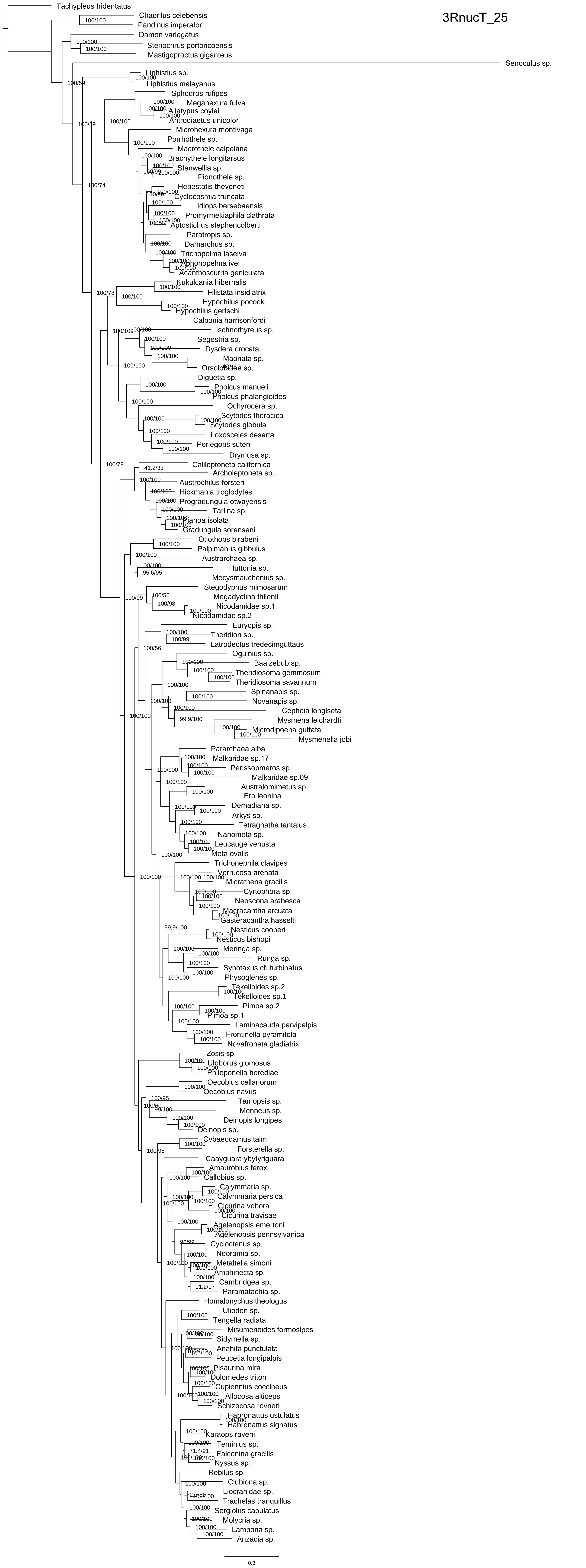
3RnucT_25
Tachypleus tridentatus
Chaerilus celebensis
Pandinus imperator
Damon variegatus
Stenochrus portoricoensis
Mastigoproctus giganteus
Senoculus sp.
Liphistius sp.
Liphistius malayanus
Sphodros rufipes
Megahexura fulva
Aliatypus coylei
Antrodiaetus unicolor
Microhexura montivaga
Porrhothele sp.
Macrothele calpeiana
Brachythele longitarsus
Stanwellia sp.
Pionothele sp.
Hebestatis theveneti
Cyclocosmia truncata
Idiops bersebaensis
Promyrmekiaphila clathrata
Aptostichus stephencolberti
Paratropis sp.
Damarchus sp.
Trichopelma laselva
Aphonopelma ivei
Acanthoscurria geniculata
Kukulcania hibernalis
Filistata insidiatrix
Hypochilus pococki
Hypochilus gertschi
Calponia harrisonfordi
Ischnothyreus sp.
Segestria sp.
Dysdera crocata
Maoriata sp.
Orsolobidae sp.
Diguetia sp.
Pholcus manueli
Pholcus phalangioides
Ochyrocera sp.
Scytodes thoracica
Scytodes globula
Loxosceles deserta
Periegops suterii
Drymusa sp.
Calileptoneta californica
Archoleptoneta sp.
Austrochilus forsteri
Hickmania troglodytes
Progradungula otwayensis
Tarlina sp.
Pianoa isolata
Gradungula sorenseni
Otiothops birabeni
Palpimanus gibbulus
Austrarchaea sp.
Huttonia sp.
Mecysmauchenius sp.
Stegodyphus mimosarum
Megadyctina thilenii
Nicodamidae sp.1
Nicodamidae sp.2
Euryopis sp.
Theridion sp.
Latrodectus tredecimguttaus
Ogulnius sp.
Baalzebub sp.
Theridiosoma gemmosum
Theridiosoma savannum
Spinanapis sp.
Novanapis sp.
Cepheia longiseta
Mysmena leichardti
Microdipoena guttata
Mysmenella jobi
Pararchaea alba
Malkaridae sp.17
Perissopmeros sp.
Malkaridae sp.09
Australomimetus sp.
Ero leonina
Demadiana sp.
Arkys sp.
Tetragnatha tantalus
Nanometa sp.
Leucauge venusta
Meta ovalis
Trichonephila clavipes
Verrucosa arenata
Micrathena gracilis
Cyrtophora sp.
Neoscona arabesca
Macracantha arcuata
Gasteracantha hasselti
Nesticus cooperi
Nesticus bishopi
Meringa sp.
Runga sp.
Synotaxus cf. turbinatus
Physoglenes sp.
Tekelloides sp.2
Tekelloides sp.1
Pimoa sp.2
Pimoa sp.1
Laminacauda parvipalpis
Frontinella pyramitela
Novafroneta gladiatrix
Zosis sp.
Uloborus glomosus
Philoponella herediae
Oecobius cellariorum
Oecobius navus
Tamopsis sp.
Menneus sp.
Deinopis longipes
Deinopis sp.
Cybaeodamus taim
Forsterella sp.
Caayguara ybytyriguara
Amaurobius ferox
Callobius sp.
Calymmaria sp.
Calymmaria persica
Cicurina vobora
Cicurina travisae
Agelenopsis emertoni
Agelenopsis pennsylvanica
Cycloctenus sp.
Neoramia sp.
Metaltella simoni
Amphinecta sp.
Cambridgea sp.
Paramatachia sp.
Homalonychus theologus
Uliodon sp.
Tengella radiata
Misumenoides formosipes
Sidymella sp.
Anahita punctulata
Peucetia longipalpis
Pisaurina mira
Dolomedes triton
Cupiennius coccineus
Allocosa alticeps
Schizocosa rovneri
Habronattus ustulatus
Habronattus signatus
Karaops raveni
Teminius sp.
Falconina gracilis
Nyssus sp.
Rebilus sp.
Clubiona sp.
Liocranidae sp.
Trachelas tranquillus
Sergiolus capulatus
Molycria sp.
Lampona sp.
Anzacia sp.
0.3

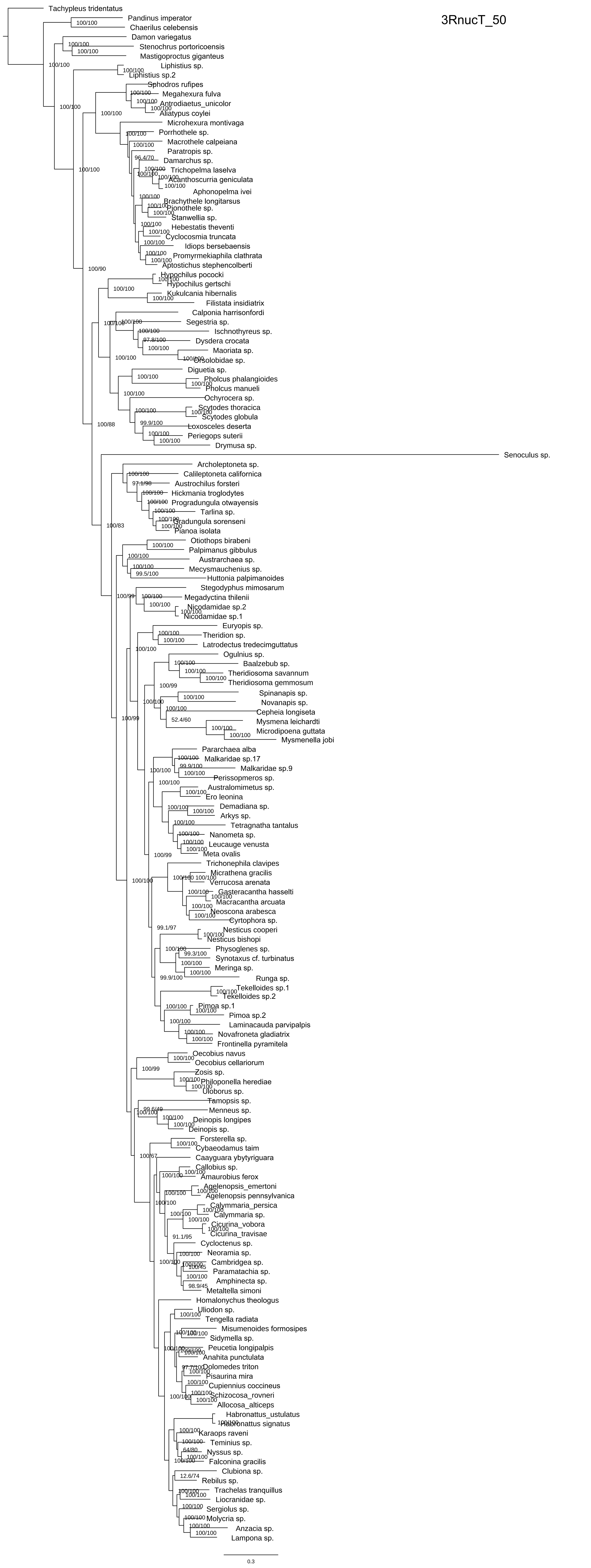
3RnucT_50
0.3

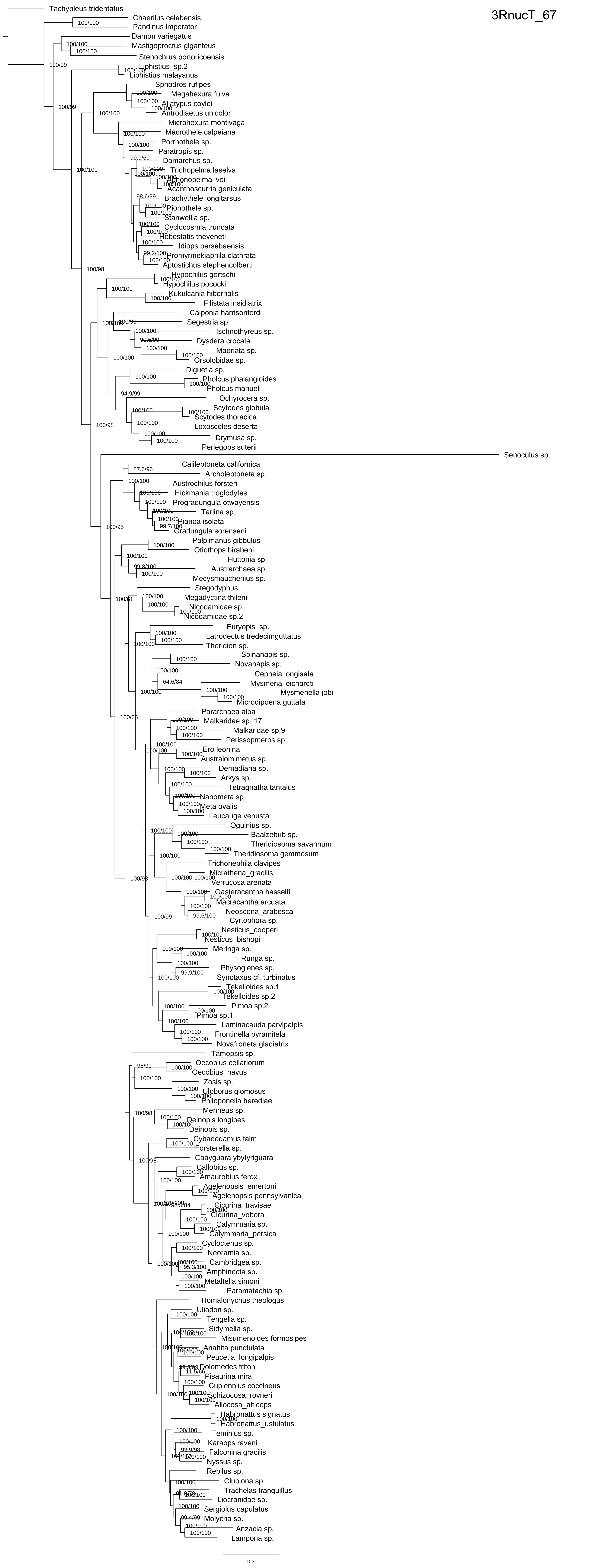
3RnucT_67
Tachypleus tridentatus
Chaerilus celebensis
Pandinus imperator
Damon variegatus
Mastigoproctus giganteus
Stenochrus portoricoensis
Liphistius_sp.2
Liphistius malayanus
Sphodros rufipes
Megahexura fulva
Aliatypus coylei
Antrodiaetus unicolor
Microhexura montivaga
Macrothele calpeiana
Porrhothele sp.
Paratropis sp.
Damarchus sp.
Trichopelma laselva
Aphonopelma ivei
Acanthoscurria geniculata
Brachythele longitarsus
Pionothele sp.
Stanwellia sp.
Cyclocosmia truncata
Hebestatis theveneti
Idiops bersebaensis
Promyrmekiaphila clathrata
Aptostichus stephencolberti
Hypochilus gertschi
Hypochilus pococki
Kukulcania hibernalis
Filistata insidiatrix
Calponia harrisonfordi
Segestria sp.
Ischnothyreus sp.
Dysdera crocata
Maoriata sp.
Orsolobidae sp.
Diguetia sp.
Pholcus phalangioides
Pholcus manueli
Ochyrocera sp.
Scytodes globula
Scytodes thoracica
Loxosceles deserta
Drymusa sp.
Periegops suterii
Senoculus sp.
Calileptoneta californica
Archoleptoneta sp.
Austrochilus forsteri
Hickmania troglodytes
Progradungula otwayensis
Tarlina sp.
Pianoa isolata
Gradungula sorenseni
Palpimanus gibbulus
Otiothops birabeni
Huttonia sp.
Austrarchaea sp.
Mecysmauchenius sp.
Stegodyphus
Megadyctina thilenii
Nicodamidae sp.
Nicodamidae sp.2
Euryopis sp.
Latrodectus tredecimguttatus
Theridion sp.
Spinanapis sp.
Novanapis sp.
Cepheia longiseta
Mysmena leichardti
Mysmenella jobi
Microdipoena guttata
Pararchaea alba
Malkaridae sp. 17
Malkaridae sp.9
Perissopmeros sp.
Ero leonina
Australomimetus sp.
Demadiana sp.
Arkys sp.
Tetragnatha tantalus
Nanometa sp.
Meta ovalis
Leucauge venusta
Ogulnius sp.
Baalzebub sp.
Theridiosoma savannum
Theridiosoma gemmosum
Trichonephila clavipes
Micrathena_gracilis
Verrucosa arenata
Gasteracantha hasselti
Macracantha arcuata
Neoscona_arabesca
Cyrtophora sp.
Nesticus_cooperi
Nesticus_bishopi
Meringa sp.
Runga sp.
Physoglenes sp.
Synotaxus cf. turbinatus
Tekelloides sp.1
Tekelloides sp.2
Pimoa sp.2
Pimoa sp.1
Laminacauda parvipalpis
Frontinella pyramitela
Novafroneta gladiatrix
Tamopsis sp.
Oecobius cellariorum
Oecobius_navus
Zosis sp.
Uloborus glomosus
Philoponella herediae
Menneus sp.
Deinopis longipes
Deinopis sp.
Cybaeodamus taim
Forsterella sp.
Caayguara ybytyriguara
Callobius sp.
Amaurobius ferox
Agelenopsis_emertoni
Agelenopsis pennsylvanica
Cicurina_travisae
Cicurina_vobora
Calymmaria sp.
Calymmaria_persica
Cycloctenus sp.
Neoramia sp.
Cambridgea sp.
Amphinecta sp.
Metaltella simoni
Paramatachia sp.
Homalonychus theologus
Uliodon sp.
Tengella sp.
Sidymella sp.
Misumenoides formosipes
Anahita punctulata
Peucetia_longipalpis
Dolomedes triton
Pisaurina mira
Cupiennius coccineus
Schizocosa_rovneri
Allocosa_alticeps
Habronattus signatus
Habronattus_ustulatus
Teminius sp.
Karaops raveni
Falconina gracilis
Nyssus sp.
Rebilus sp.
Clubiona sp.
Trachelas tranquillus
Liocranidae sp.
Sergiolus capulatus
Molycria sp.
Anzacia sp.
Lampona sp.
0.3
